# Supplementary material for: Mapping the landscape of histomorphological cancer phenotypes using self-supervised learning on unannotated pathology slides
Source: Nat Commun. 2024 Jun 11;15:4596. doi: 10.1038/s41467-024-48666-7 (PMC11525555; doi:10.1038/s41467-024-48666-7)
Supplement: Supplementary file 1 — Supplementary Information [file 41467_2024_48666_MOESM1_ESM.pdf]

Supplementary Information

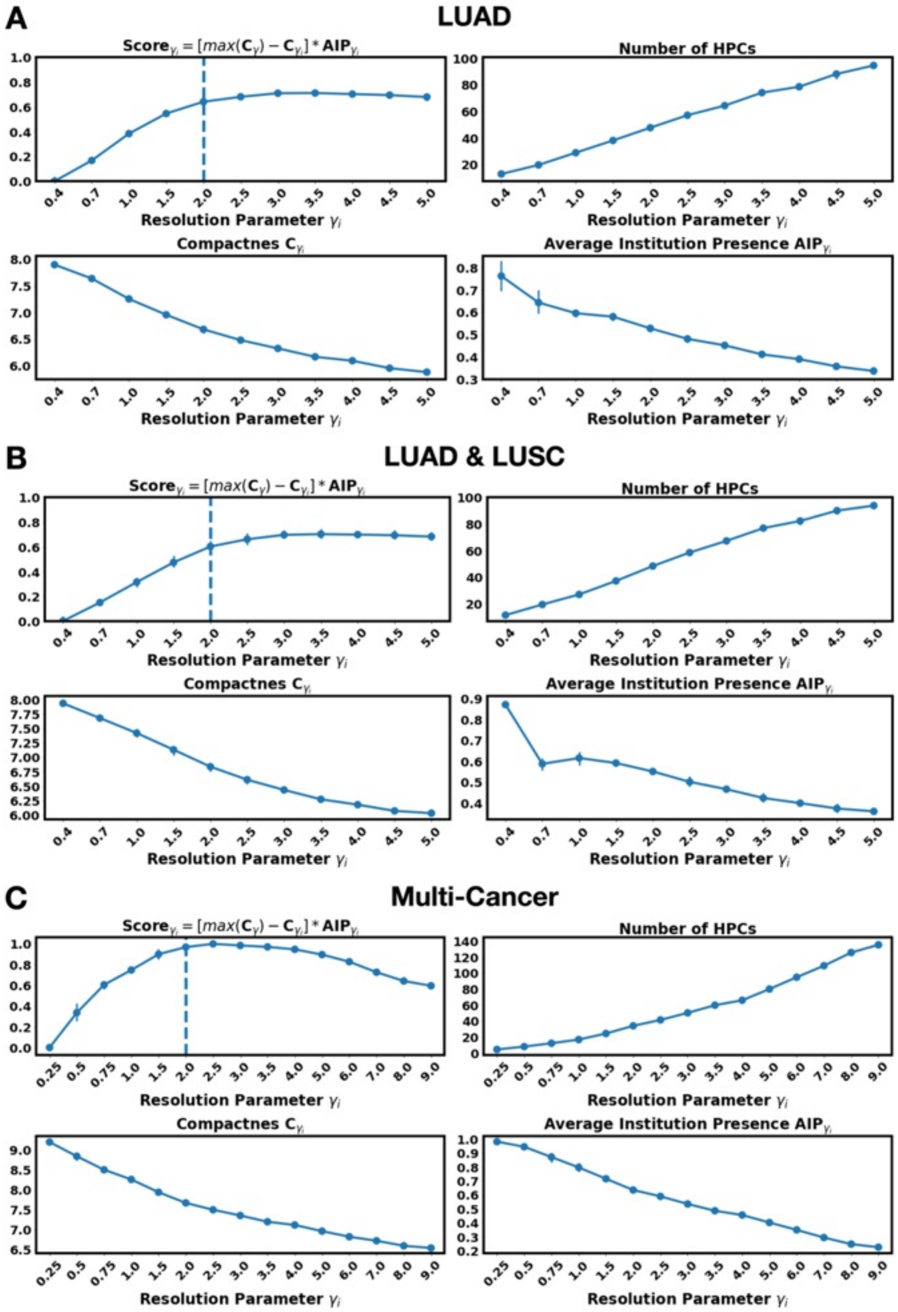

Supplementary Figure 1. Selection of number of HPCs. Graphs are shown for **A** the LUAD study, **B** the LUAD vs LUSC classification task and **C** the multi-cancer analysis. For each of them, we shop the final score corresponding to **Equation 6** (top left panel), the resulting number of HPCs for each leiden resolution (top right), and the two trade-off elements forming the final score: the compactness parameters associated with **Equation 4** (bottom left) and the average presence of institutions per HPC from **Equation 5**. The dotted line shows the optimal Leiden resolution. We selected the optimal Leiden resolution where the score function shows a larger trend change and begins saturation (elbow method or knee of a curve). Source data are provided as a Source Data file.

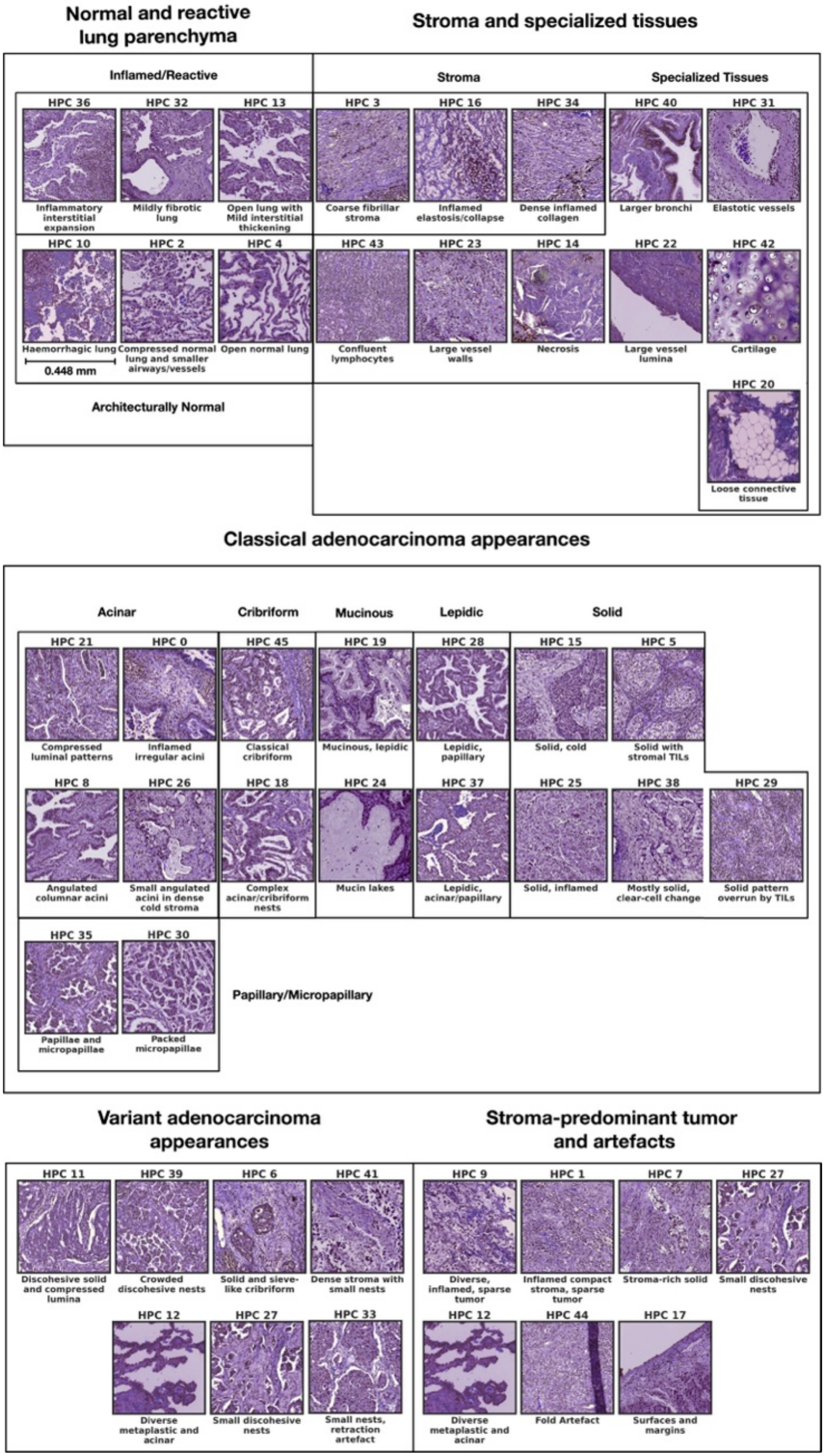

Supplementary Figure 2. Consensus description of HPCs with their representative tiles. Source data are provided as a Source Data file.

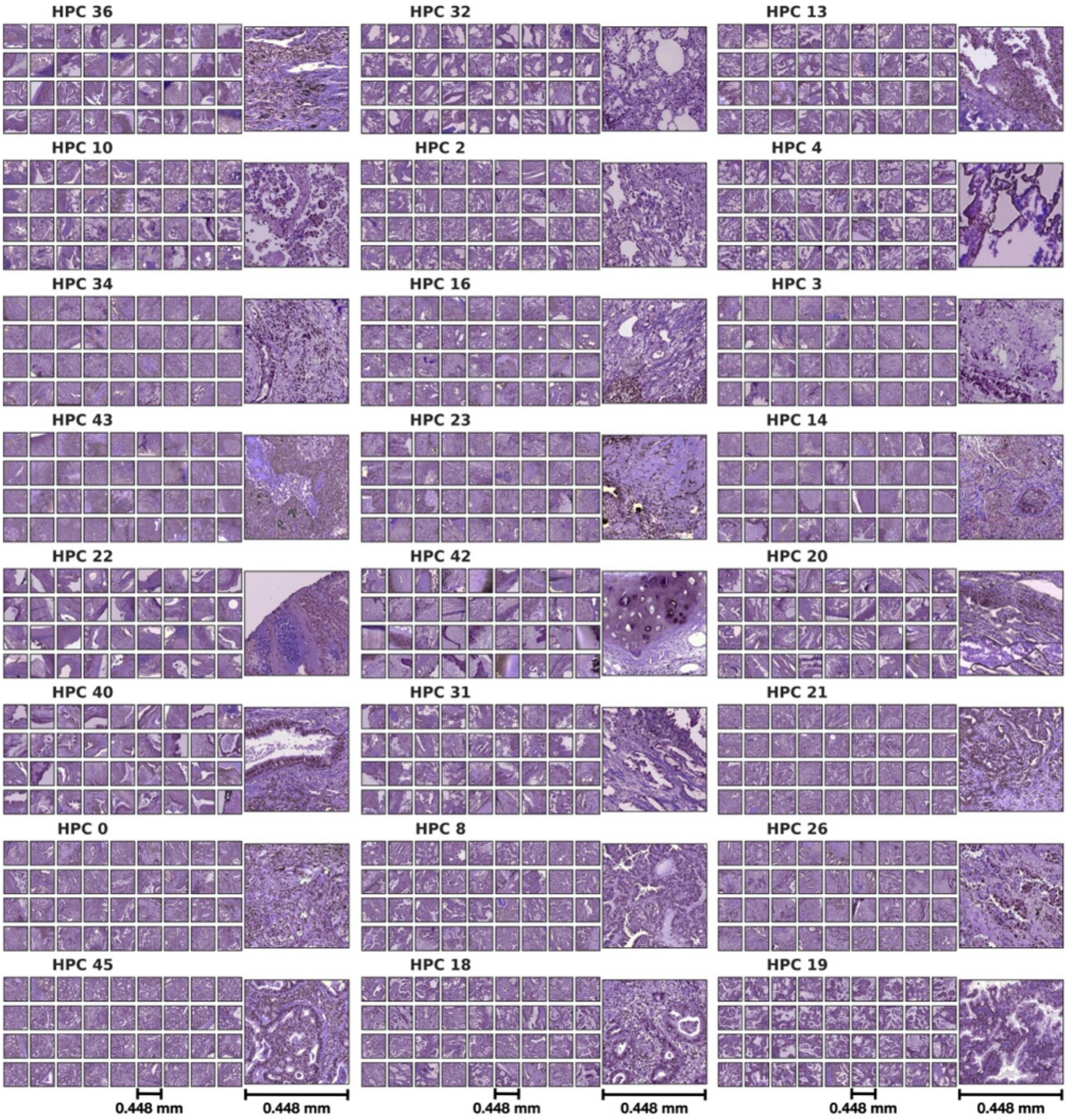

Supplementary Figure 3. More examples of tiles from HPC discussed in Figures 3 and 4. Clusters are ordered as they appear in those two main figures. Source data are provided as a Source Data file.

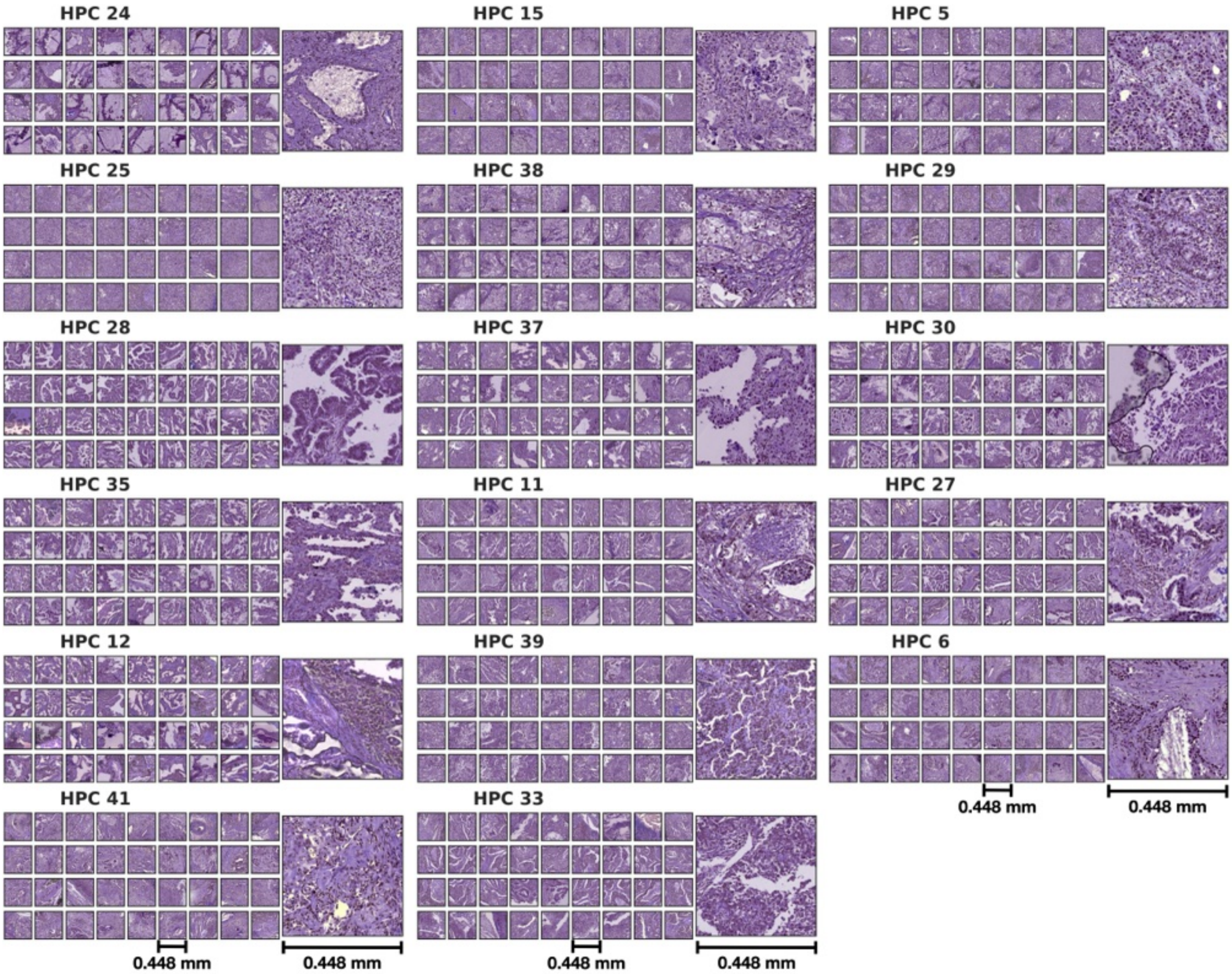

Supplementary Figure 4. More examples of tiles from HPC discussed in Figures 3 and 4. Clusters are ordered as they appear in those two main figures. Source data are provided as a Source Data file.

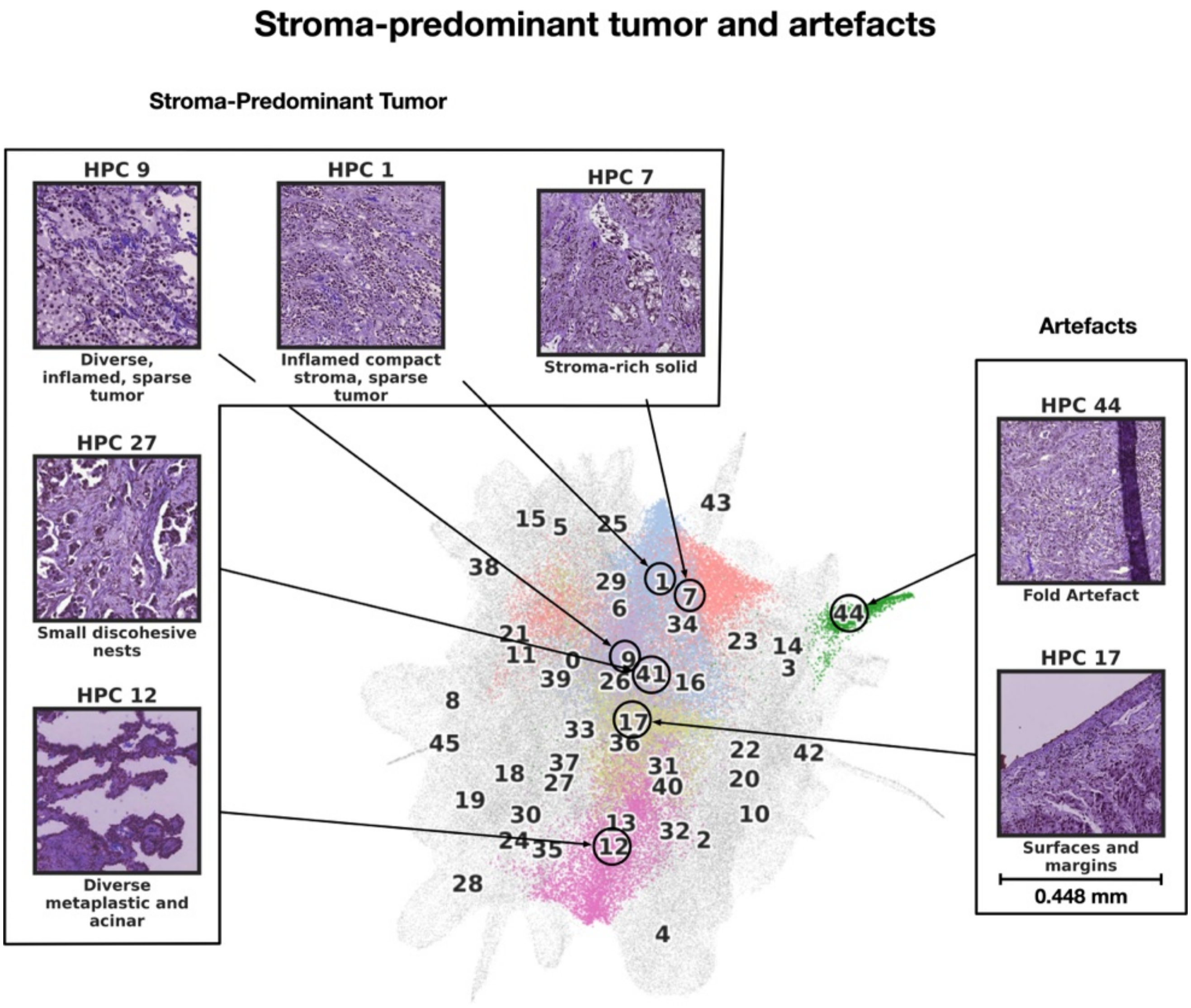

Supplementary Figure 5. Consensus description of HPCs enriched in stroma-predominant tumor and artefacts with their representative tiles). Source data are provided as a Source Data file.

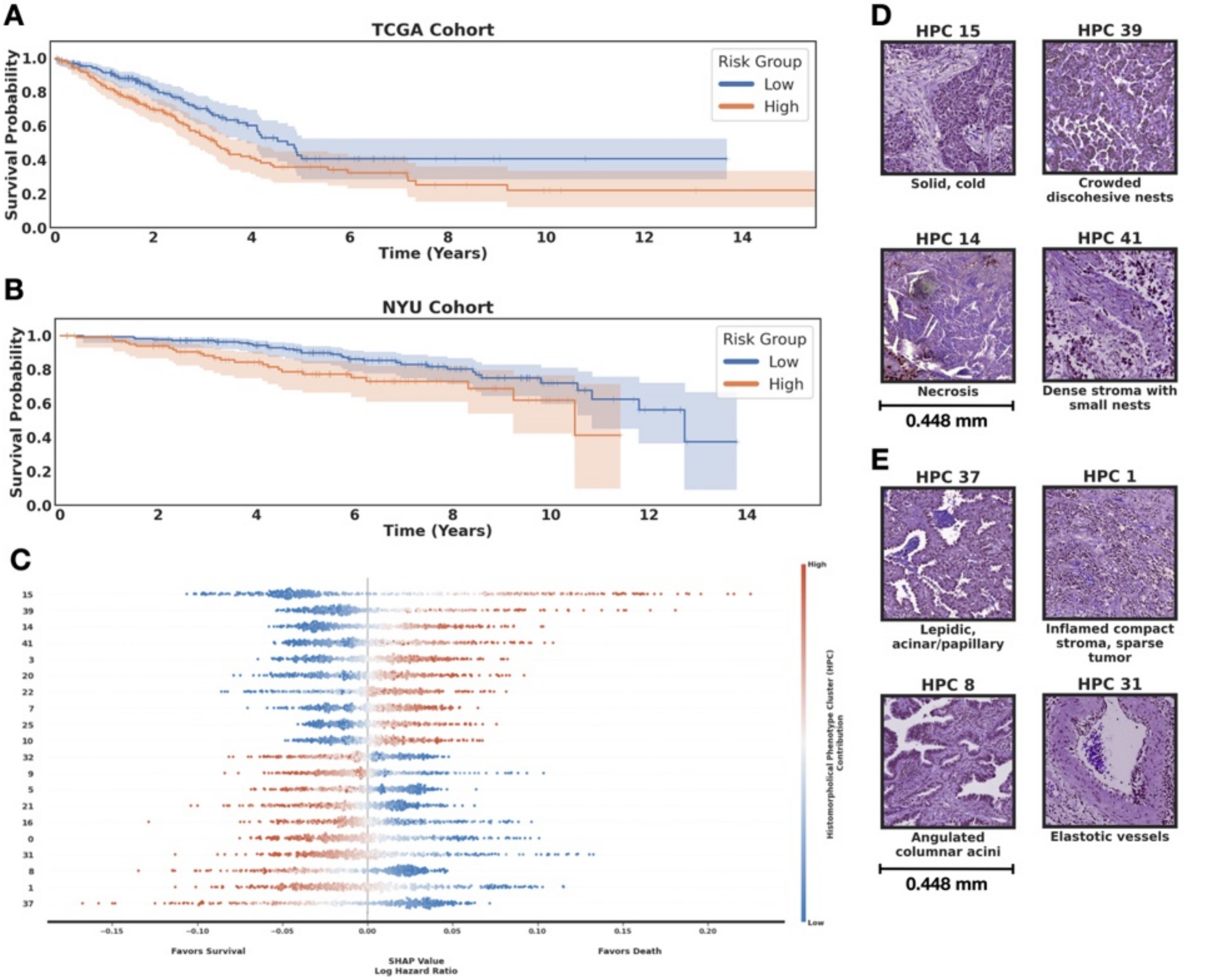

Supplementary Figure 6. Lung adenocarcinoma overall survival. A. SHAP (SHapley Additive exPlanations) plot showing how HPC (y-axis) weight on survival outcome. **B.** High and low risk groups on TCGA cohort showing statistical significance (p-value $3.7 \times 10^{-3} < 0.05$) and c-index of 0.60. **C.** High and low risk groups on NYU_1 cohort showing statistical significance (p-value $1.6 \times 10^{-2} < 0.05$) and c-index of 0.65. **D.** Representative tiles for HPCs associated with poor survival in panel C. **E.** Representative tiles for HPCs associated with good survival in panel C. Source data are provided as a Source Data file.

A

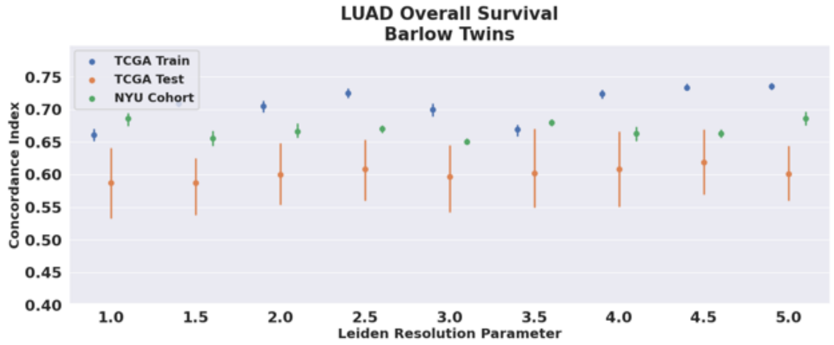

B

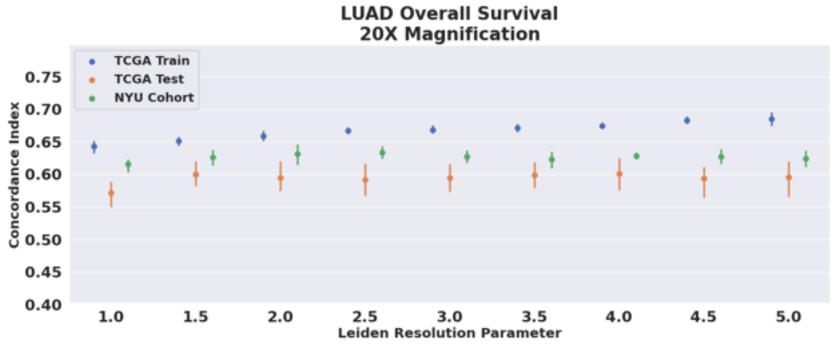

C

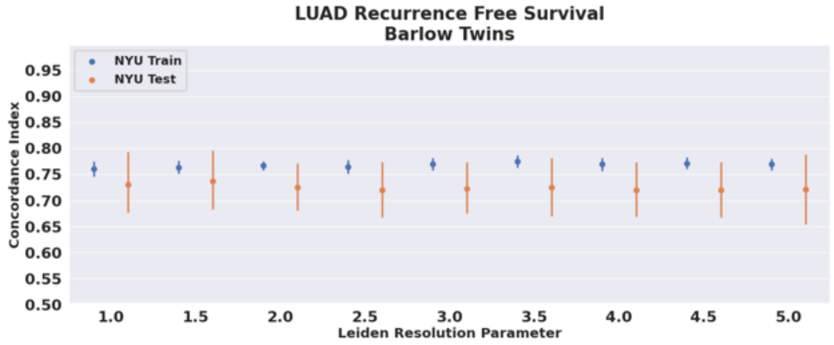

D

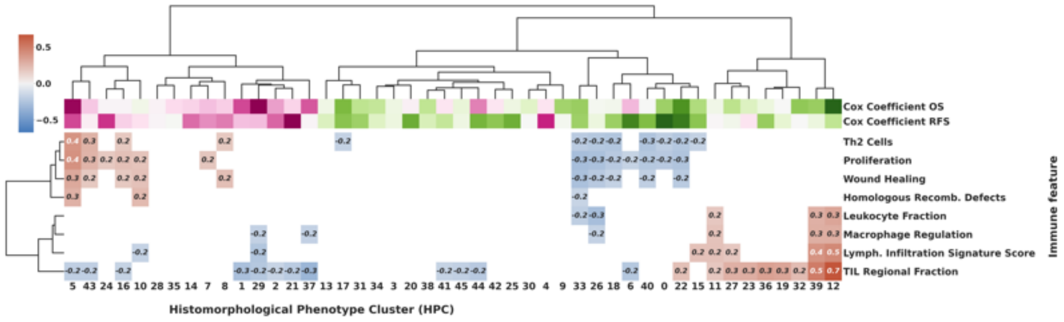

Supplementary Figure 7. Survival analysis at different resolutions and after training Barlow-Twins without sub-sampling the training dataset. **A** OS measured from HPL run at 5x. **B** OS measured from HPL run at 20x. **C** RFS measured from HPL run at 5x. **D** Bi-hierarchical clustering of HPCs and immune signature correlations where positive correlations are shown in red and negative as blue, corresponding to run at 20x and Leiden resolution of 2.0. Cox coefficients for overall and recurrence free survival are colored with a gradient scheme, from favoring death or recurrence as purple to favoring survival or no recurrence as green. All runs were done following a 5-fold cross-validation split similar to the one used in previous studies[1,2]. Source data are provided as a Source Data file.

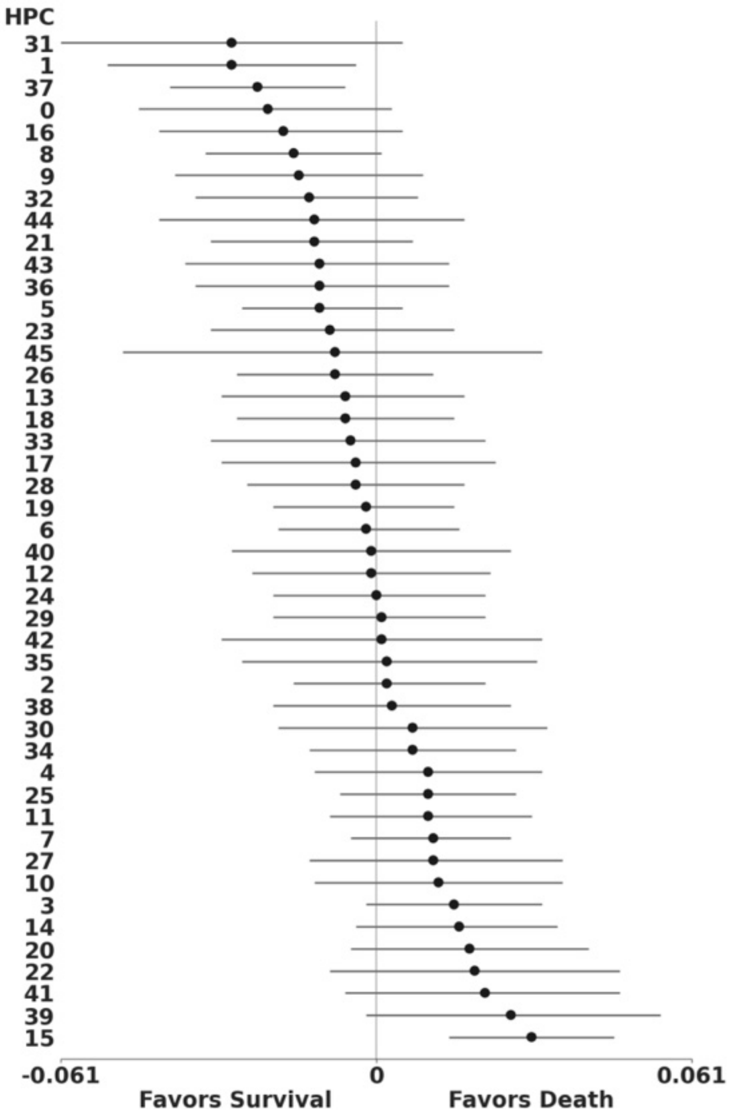

HPC	Log Hazard Ratio	95% CI	P-Value
31	-0.028	(-0.061, 0.005)	0.008
1	-0.028	(-0.052, -0.004)	0.0
37	-0.023	(-0.04, -0.006)	0.0
0	-0.021	(-0.046, 0.003)	0.006
16	-0.018	(-0.042, 0.005)	0.012
8	-0.016	(-0.033, 0.001)	0.001
9	-0.015	(-0.039, 0.009)	0.082
32	-0.013	(-0.035, 0.008)	0.109
44	-0.012	(-0.042, 0.017)	0.509
21	-0.012	(-0.032, 0.007)	0.083
43	-0.011	(-0.037, 0.014)	0.381
36	-0.011	(-0.035, 0.014)	0.407
5	-0.011	(-0.026, 0.005)	0.049
23	-0.009	(-0.032, 0.015)	0.429
45	-0.008	(-0.049, 0.032)	0.85
26	-0.008	(-0.027, 0.011)	0.387
13	-0.006	(-0.03, 0.017)	0.595
18	-0.006	(-0.027, 0.015)	0.772
33	-0.005	(-0.032, 0.021)	0.822
17	-0.004	(-0.03, 0.023)	0.954
28	-0.004	(-0.025, 0.017)	0.952
19	-0.002	(-0.02, 0.015)	0.982
6	-0.002	(-0.019, 0.016)	0.985
40	-0.001	(-0.028, 0.026)	0.994
12	-0.001	(-0.024, 0.022)	0.912
24	0.0	(-0.02, 0.021)	0.99
29	0.001	(-0.02, 0.021)	0.951
42	0.001	(-0.03, 0.032)	0.909
35	0.002	(-0.026, 0.031)	0.993
2	0.002	(-0.016, 0.021)	0.895
38	0.003	(-0.02, 0.026)	0.651
30	0.007	(-0.019, 0.033)	0.614
34	0.007	(-0.013, 0.027)	0.65
4	0.01	(-0.012, 0.032)	0.411
25	0.01	(-0.007, 0.027)	0.122
11	0.01	(-0.009, 0.03)	0.224
7	0.011	(-0.005, 0.026)	0.035
27	0.011	(-0.013, 0.036)	0.378
10	0.012	(-0.012, 0.036)	0.296
3	0.015	(-0.002, 0.032)	0.006
14	0.016	(-0.004, 0.035)	0.02
20	0.018	(-0.005, 0.041)	0.021
22	0.019	(-0.009, 0.047)	0.051
41	0.021	(-0.006, 0.047)	0.015
39	0.026	(-0.002, 0.055)	0.002
15	0.03	(0.014, 0.046)	0.0

Supplementary Figure 8. Lung adenocarcinoma overall survival Forest plot. Log hazard ratio of Cox proportional hazards model over the train sets of a 5-fold cross-validation over TCGA data and NYU as independent cohort. We averaged coefficients across fold and combined p-values with Fisher's combined probability test. Statistically significant HPCs (p-value$<$ 0.05) and HPCs that contain at least 10% of total patients are in bold, which is motivated by finding tissue patterns that can generalize across the cohort. This plot shows the relationship between HPCs and their relevance in predicting overall survival, negative values show association with survival while positive values are associated with a death event. Source data are provided as a Source Data file.

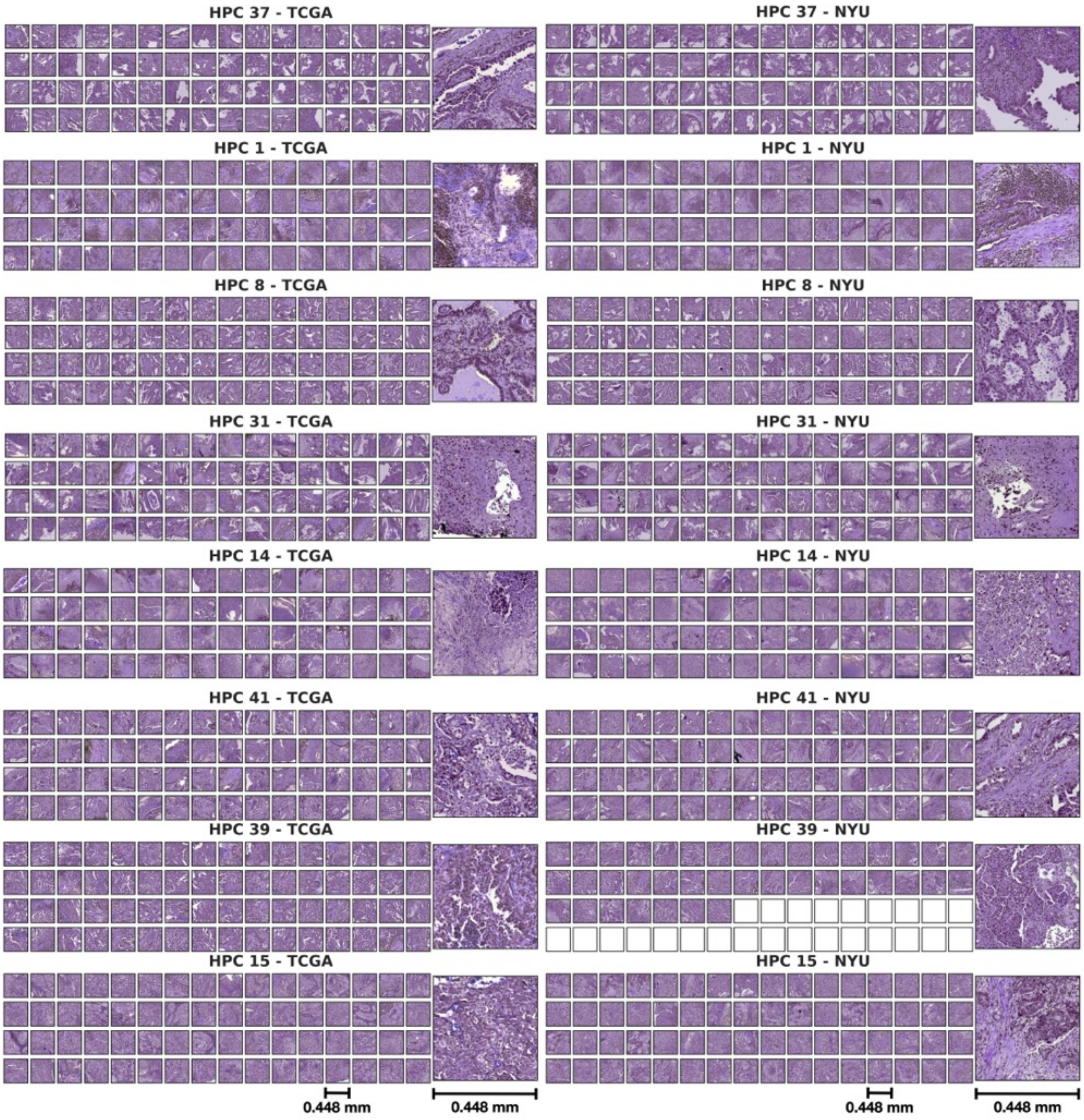

Supplementary Figure 9. The Cancer Genome Atlas (TCGA) and New York University NYU_1 **tile samples for statistically significant HPCs on lung adenocarcinoma (LUAD) overall survival.** Source data are provided as a Source Data file.

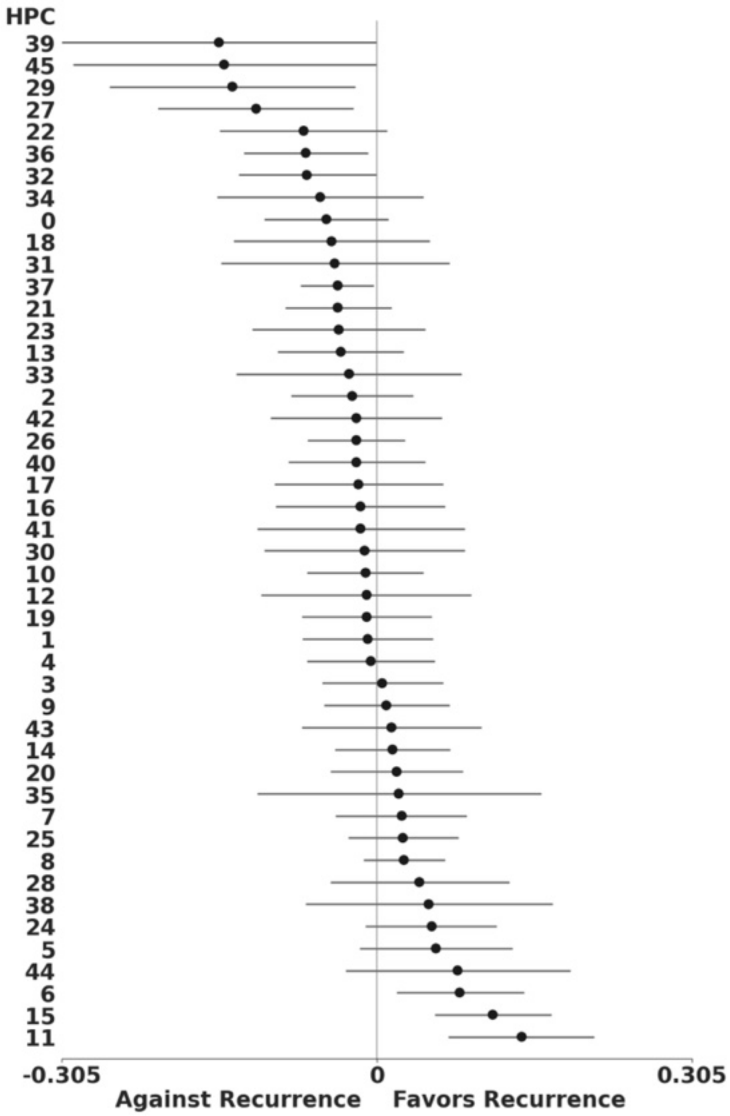

HPC	Log Hazard Ratio	95% CI	P-Value
39	-0.153	(-0.305, -0.001)	0.0
45	-0.148	(-0.294, -0.001)	0.0
29	**-0.14**	**(-0.259, -0.021)**	**0.0**
27	**-0.117**	**(-0.212, -0.023)**	**0.0**
22	**-0.071**	**(-0.152, 0.01)**	**0.006**
36	**-0.069**	**(-0.129, -0.009)**	**0.0**
32	**-0.068**	**(-0.134, -0.001)**	**0.0**
34	-0.055	(-0.155, 0.045)	0.211
0	**-0.049**	**(-0.109, 0.011)**	**0.009**
18	-0.044	(-0.139, 0.051)	0.393
31	-0.041	(-0.151, 0.07)	0.624
37	**-0.038**	**(-0.074, -0.003)**	**0.0**
21	**-0.038**	**(-0.089, 0.014)**	**0.039**
23	-0.037	(-0.121, 0.047)	0.417
13	-0.035	(-0.096, 0.026)	0.146
33	-0.027	(-0.136, 0.082)	0.596
2	-0.024	(-0.083, 0.035)	0.551
42	-0.02	(-0.103, 0.063)	0.876
26	-0.02	(-0.067, 0.027)	0.46
40	-0.02	(-0.086, 0.047)	0.757
17	-0.018	(-0.099, 0.064)	0.844
16	-0.016	(-0.098, 0.066)	0.936
41	-0.016	(-0.116, 0.085)	0.409
30	-0.012	(-0.109, 0.085)	0.93
10	-0.011	(-0.068, 0.045)	0.706
12	-0.01	(-0.112, 0.091)	0.936
19	-0.01	(-0.073, 0.053)	0.946
1	-0.009	(-0.072, 0.054)	0.877
4	-0.006	(-0.068, 0.056)	0.996
3	0.005	(-0.053, 0.064)	0.994
9	0.009	(-0.051, 0.07)	0.951
43	0.014	(-0.073, 0.101)	0.84
14	0.015	(-0.041, 0.071)	0.516
20	0.019	(-0.045, 0.083)	0.767
35	0.021	(-0.116, 0.159)	0.966
7	0.024	(-0.04, 0.087)	0.522
25	0.025	(-0.028, 0.079)	0.308
8	0.026	(-0.013, 0.066)	0.064
28	0.041	(-0.045, 0.128)	0.325
38	0.05	(-0.069, 0.17)	0.484
24	**0.053**	**(-0.011, 0.116)**	**0.009**
5	**0.057**	**(-0.017, 0.131)**	**0.027**
44	**0.078**	**(-0.03, 0.187)**	**0.045**
6	**0.08**	**(0.019, 0.142)**	**0.0**
15	**0.112**	**(0.056, 0.169)**	**0.0**
11	**0.14**	**(0.069, 0.21)**	**0.0**

Supplementary Figure 10. Lung adenocarcinoma recurrence free survival Forest plot. Log hazard ratio of Cox proportional hazards model over the train sets of a 5-fold cross-validation on the NYU cohort. We averaged coefficients across fold and combined p-values with Fisher's combined probability test. This plot shows the relationship between HPCs and their positive or negative relevance in predicting recurrence. In order to find tissue patterns that can generalize across the cohort, we focused on HPCs that contain at least 10% of the total NYU number of patients and show statistical significance (p-value<0.05); these are displayed in bold. Source data are provided as a Source Data file.

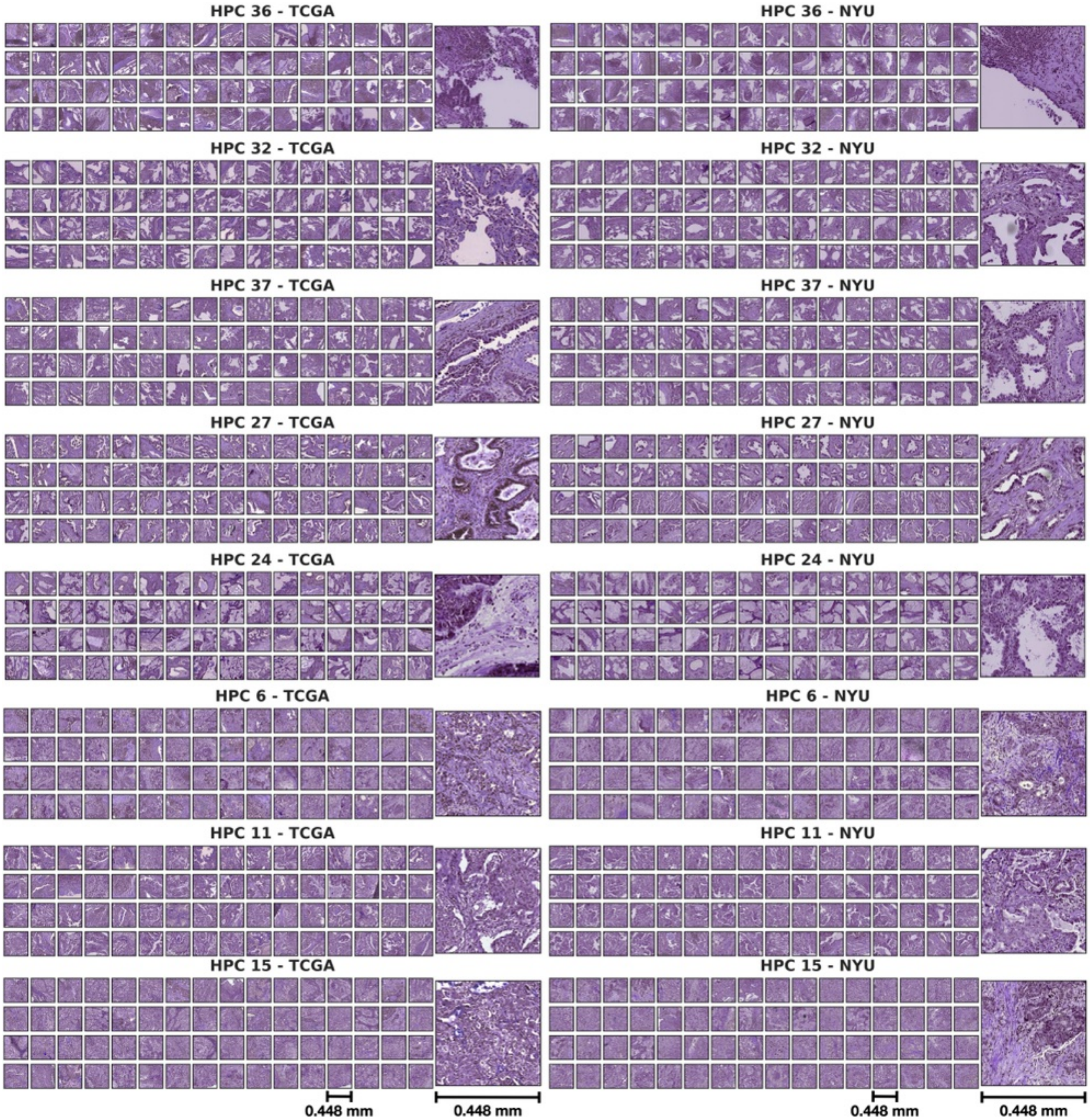

Supplementary Figure 11. The Cancer Genome Atlas (TCGA) and New York University NYU_1 tile samples for statistically significant HPCs on lung adenocarcinoma (LUAD) recurrence free survival. Source data are provided as a Source Data file.

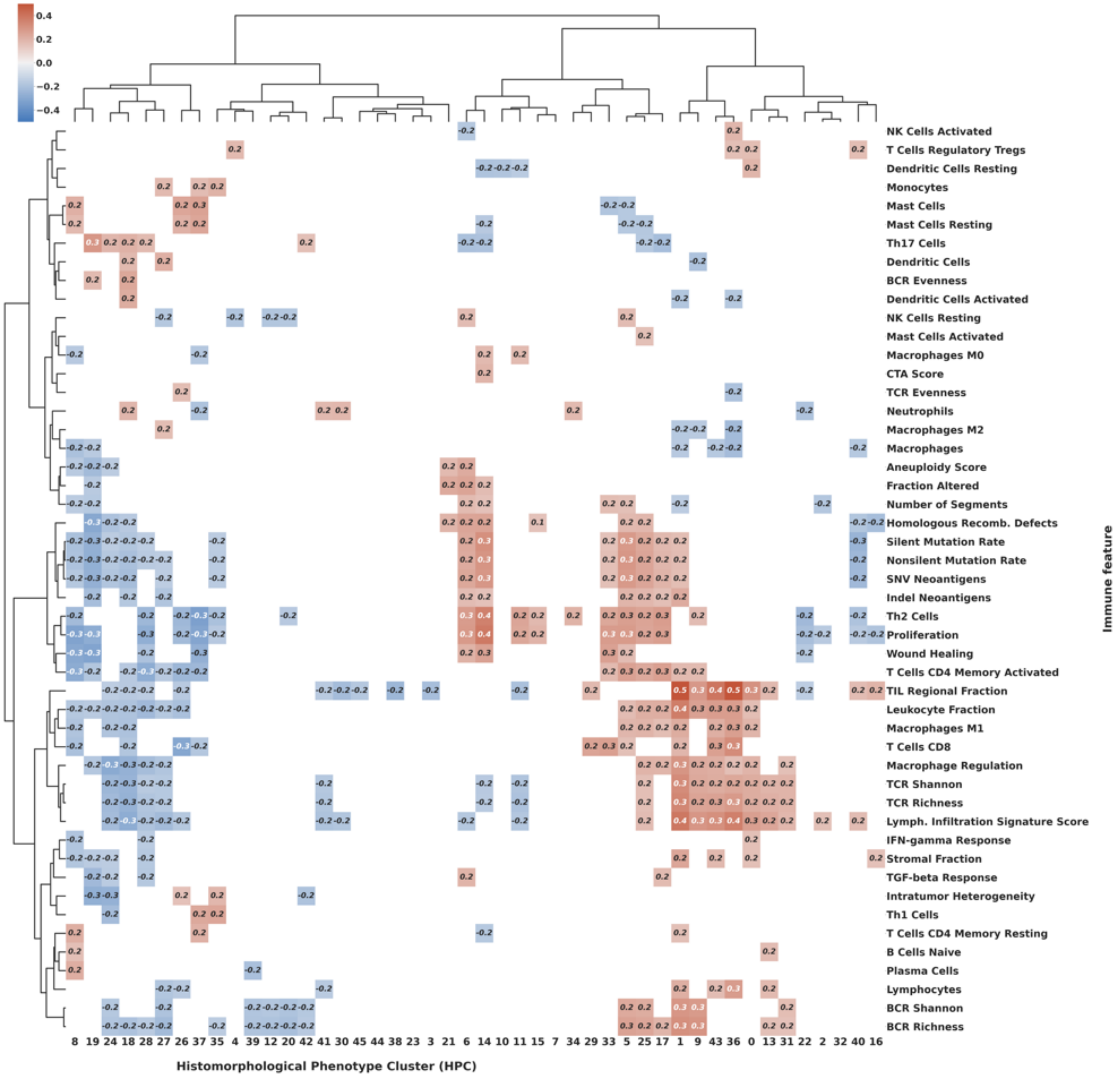

Supplementary Figure 12. Lung adenocarcinoma survival analysis and Histomorphological Phenotype Cluster (HPC) correlations. Bi-hierarchical clustering of HPCs and immune signature[3] spearman correlations. Source data are provided as a Source Data file.

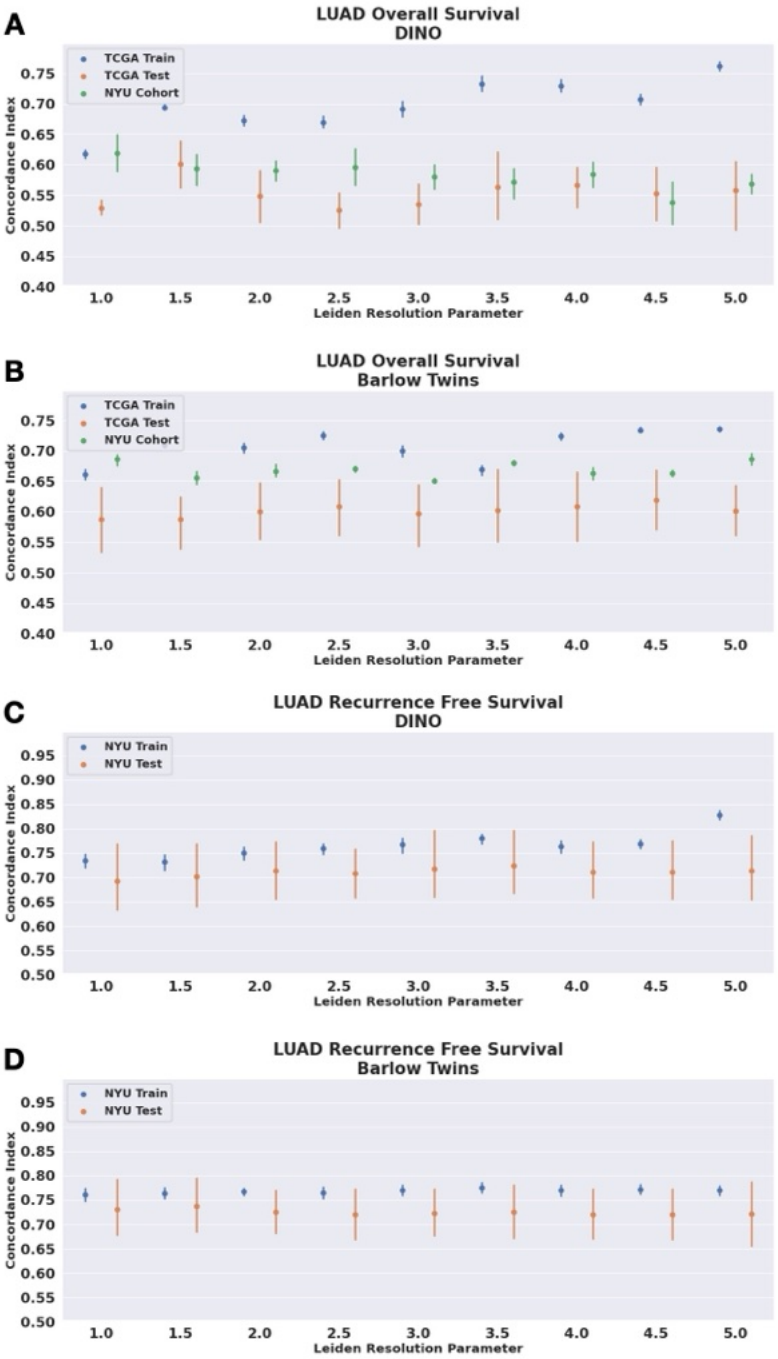

Supplementary Figure 13. LUAD survival analysis comparing Barlow Twins and DINO at different resolutions. **A** OS measured from HPL using DINO as self-supervised method. **B**OS measured from HPL using Barlow Twins as self-supervised method. **C** RFS measured from HPL using DINO as self-supervised method. **D** RFS measured from HPL using Barlow Twins as self-supervised method. DINO LUAD OS (Resolution 1.5) achieves a c-index of 0.612 with 95% confidence intervals (CI) of 0.569-0.643 in the TCGA Test set and a c-index of 0.597 with 95% CI of 0.565-0.625 in the NYU cohort. Barlow Twins (Resolution 2.0) achieves a TCGA Test set c-index of 0.600 with 95% CI of 0.551-0.649, and a NYU c-index of 0.666 with 95% CI of 0.656-0.676. DINO LUAD RFS (Resolution 1.5) achieves a comparable result to Barlow Twins with a c-index of 0.701 with 95% CI of 0.642-0.768. In comparison, Barlow Twins (Resolution 2.0) reaches a c-index of 0.725 with 95% CI of 0.681-0.770. The performance of both self-supervised methods is similar. However, DINO c-index fluctuates more with respect to the Leiden resolution parameter (resulting in more/less clusters) while Barlow Twins remains rather stable at different resolutions. All runs were done following a 5-fold cross-validation split similar to the one used in previous studies[1,2]. Source data are provided as a Source Data file.

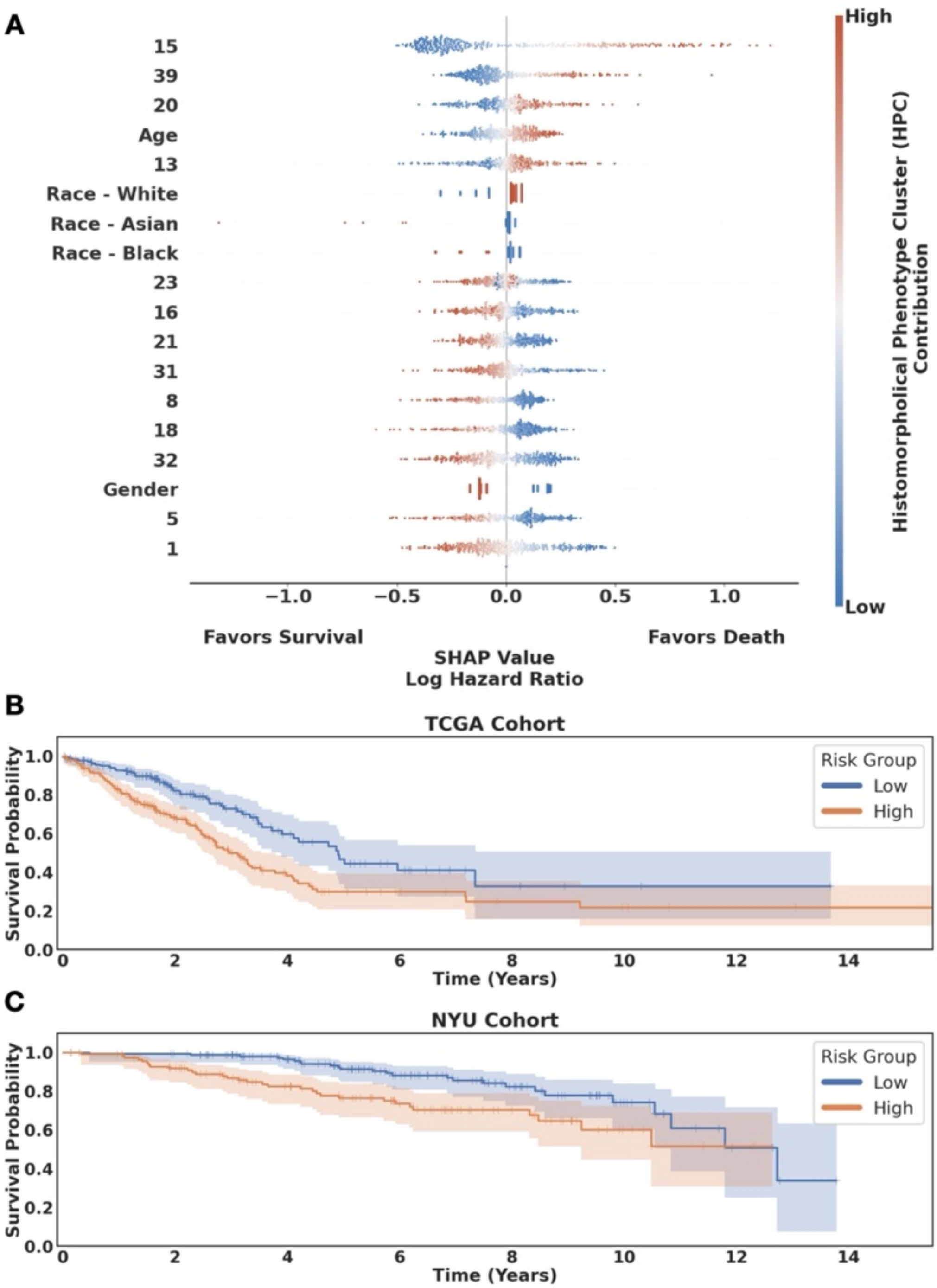

Supplementary Figure 14. LUAD Overall Survival analysis including patient metadata (age, sex, and race). Patient metadata is included as covariates in the Cox model along with HPC values. We evaluated the model using a 5-fold cross-validation over the TCGA data with a cohort from NYU as an additional independent set. **A**. SHAP (SHapley Additive exPlanations) plot showing how HPC (y-axis) weight on survival outcome (all race and ethnicities for TCGA data were submitted by the Tissue Source Sites that collected the sample and are presumed to be self-described/reported by the patient). **B.** High and low risk groups on TCGA cohort showing statistical significance (p-value $1.86 \times 10^{-4} < 0.05$) and c-index of 0.636 with 95% confidence intervals (CI) of $0.596 - 0.680$. **C.** High and low risk groups on NYU_1 cohort showing statistical significance (p-value $3.57 \times 10^{-3} < 0.05$) and c-index of 0.650 with 95% CI of $0.636 - 0.664$. Source data are provided as a Source Data file.

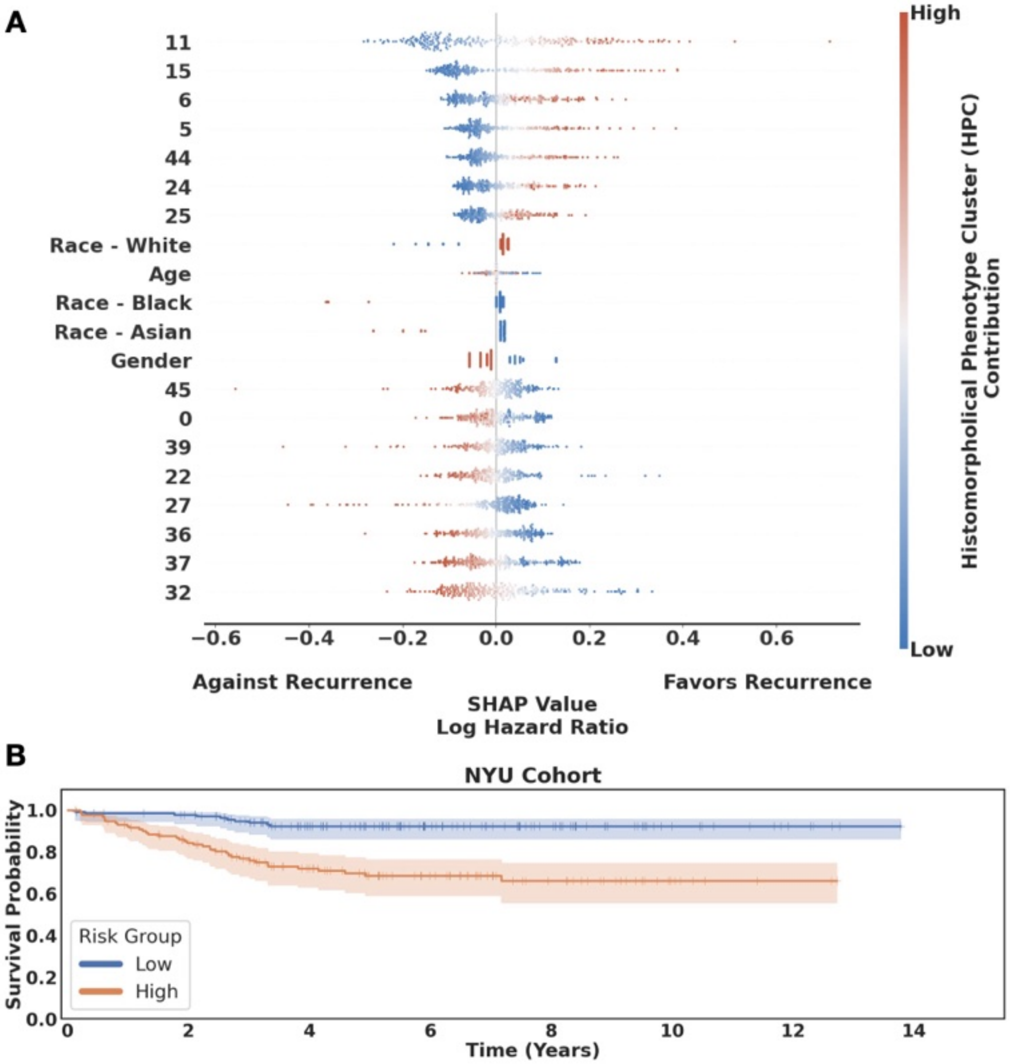

Supplementary Figure 15. LUAD Recurrence Free Survival analysis including patient metadata (age, sex, and race). Patient metadata is included as covariates in the Cox model along with HPC values. We evaluated the model using a 5-fold cross-validation over the NYU cohort (race was self-described by patients). **A**. SHAP (SHapley Additive exPlanations) plot showing how HPC (y-axis) weight on survival outcome. **B.** High and low risk groups on NYU cohort showing statistical significance (p-value $8.82 \times 10^{-7} < 0.05$) and c-index of 0.748 with 95% confidence intervals (CI) of $0.698 - 0.808$. Source data are provided as a Source Data file.

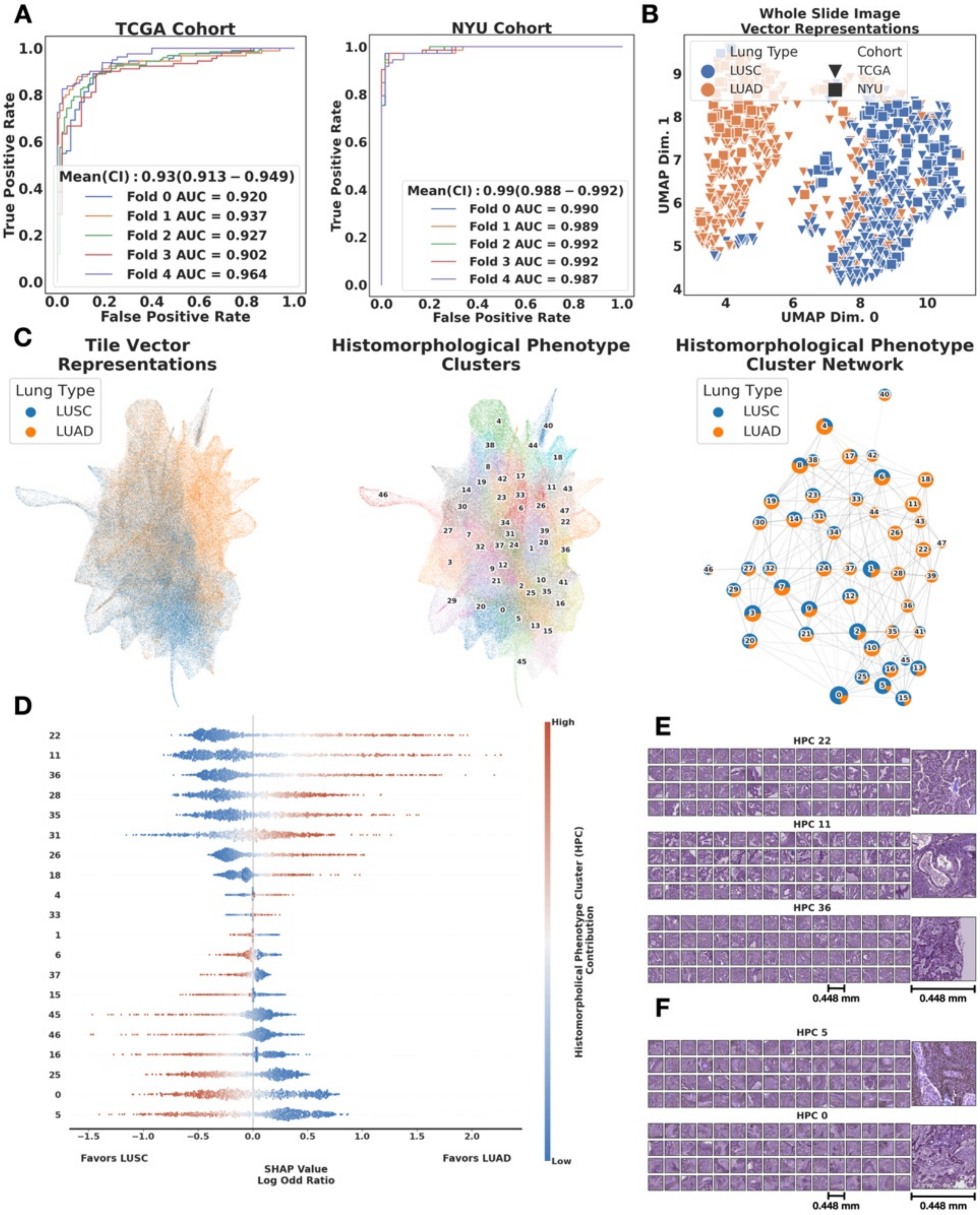
A
TCGA Cohort
True Positive Rate
False Positive Rate
Mean(CI) : 0.93(0.913 – 0.949)
Fold 0 AUC = 0.920
Fold 1 AUC = 0.937
Fold 2 AUC = 0.927
Fold 3 AUC = 0.902
Fold 4 AUC = 0.964
NYU Cohort
Mean(CI) : 0.99(0.988 – 0.992)
Fold 0 AUC = 0.990
Fold 1 AUC = 0.989
Fold 2 AUC = 0.992
Fold 3 AUC = 0.992
Fold 4 AUC = 0.987
B
Whole Slide Image Vector Representations
Lung Type
LUSC
LUAD
Cohort
TCGA
NYU
UMAP Dim. 1
UMAP Dim. 0
C
Tile Vector Representations
Histomorphological Phenotype Clusters
Histomorphological Phenotype Cluster Network
D
Favors LUSC
SHAP Value
Log Odd Ratio
Favors LUAD
High
Low
Histomorphological Phenotype Cluster (HPC) Contribution
E
HPC 22
HPC 11
HPC 36
0.448 mm
F
HPC 5
HPC 0
0.448 mm

Supplementary Figure 16. Lung adenocarcinoma (LUAD) versus squamous cell carcinoma (LUSC) classification performance and representation analysis. A. Receiver operating characteristic (ROC) curve over a 5-fold cross-validation on TCGA dataset and NYU_2 dataset as an independent cohort, with mean AUCs of 0.93 and 0.99 respectively. **B.** Uniform Manifold Approximation and Projection (UMAP) dimensionality reduction of whole slide image (WSI) compositional vector representations for TCGA and NYU_2 cohorts. Each representation is labeled with the corresponding lung type, LUSC (blue) and LUAD (orange). **C.** UMAP dimensionality reduction of tile vector representations, each tile label corresponds to the associated WSI lung type (left) and HPC membership (middle), and partition-based graph abstraction (PAGA) of HPC and their connections (right). **D.** SHAP (SHapley Additive exPlanations) plot. **E.** Examples of tiles randomly selected from the 3 top HPCs enriched in LUAD-specific phenotypes according to SHAP analysis from panel D. **F.** Examples of tiles randomly selected from the 3 top HPCs enriched in LUSC-specific phenotypes according to SHAP analysis from panel D. Source data are provided as a Source Data file.

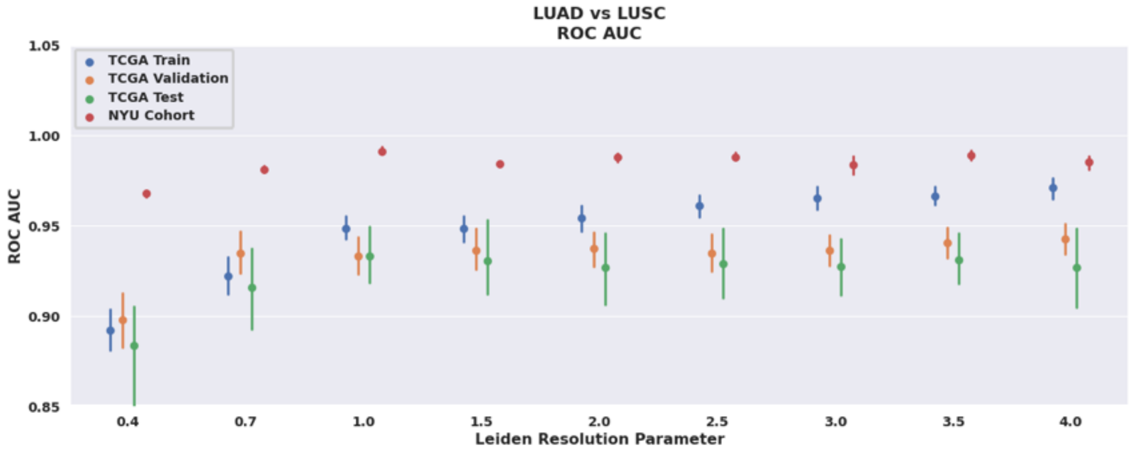

Supplementary Figure 17. Effect of the Leiden resolution on the LUAD/LUSC classification task. This run was done on a 5 fold cross-validation. Source data are provided as a Source Data file.

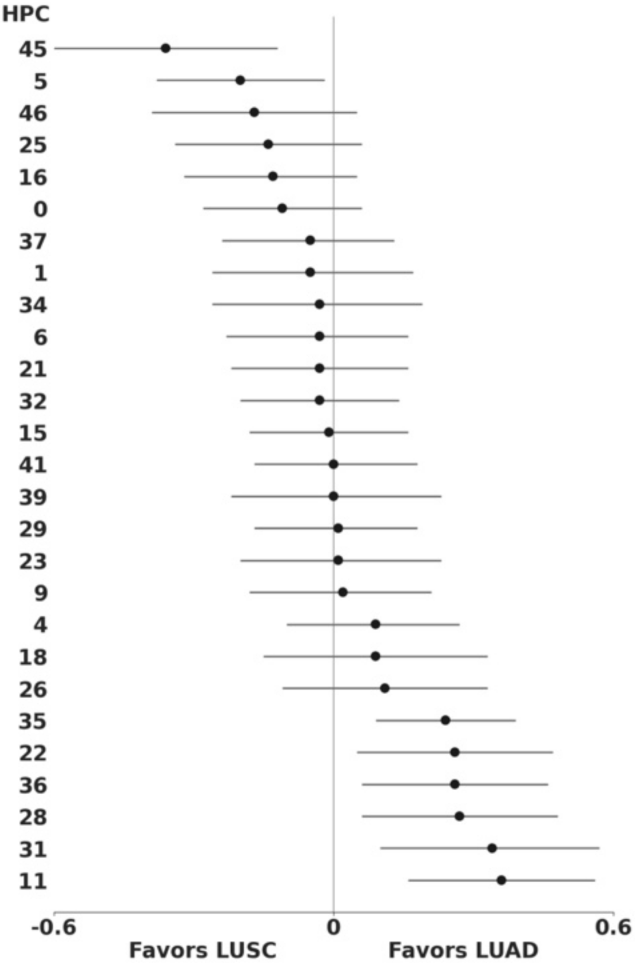

HPC	Log Odds Ratio	95% CI	P-Value	Predominant Lung Type	Purity %
45	-0.36	(-0.6, -0.12)	0.003	LUSC	97
5	-0.2	(-0.38, -0.02)	0.028	LUSC	87
46	-0.17	(-0.39, 0.05)	0.129	LUSC	77
25	-0.14	(-0.34, 0.06)	0.166	LUSC	78
16	-0.13	(-0.32, 0.05)	0.148	LUSC	63
0	-0.11	(-0.28, 0.06)	0.2	LUSC	79
37	-0.05	(-0.24, 0.13)	0.573	LUAD	73
1	-0.05	(-0.26, 0.17)	0.654	LUSC	73
34	-0.03	(-0.26, 0.19)	0.773	LUSC	56
6	-0.03	(-0.23, 0.16)	0.745	LUAD	50
21	-0.03	(-0.22, 0.16)	0.758	LUSC	58
32	-0.03	(-0.2, 0.14)	0.751	LUSC	63
15	-0.01	(-0.18, 0.16)	0.908	LUSC	77
41	0.0	(-0.17, 0.18)	0.975	LUAD	71
39	0.0	(-0.22, 0.23)	0.971	LUAD	81
29	0.01	(-0.17, 0.18)	0.942	LUSC	68
23	0.01	(-0.2, 0.23)	0.898	LUSC	50
9	0.02	(-0.18, 0.21)	0.866	LUSC	62
4	0.09	(-0.1, 0.27)	0.378	LUAD	71
18	0.09	(-0.15, 0.33)	0.455	LUAD	97
26	0.11	(-0.11, 0.33)	0.324	LUAD	95
35	0.24	(0.09, 0.39)	0.002	LUAD	74
22	0.26	(0.05, 0.47)	0.014	LUAD	96
36	0.26	(0.06, 0.46)	0.009	LUAD	86
28	0.27	(0.06, 0.48)	0.013	LUAD	89
31	0.34	(0.1, 0.57)	0.006	LUSC	53
11	0.36	(0.16, 0.56)	0.0	LUAD	97

Supplementary Figure 18. Lung adenocarcinoma (LUAD) versus squamous cell carcinoma (LUSC) Forest plot. Forest plot of the logistic regression's coefficients over the train sets of a 5-fold cross-validation. We averaged coefficients across fold and combined p-values with Fisher's combined probability test. We highlighted statistically significant HPCs (p-value$<$ 0.05) and included each HPC's dominant type and purity. We calculated purity as the percentage of the dominant cancer type tiles with respect to the total HPC tiles. Source data are provided as a Source Data file.

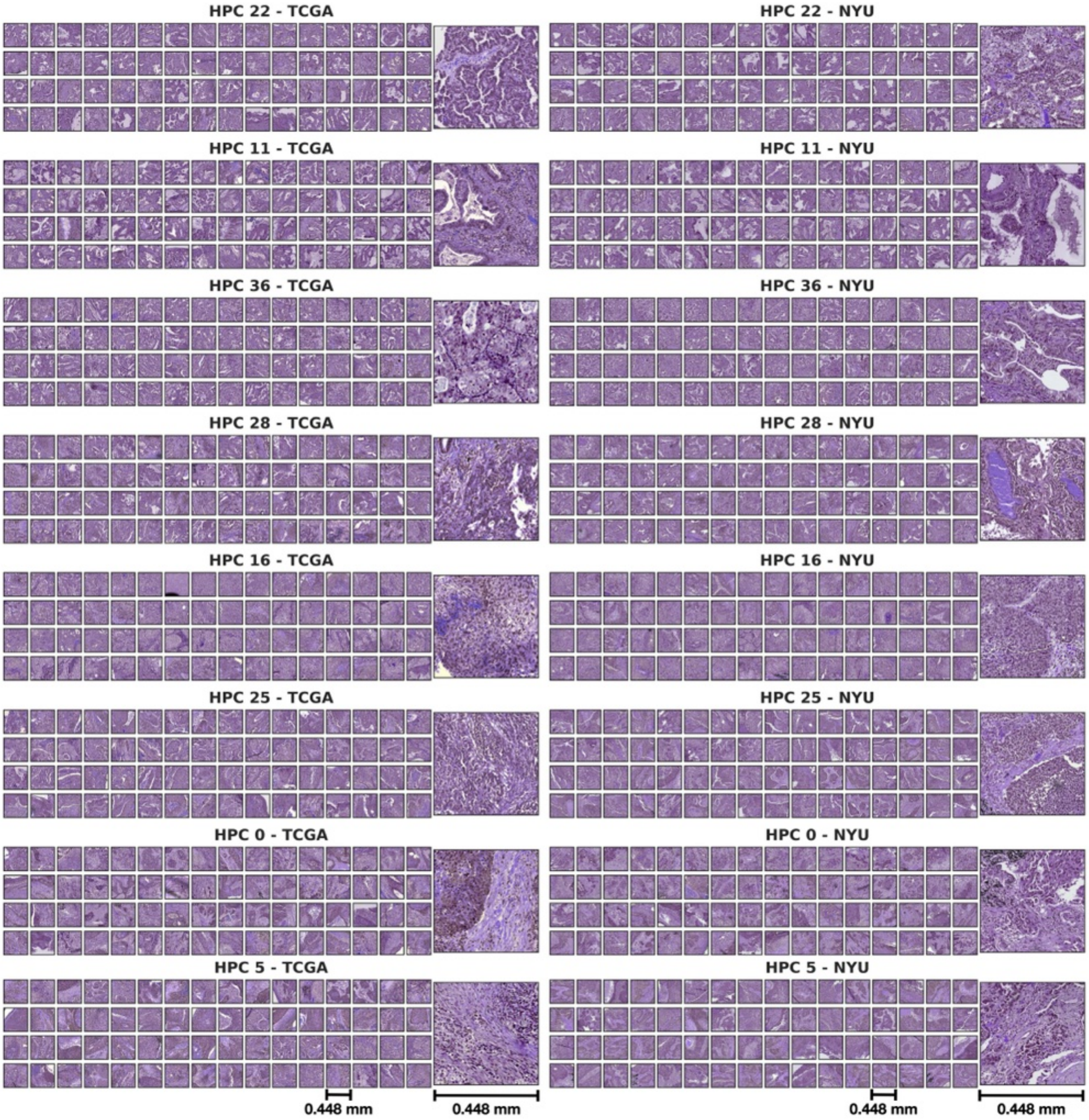

Supplementary Figure 19. The Cancer Genome Atlas (TCGA) and New York University NYU_2 tile samples for statistically significant HPCs on lung adenocarcinoma (LUAD) versus cell squamous cell carcinoma (LUSC) classification. Source data are provided as a Source Data file.

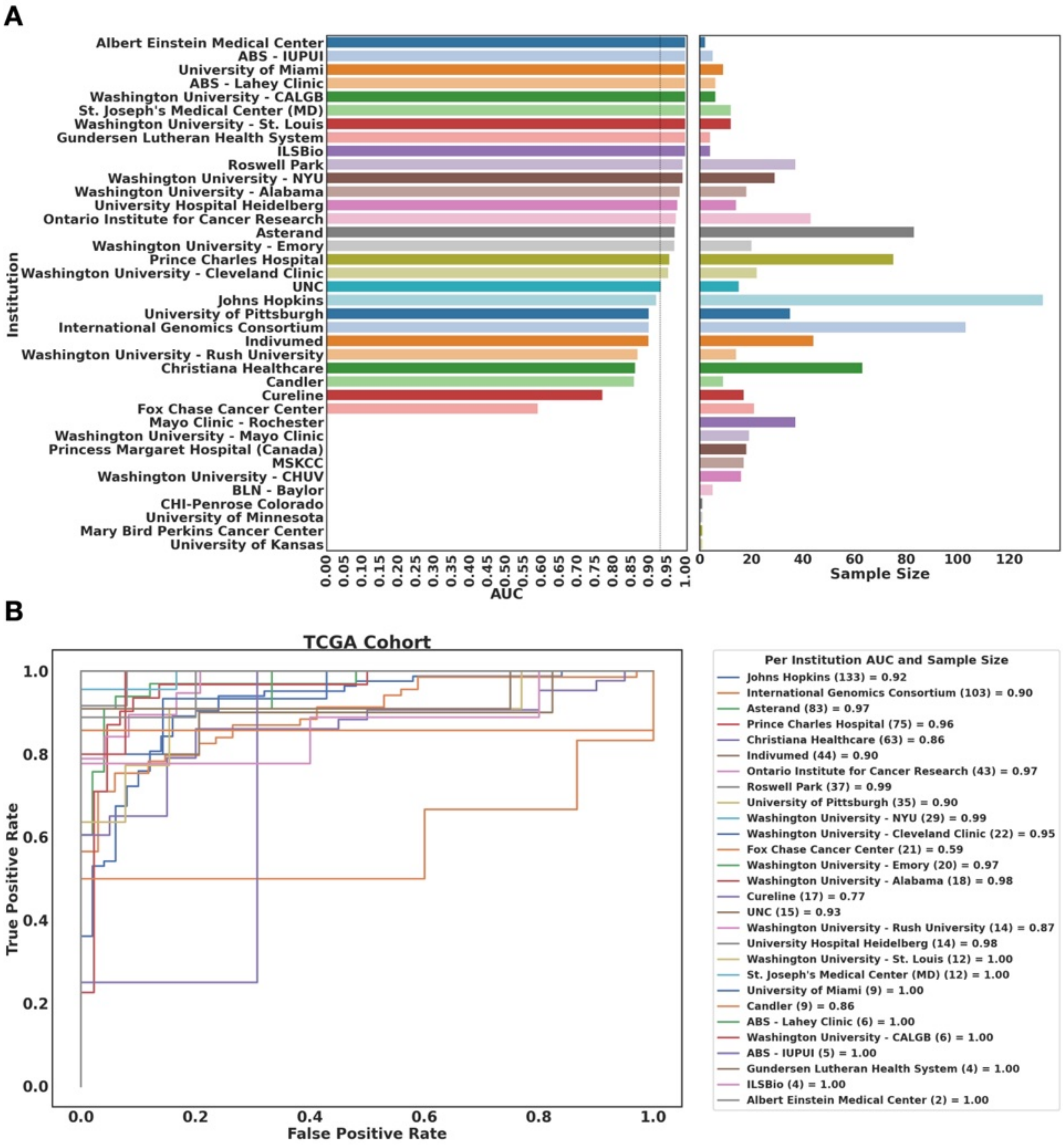

Supplementary Figure 20. Lung adenocarcinoma (LUAD) versus squamous cell carcinoma (LUSC) classification performance per institution. A Receiver operating characteristic (ROC) area under the curve (AUC) per TCGA institution and WSI sample size. Institutions without an AUC value only have one kind of lung type, not allowing to compute the ROC AUC. **B** ROC curve per TCGA institution. These results correspond to the 5-fold cross-validation on TCGA dataset and NYU dataset as independent cohort as included in **Figure 13 A**. Source data are provided as a Source Data file.

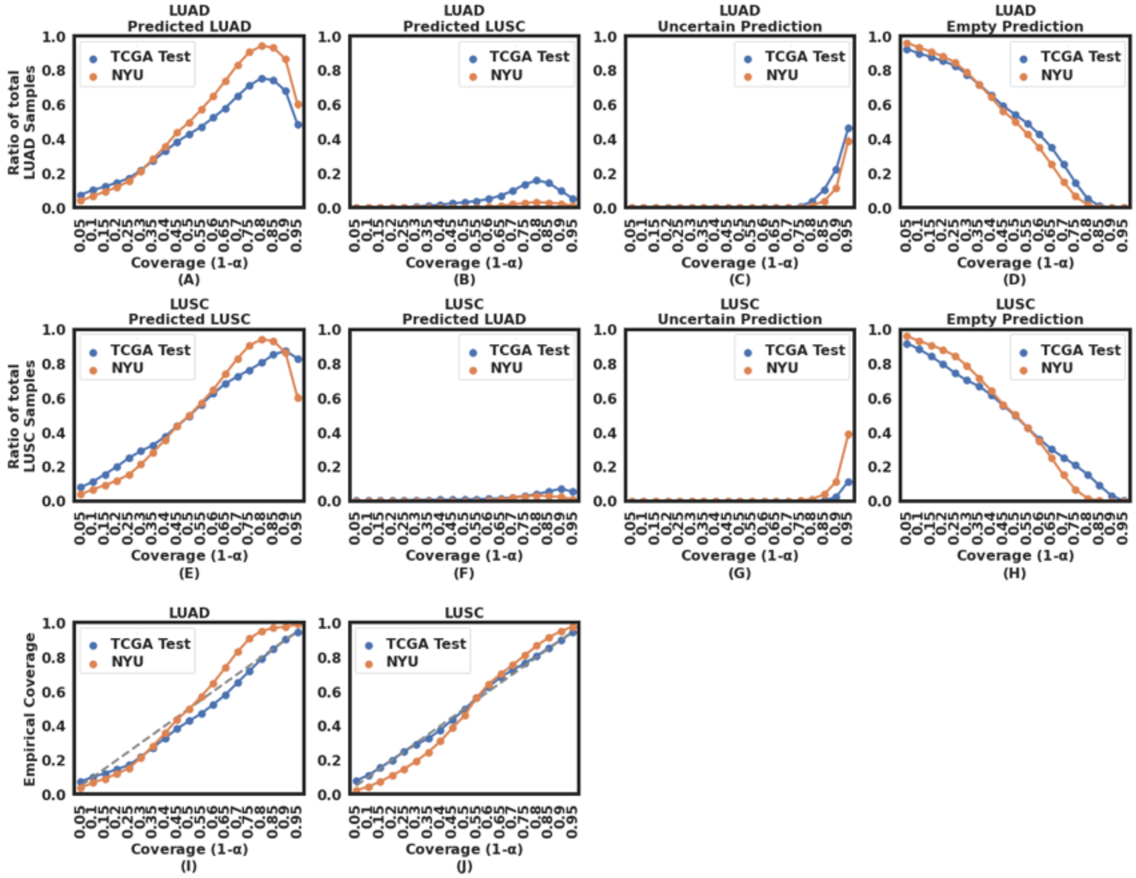

Supplementary Figure 21. Label-Conditional Conformal Prediction on on the LUAD vs LUSC prediction task. We performed MICP[4] for a range of coverage $(1-\alpha)$ from 0.05 to 0.95. The figure above shows for LUAD and LUSC the ratios of correct single predictions (A,F), incorrect single predictions (B,G), uncertain predictions (C,H), and empty predictions (D,I). Uncertain predictions are defined as prediction sets that contain both labels and empty predictions are as prediction sets that contain no label. Finally, we include the empirical coverage for LUAD (I) and LUSC (J), highlighting the calibration coverage in the gray dashed line. We define non-conformity measure as one minus the probability of the correct class. For each coverage measure $(1-\alpha)$, we obtained 1000 samples by running 200 trials per fold, out of the initial folds used in the 5-fold cross-validation LUAD vs LUSC task. For each trial, we randomly subsampled 50% of the total samples on the TCGA validation set (calibration set), TCGA test set (quantification set), and NYU cohort (additional quantification set), resulting into approximately 100 samples for the TCGA validation and test set, and 70 samples for the NYU cohort. We can see higher uncertainty in TCGA LUAD samples compared to TCGA LUSC samples. The model is also more sensitive to misclassifications in LUAD, barely making any in LUSC. Indeed, this result may be explained by LUAD solid samples, which are harder to differentiate from LUSC than other LUAD subtypes such as acinar, papillary, micropapillary, or lepidic. HPL displays similar confidence on LUAD and LUSC samples of the NYU cohort, with higher uncertainty in LUSC compared to TCGA. Nonetheless, HPL makes larger correct single predictions on both subtypes in the NYU cohort than in TCGA. Possibly due to NYU WSIs being cleaner and less noisy samples than the TCGA counterparts, in particular on the LUAD cases. Source data are provided as a Source Data file.

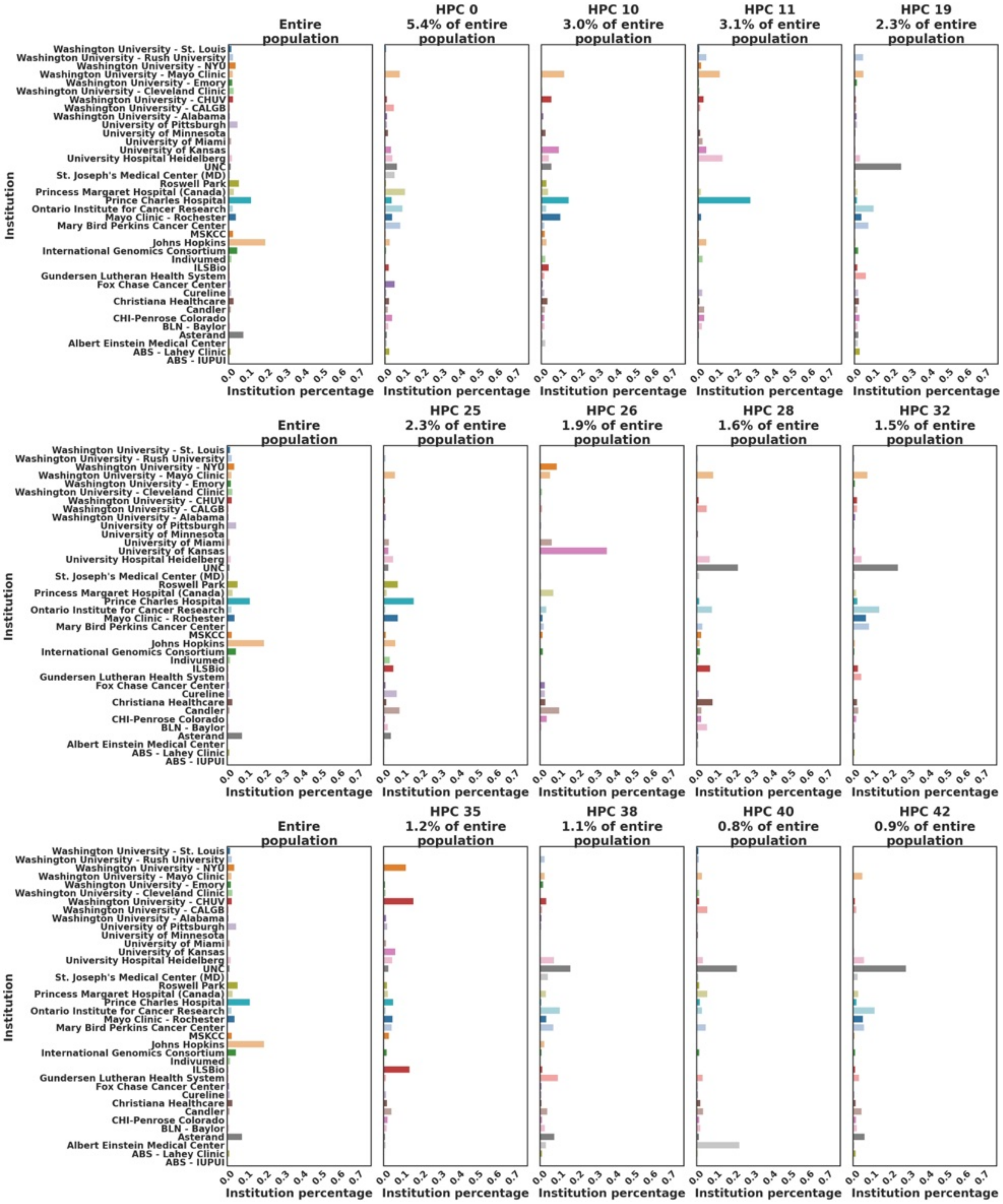

Supplementary Figure 22. Lung adenocarcinoma (LUAD) versus squamous cell carcinoma (LUSC) HPCs: institution distribution per HPC and for the entire population of tissue tiles. we show 12 randomly sampled HPCs. Source data are provided as a Source Data file.

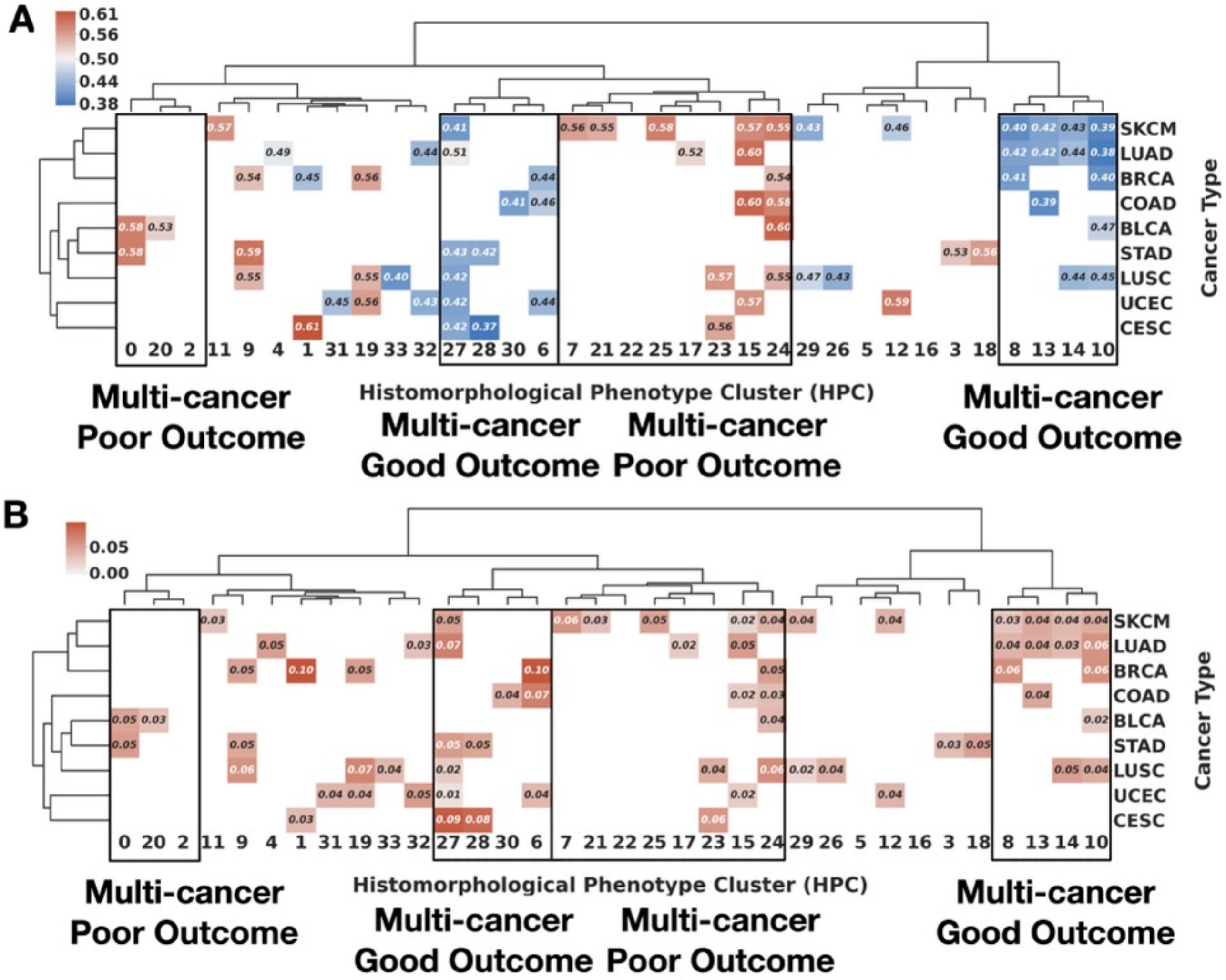

Supplementary Figure 23. Concordance index between multi-cancer HPCs and overall survival. A Mean C-index values across the 5-fold cross validation. Values below 0.5 (blue) indicate that higher percentage of the HPC favors longer survival (good outcome), while those above 0.5 (red) indicate that higher percentage the HPC favors shorter survival (poor outcome). **B** Variation for the 95% confidence interval over the 5-fold cross validation. We only display statistically significant values of the log rank test for the high and low risk patient split (*p-value* < 0.05). Risk groups are defnied by taking the median risk in the training set (in this casem the HPC value). Source data are provided as a Source Data file.

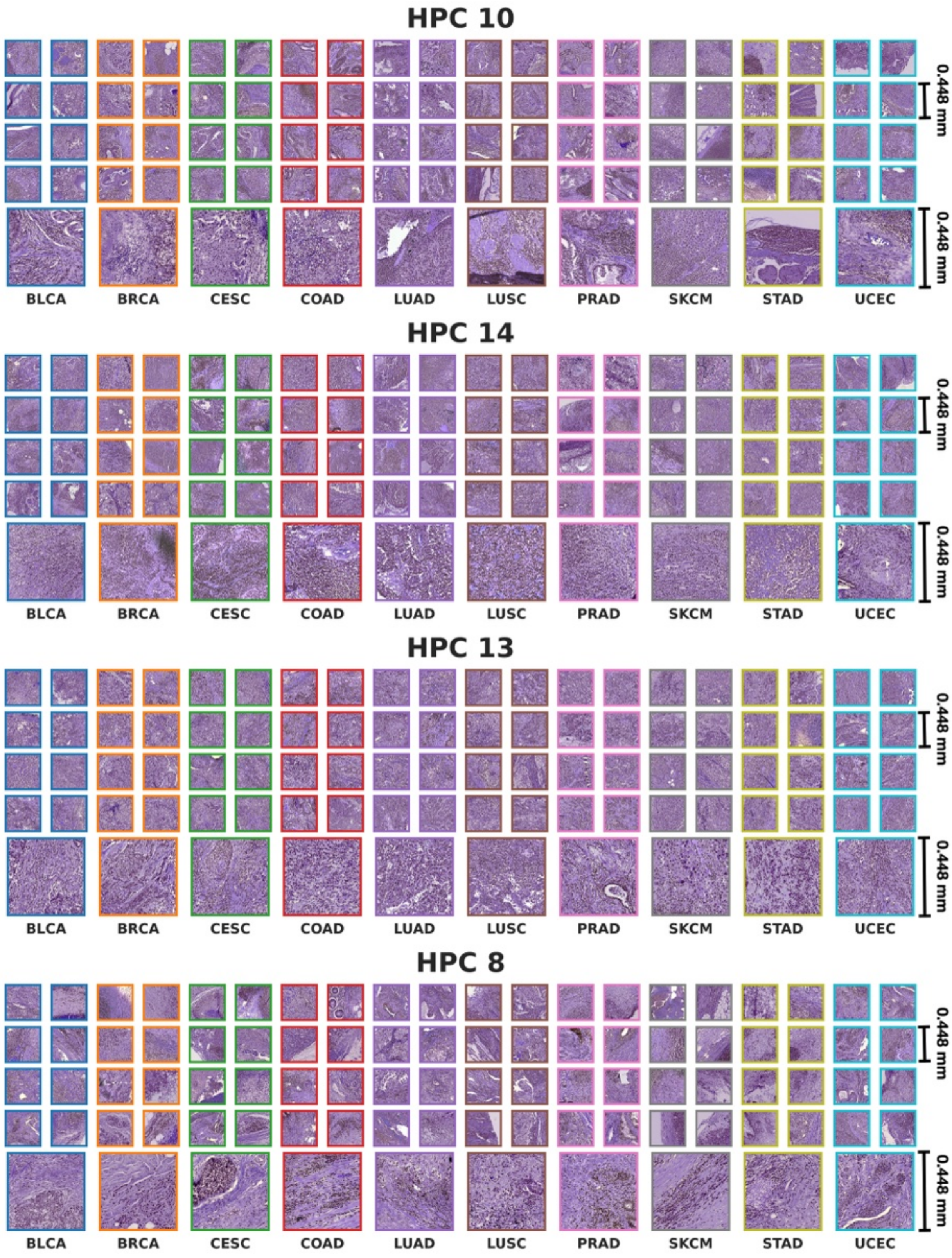

Supplementary Figure 24. Examples of tiles from The Cancer Genome Atlas (TCGA) assigned to HPCs associated with good outcomes, and corresponding to the right-hand side of the bi-herachical clustering in **Figure 9A**. Source data are provided as a Source Data file.

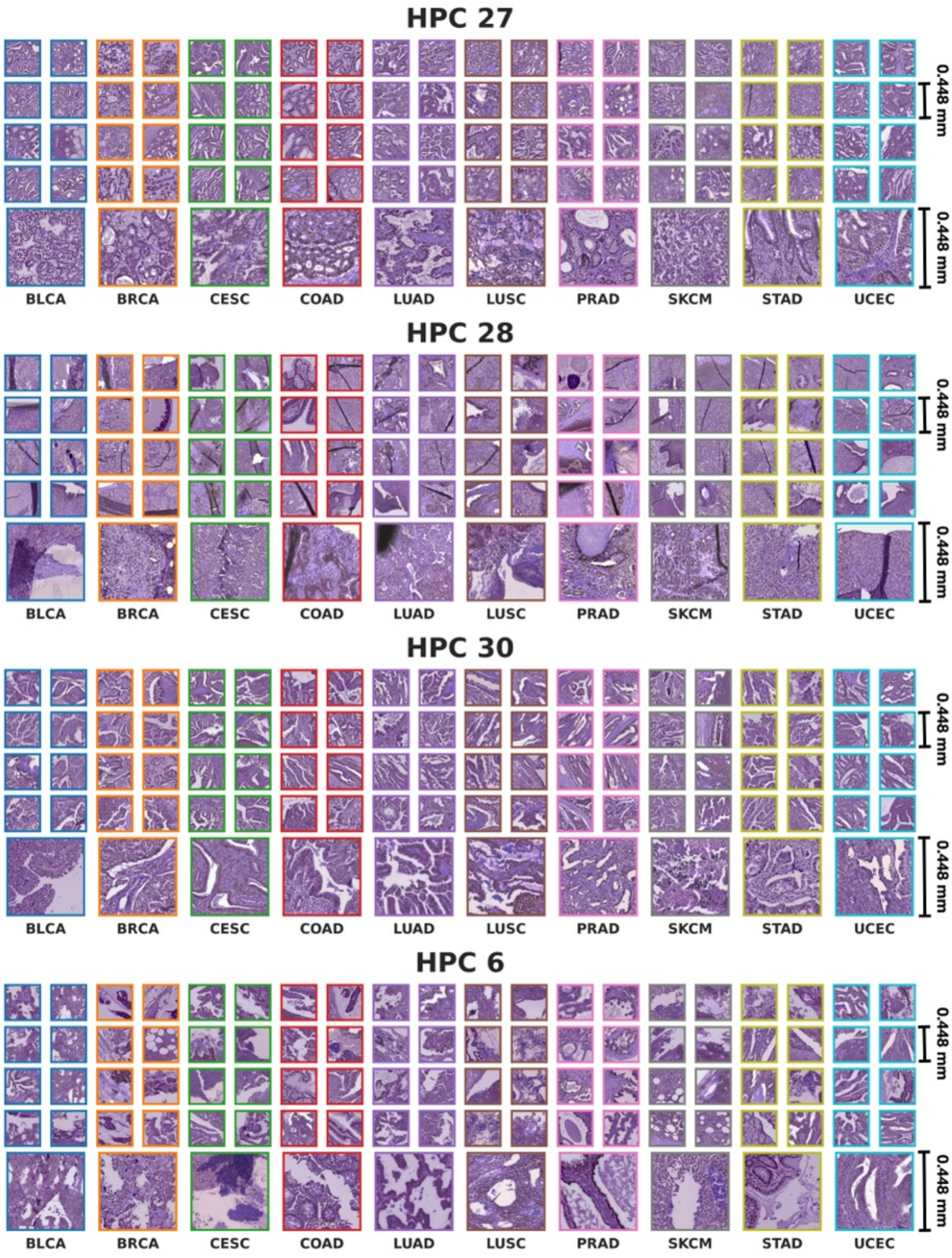

Supplementary Figure 25. Examples of tiles from The Cancer Genome Atlas (TCGA) assigned to HPCs associated with good outcomes, and corresponding to the left-hand side of the bi-herachical clustering in **Figure 9A**. Source data are provided as a Source Data file.

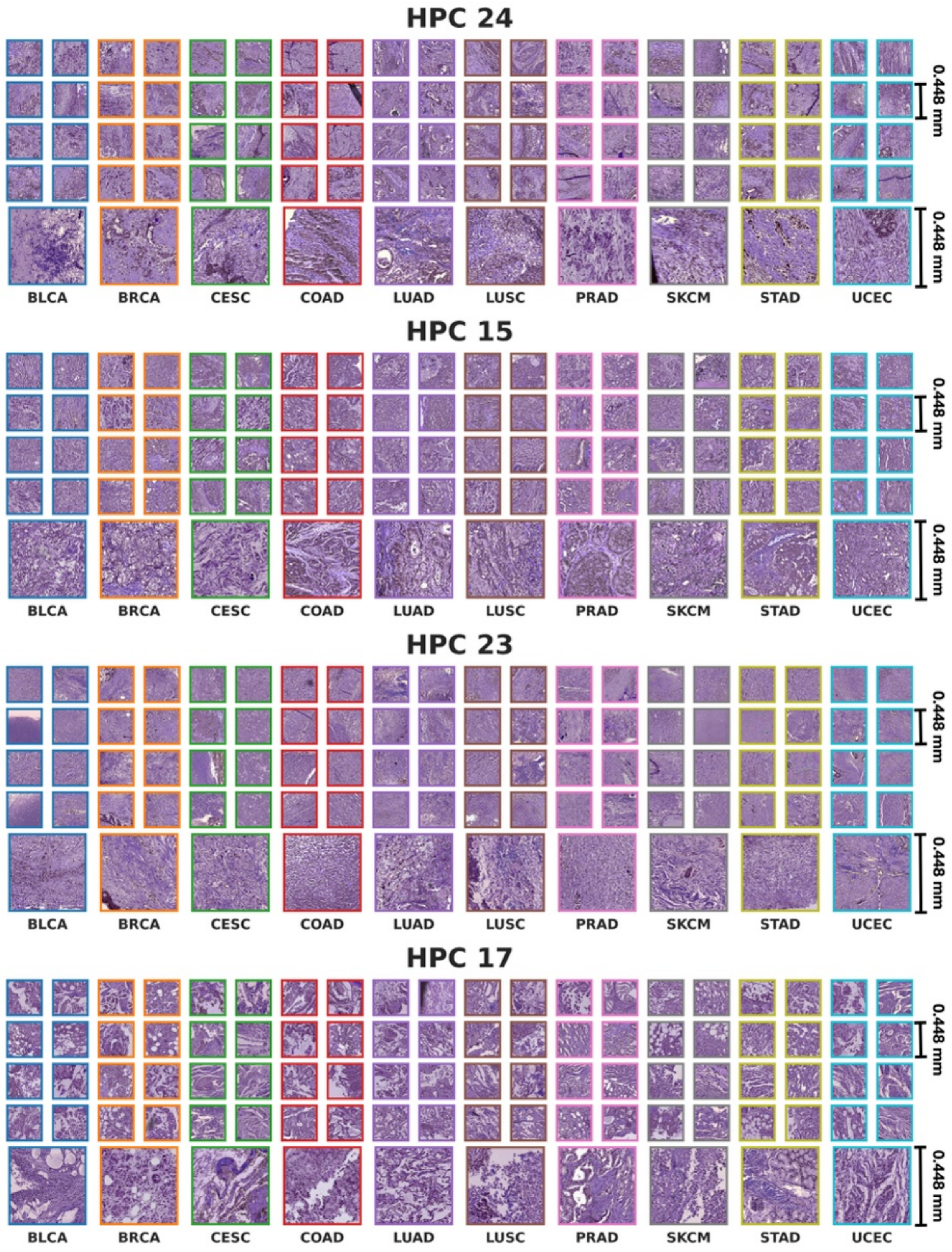

Supplementary Figure 26. Examples of tiles from The Cancer Genome Atlas (TCGA) assigned to HPCs associated with poor outcomes, and corresponding to the right-hand side of the bi-herachical clustering in **Figure 9A**. Source data are provided as a Source Data file.

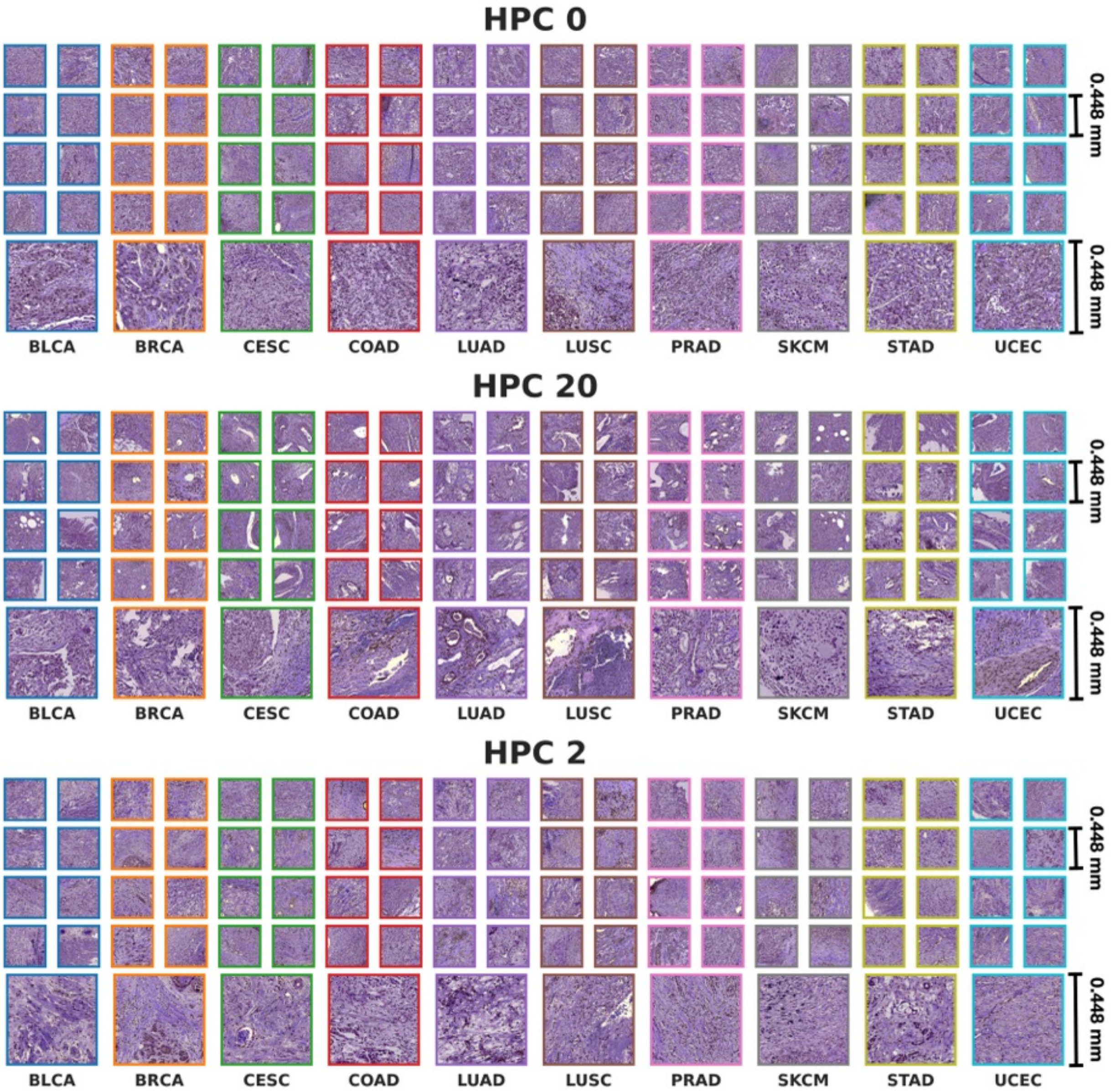

Supplementary Figure 27. Examples of tiles from The Cancer Genome Atlas (TCGA) assigned to HPCs associated with poor outcomes, and corresponding to the left-hand side of the bi-hierarchical clustering in **Figure 9A**. Source data are provided as a Source Data file.

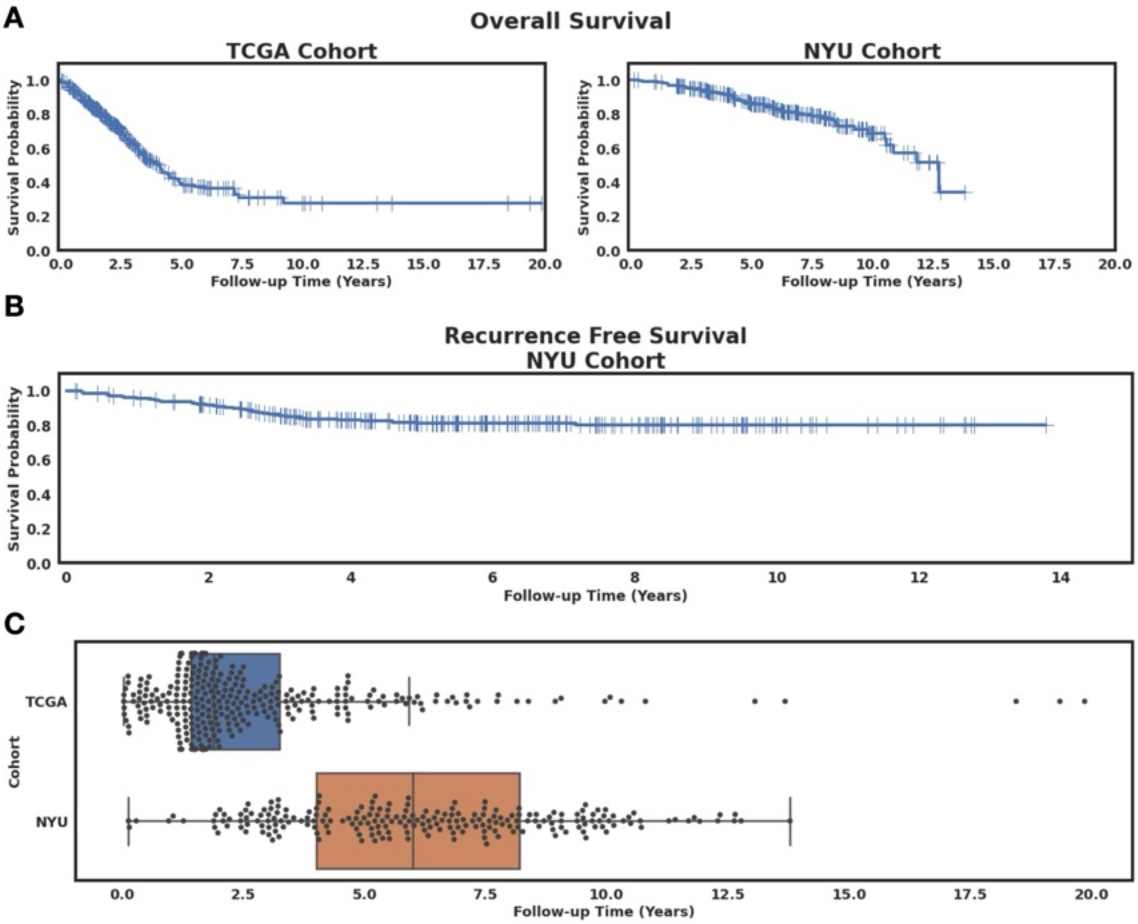

Supplementary Figure 28. Dataset description for lung adenocarcinoma overall and recurrence free survival. **A** Kaplan-Meier curves of overall survival events of lung adenocarcinoma (LUAD) of The Cancer Genome Atlas (TCGA) and New York University NYU_1 cohorts. Censored samples are represented by a cross. **B** Kaplan-Meier curves of recurrence free survival events of LUAD systemic and loco-regional recurrence for the NYU_1 cohort. The NYU cohort is composed by three types: systemic, loco-regional, and new primary. We censored new primary cases as we focus on systemic and loco-regional. Censored samples are represented by a cross. **C** Follow-up times from patients of TCGA and NYU_1 cohorts. NYU patients are considerably larger follow-up times allowing a more refined study of recurrence. Source data are provided as a Source Data file.

Encoder Network $E : x \to z$
$x \in \mathbb{R}^{224\times 224\times 3}$
Conv2D Layer, 7x7, stride 2, pad downscale, Batch Norm, and ReLU<br>$224 \times 224 \times 3 \to 112 \times 112 \times 32$
ResNet Conv2D Layer, 5x5, stride 1, pad same, Batch Norm, and ReLU<br>$112 \times 112 \times 32 \to 112 \times 112 \times 32$
ResNet Conv2D Layer, 5x5, stride 1, pad same, Batch Norm, and ReLU<br>$112 \times 112 \times 32 \to 112 \times 112 \times 32$
Conv2D Layer, 4x4, stride 2, pad downscale, Batch Norm, and ReLU<br>$112 \times 112 \times 32 \to 56 \times 56 \times 32$
ResNet Conv2D Layer, 5x5, stride 1, pad same, Batch Norm, and ReLU<br>$56 \times 56 \times 32 \to 56 \times 56 \times 32$
Attention Layer at $56 \times 56 \times 32$
ResNet Conv2D Layer, 5x5, stride 1, pad same, Batch Norm, and ReLU<br>$56 \times 56 \times 32 \to 28 \times 28 \times 64$
ResNet Conv2D Layer, 5x5, stride 1, pad same, Batch Norm, and ReLU<br>$28 \times 28 \times 64 \to 28 \times 28 \times 64$
ResNet Conv2D Layer, 5x5, stride 1, pad same, Batch Norm, and ReLU<br>$28 \times 28 \times 64 \to 28 \times 28 \times 64$
Conv2D Layer, 4x4, stride 2, pad downscale, Batch Norm, and ReLU<br>$28 \times 28 \times 64 \to 14 \times 14 \times 128$
ResNet Conv2D Layer, 5x5, stride 1, pad same, Batch Norm, and ReLU<br>$14 \times 14 \times 128 \to 14 \times 14 \times 128$
ResNet Conv2D Layer, 5x5, stride 1, pad same, Batch Norm, and ReLU<br>$14 \times 14 \times 128 \to 14 \times 14 \times 128$
Conv2D Layer, 4x4, stride 2, pad downscale, Batch Norm, and ReLU<br>$14 \times 14 \times 128 \to 7 \times 7 \times 256$
ResNet Conv2D Layer, 5x5, stride 1, pad same, Batch Norm, and ReLU<br>$7 \times 7 \times 256 \to 7 \times 7 \times 256$
ResNet Conv2D Layer, 5x5, stride 1, pad same, Batch Norm, and ReLU<br>$7 \times 7 \times 256 \to 7 \times 7 \times 256$
Conv2D Layer, 4x4, stride 2, pad downscale, Batch Norm, and ReLU<br>$7 \times 7 \times 256 \to 4 \times 4 \times 512$
Flatten $4 \times 4 \times 512 \to 1024$
Dense Layer, Batch Norm and ReLU, $1024 \to 1024$
Dense Layer, Batch Norm and ReLU, $1024 \to 1024$
Dense Layer, Batch Norm and ReLU, $1024 \to 128$

Supplementary Figure 29. Detailed description of the CNN layers. Source data are provided as a Source Data file.

A

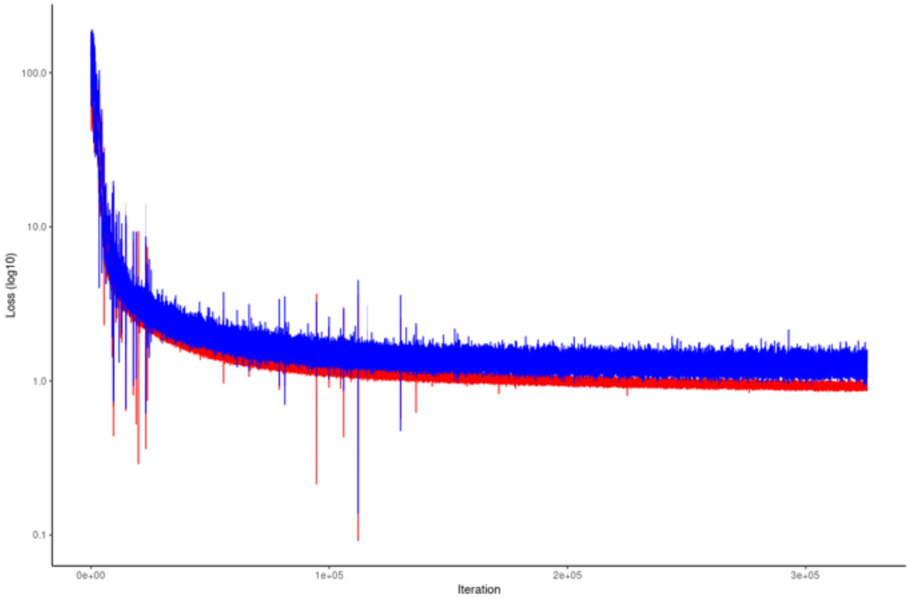

B

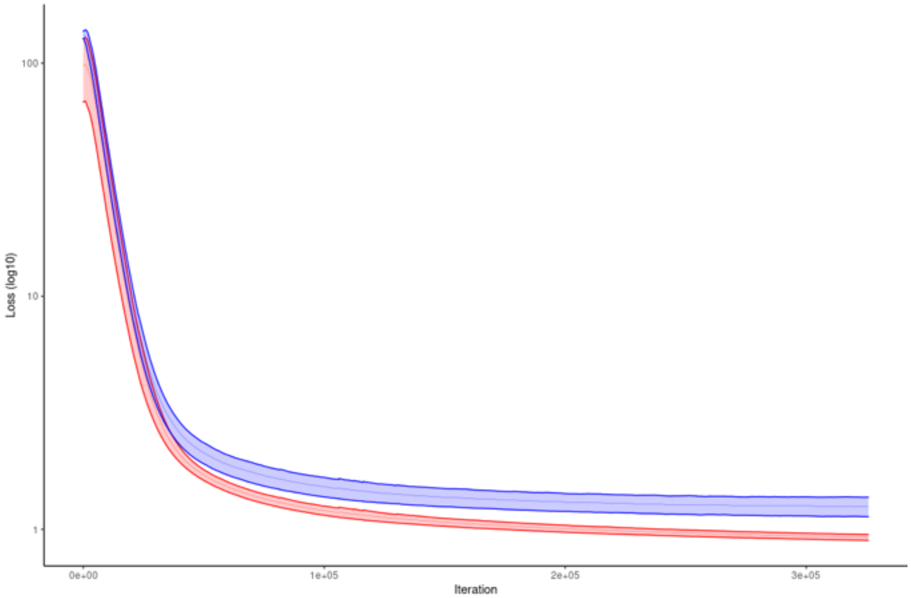

Supplementary Figure 30. Loss during training of Barlow-Twins-based network. A Raw losses for the train (red) and validation (blue) sets. **B** Same as panel A but after smoothing the curve using exponential moving average of the batch losses over 1 epoch for clearer display. Error shows the standard deviation from the 5-fold cross-validation runs. Source data are provided as a Source Data file.

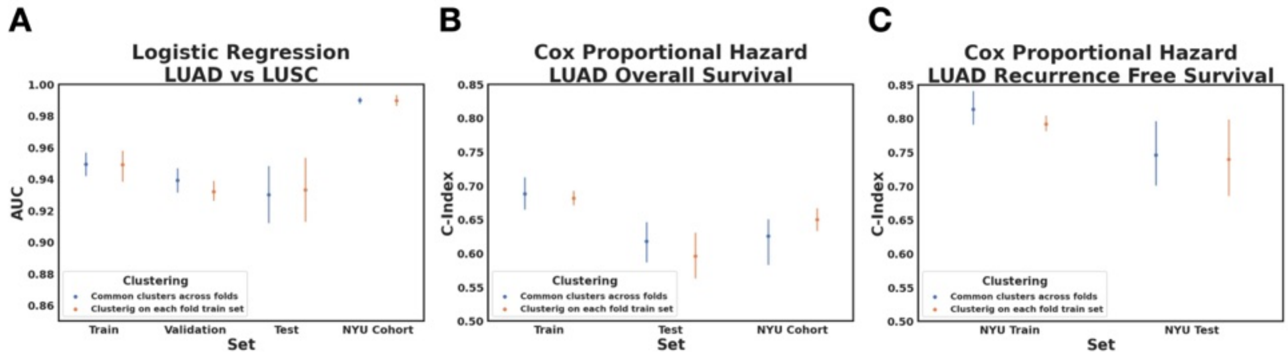

Supplementary Figure 31. Clustering impact on performance. Performance variation when clustering is done on each fold's train set compared to when a particular cluster configuration is hold the same across folds for the classification or survival task. **A** Performance variation on logistic regression for LUSC versus LUAD classification. **B** Performance variation on Cox proportional hazard for LUAD overall survival. **C** Performance variation on Cox proportional hazard for LUAD recurrence free survival. Source data are provided as a Source Data file.

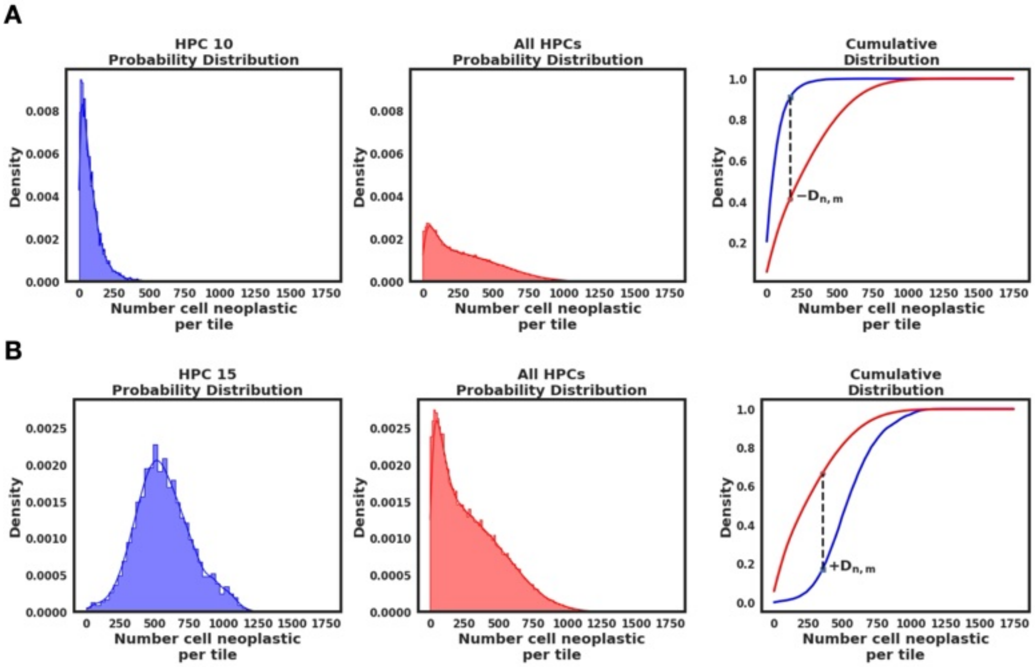

Supplementary Figure 32. Kolmogorov-Smirnov test for lung adenocarcinoma cell type over and under-representation. Representations of usage of Two-sample Kolmogorov-Smirnov (K-S) test to account for over and under-representation of cell types in an HPC. We used the K-S statistic $D_{n,m}$ to quantify for over-representation and under-representations, assigning $+D_{n,m}$ if there is over-representation (**A**) and $-D_{n,m}$ if there is under-representation (**B**). The Two-sample Kolmogorov-Smirnov test uses the statistic $D_{n,m}$ to quantify the distance between empirical distributions. The statistic is defined as $D_{n,m} = sup_x|F_{1,n}(x) - F_{2,m}|$ where $F_{1,n}(x), F_{2,m}$ are the empirical distribution functions of the first and second samples, n is the sample size for the F_1, m is the sample size for the F_2, and x the support for both distributions. Source data are provided as a Source Data file.

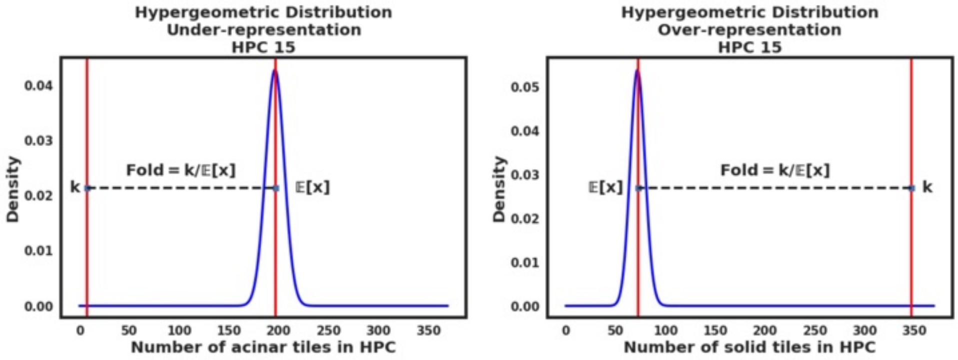

Supplementary Figure 33. Hypergeometric test for lung adenocarcinoma histological subtype enrichment. The hypergeometric test uses the hypergeometric distribution in order to measure the statistical significance of seeing k successes in n draws from a population of size N with K successes, without replacement. In our case, we specify k as the number of tiles with a particular histological subtype given as the number of total tiles in the HPC n, K is the number of tiles with the same particular histological subtype in the entire population of annotated tiles of size N. We measure enrichment by using a *fold* metric defined as $k/\mathbb{E}[x]$ where $\mathbb{E}[x]$ is the expectation of the hypergeometric distribution given n, K, N. Enrichment for a particular histological subtype shows as $fold > 1$ and depletion as $fold < 1$. This figure shows an example of the under-representation of acinar growth pattern and over-representation of the solid growth pattern in HPC 15. Source data are provided as a Source Data file.

Supplementary References

1. Chen, R. J. *et al.* Pan-cancer integrative histology-genomic analysis via multimodal deep learning. *Cancer Cell* **40**, 865–878 (2022).

2. Chen, R. J. *et al.* Scaling vision transformers to gigapixel images via hierarchical self-supervised learning. In *Proceedings of the IEEE/CVF Conference on Computer Vision and Pattern Recognition*, 16144–16155 (2022).

3. Thorsson, V. *et al.* The immune landscape of cancer. *Immunity* **48**, 812–830.e14 (2018).

4. Toccaceli, P. & Gammerman, A. Combination of inductive mondrian conformal predictors. *Mach. Learn.* **108**, 489–510 (2019).
