# Supplementary material for: Mapping the landscape of histomorphological cancer phenotypes using self-supervised learning on unannotated pathology slides
Source: Nat Commun. 2024 Jun 11;15:4596. doi: 10.1038/s41467-024-48666-7 (PMC11525555; doi:10.1038/s41467-024-48666-7)
Supplement: Supplementary file 3 — Reporting Summary [file 41467_2024_48666_MOESM3_ESM.pdf]

nature portfolio

Corresponding author(s): John Le Quesne, Aristotelis Tsirigos, Ke Yuan

Last updated by author(s): May 2, 2024

Reporting Summary

Nature Portfolio wishes to improve the reproducibility of the work that we publish. This form provides structure for consistency and transparency in reporting. For further information on Nature Portfolio policies, see our Editorial Policies and the Editorial Policy Checklist.

Statistics

For all statistical analyses, confirm that the following items are present in the figure legend, table legend, main text, or Methods section.

n/a	Confirmed	
☐	☒	The exact sample size (*n*) for each experimental group/condition, given as a discrete number and unit of measurement
☐	☒	A statement on whether measurements were taken from distinct samples or whether the same sample was measured repeatedly
☐	☒	The statistical test(s) used AND whether they are one- or two-sided<br>*Only common tests should be described solely by name; describe more complex techniques in the Methods section.*
☐	☒	A description of all covariates tested
☐	☒	A description of any assumptions or corrections, such as tests of normality and adjustment for multiple comparisons
☐	☒	A full description of the statistical parameters including central tendency (e.g. means) or other basic estimates (e.g. regression coefficient) AND variation (e.g. standard deviation) or associated estimates of uncertainty (e.g. confidence intervals)
☐	☒	For null hypothesis testing, the test statistic (e.g. *F*, *t*, *r*) with confidence intervals, effect sizes, degrees of freedom and *P* value noted<br>*Give P values as exact values whenever suitable.*
☒	☐	For Bayesian analysis, information on the choice of priors and Markov chain Monte Carlo settings
☒	☐	For hierarchical and complex designs, identification of the appropriate level for tests and full reporting of outcomes
☐	☒	Estimates of effect sizes (e.g. Cohen's *d*, Pearson's *r*), indicating how they were calculated

Our web collection on statistics for biologists contains articles on many of the points above.

Software and code

Policy information about availability of computer code

Data collection

Images were downloaded from the publicly available "TCGA" database (The Cancer Genome Atlas), together with associated labels (1054 Lung, 457 BLCA, 1133 BRCA, 279 CESC, 616 COAD, 449 PRAD, 475 SKCM, 442 STAD). Only those with immune signature information \cite{Thorsson2018} were kept. The immune landscape signatures for the TCGA samples are available at "Thorsson, V. et al. The immune landscape of cancer. Immunity" (https:/ /pubmed.ncbi.n lm. nih .gov /29628290/)

As an external cohort, both NYU_l and NYU_2 cohorts (138 and 276 LUAD slides respectively) were scanned at NYU by the Center of Biospecimen Research and Development core facility on a Aperio Scanner (Leica Biosystem) at 20x or 40x, and correspond to those previously used in
Coudray, Nicolas, et al. "Classification and mutation prediction from non-small cell lung cancer histopathology images using deep learning." Nature medicine 24.10 (2018): 1559-1567.
and
Moreira, Andre L., et al. "A grading system for invasive pulmonary adenocarcinoma: a proposal from the International Association for the Study of Lung Cancer Pathology Committee." Journal of Thoracic Oncology 15.10 (2020): 1599-1610.
The discrepancy with the original number of images used in those publications (140 and 300) corresponds to the removal of slides with:
- very low amount of tissues (100 tiles minimum)
- removing those with no tumor
- and selecting 1 random cases for patients with 2 slides (n=8)

For pre-processing, the code, python version and packages, and instructions for the image pre-processing is available at: https://github.com/ncoudray/DeepPATH

Data analysis	Code to replicate the results is available at: https://github.com/AdalbertoCq/Histomorphological-Phenotype-Learning<br>The repository provides instructions to replicate results, any output file from the methodology, and python version and packages versions.

For manuscripts utilizing custom algorithms or software that are central to the research but not yet described in published literature, software must be made available to editors and reviewers. We strongly encourage code deposition in a community repository (e.g. GitHub). See the Nature Portfolio guidelines for submitting code & software for further information.

Data

Policy information about availability of data

All manuscripts must include a data availability statement. This statement should provide the following information, where applicable:

- Accession codes, unique identifiers, or web links for publicly available datasets
- A description of any restrictions on data availability
- For clinical datasets or third party data, please ensure that the statement adheres to our policy

The Cancer Genome Atlas (TCGA) whole slide images and corresponding labels for the 10 cancer types (accessing IDs TCGA-LUAD, TCGA-LUSC, TCGA-BLCA, TCGA-BRCA, TCGA-CESC, TCGA-COAD, TCGA-PRAD, TCGA-SKCM, TCGA-STAD) are available at the Genomic Data Commons portal (https://gdc.cancer.gov/). This data is publicly available without restriction, authentication or authorization necessary. The immune landscape signatures for the TCGA samples are available at "Thorsson, V. et al. The immune landscape of cancer. Immunity"(https://pubmed.ncbi.nlm.nih.gov/29628290/) . Due to privacy, ethical considerations, and in accordance with the institutional policies, requests for whole slide images and corresponding labels in the additional New York University cohorts data may be addressed to the corresponding author, and a data transfer agreement between institutions will need to be signed - length and conditions for access will be defined by the parties following the procedure described in https://hslguides.med.nyu.edu/datasharing. The data generated in this study (pre-trained LUAD/LUSC model checkpoints, multi-cancer model checkpoints; tile vector representations for LUAD/LUSC before and after artefact removal; tile vector representation for the multi-cancer model; HPC configurations used in the publication for background and artefact removal, for LUAD/LUSC type classification, for LUAD survival and for multi-cancer analysis; whole slide image and patient vector representations for the lung subtype classification, the LUAD survival and the multi-cancer study; jupyter notebook to generate figures and results presented here) are available for download from the github page https://github.com/AdalbertoCq/Histomorphological-Phenotype-Learning.Source data are provided with this paper.

Research involving human participants, their data, or biological material

Policy information about studies with human participants or human data. See also policy information about sex, gender (identity/presentation), and sexual orientation and race, ethnicity and racism.

Reporting on sex and gender	This study was trained on public datasets which include both male and female participants.<br>Sex- and gender-based analyses were not performed in this study.
Reporting on race, ethnicity, or other socially relevant groupings	Race, ethnicity, or other socially relevant groupings information were not considered in the study .
Population characteristics	No such data were collected on NYU_2 dataset.<br><br>For NYU_1 dataset:<br>* 69% female, 31% male<br>* 86% white; 9% asian <2% black; ~3% other<br>* from 46 to 83 years old (avg=65)<br>* only stage I and II
Recruitment	The protocols of participant for TCGA recruitment could be found on the study websites (TCGA: https://portal.gdc.cancer.gov/).<br><br>For NYU cohort, these were stage I LUAD patients at NYU Langone Health. There are no apparent biases. Patients were not compensated.
Ethics oversight	Our research complies with all relevant ethical regulations, and the NYU specimens used for this were collected under the NCI/NIH Early Detection Research Network U01CA214195 to Harvey I. Pass MD. NYU slides for this investigation were used according to protocol i8896 ``The Lung Cancer Biomarker Center'', H. Pass, co-investigator, which was approved by the New York University Langone Health Investigational Review Board on a yearly basis since 2001. All patients signed written informed consent for the use of their tissues, blood, and slides as well as for the use of corresponding de-identified data, as well as permission to have follow-up by the Principle Investigator.

Note that full information on the approval of the study protocol must also be provided in the manuscript.

Field-specific reporting

Please select the one below that is the best fit for your research. If you are not sure, read the appropriate sections before making your selection.

☒ Life sciences ☐ Behavioural & social sciences ☐ Ecological, evolutionary & environmental sciences

For a reference copy of the document with all sections, see nature.com/documents/nr-reporting-summary-flat.pdf

Life sciences study design

All studies must disclose on these points even when the disclosure is negative.

Sample size	Sample size was determined based on the number of cases available in the databases mined. The TCGA cohort is composed of 1021 WSIs, 513 of adenocarcinoma (LUAD), 508 WSIs of squamous cell carcinoma (LUSC). The external dataset used in the lung type classification is composed of 138 WSIs, 72 of LUAD and 66 of LUSC. The second external dataset used in the LUAD overall and recurrence free survival is composed of 276 patients.<br>Our multi-cancer analysis used 10 cancer types from The Cancer Genome Atlas (TCGA). The complete cohort is composed of 279 patients of bladder urothelial carcinoma (BLCA), 364 patients of breast invasive carcinoma (BRCA), 187 patients of cervical squamous cell carcinoma and endocervical adenocarcinoma (CESC), 369 patients of colon adenocarcinoma (COAD), 366 patients of lung adenocarcinoma (LUAD), 367 patients of lung squamous cell carcinoma (LUSC), 247 patients of prostate adenocarcinoma (PRAD), 363 patients of skin cutaneous melanoma (SKCM), 278 patients of stomach adenocarcinoma (STAD), and 395 patients of uterine corpus endometrial carcinoma (UCEC).
Data exclusions	To keep the external cohort homogeneous, we made sure we had only 1 slide per patient. In the NYU_2 cohorts, we had only 8 patients with 2 slides and randomly selected 1
Replication	5-fold cross-validation experiments were run for all tests.
Randomization	Training, validation, and test sets were created through random sampling of the TCGA dataset.
Blinding	Histological Assessment of cluster was done in a manner blinded to patient outcomes.

Reporting for specific materials, systems and methods

We require information from authors about some types of materials, experimental systems and methods used in many studies. Here, indicate whether each material, system or method listed is relevant to your study. If you are not sure if a list item applies to your research, read the appropriate section before selecting a response.

Materials & experimental systems

n/a	Involved in the study
☒	☐ Antibodies
☒	☐ Eukaryotic cell lines
☒	☐ Palaeontology and archaeology
☒	☐ Animals and other organisms
☒	☐ Clinical data
☒	☐ Dual use research of concern
☒	☐ Plants

Methods

n/a	Involved in the study
☒	☐ ChIP-seq
☒	☐ Flow cytometry
☒	☐ MRI-based neuroimaging

Plants

Seed stocks	*Report on the source of all seed stocks or other plant material used. If applicable, state the seed stock centre and catalogue number. If plant specimens were collected from the field, describe the collection location, date and sampling procedures.*
Novel plant genotypes	*Describe the methods by which all novel plant genotypes were produced. This includes those generated by transgenic approaches, gene editing, chemical/radiation-based mutagenesis and hybridization. For transgenic lines, describe the transformation method, the number of independent lines analyzed and the generation upon which experiments were performed. For gene-edited lines, describe the editor used, the endogenous sequence targeted for editing, the targeting guide RNA sequence (if applicable) and how the editor was applied.*
Authentication	*Describe any authentication procedures for each seed stock used or novel genotype generated. Describe any experiments used to assess the effect of a mutation and, where applicable, how potential secondary effects (e.g. second site T-DNA insertions, mosiacism, off-target gene editing) were examined.*
