# Supplementary figures and images for: Mapping the landscape of histomorphological cancer phenotypes using self-supervised learning on unannotated pathology slides
Source: Nat Commun. 2024 Jun 11;15:4596. doi: 10.1038/s41467-024-48666-7 (PMC11525555; doi:10.1038/s41467-024-48666-7)

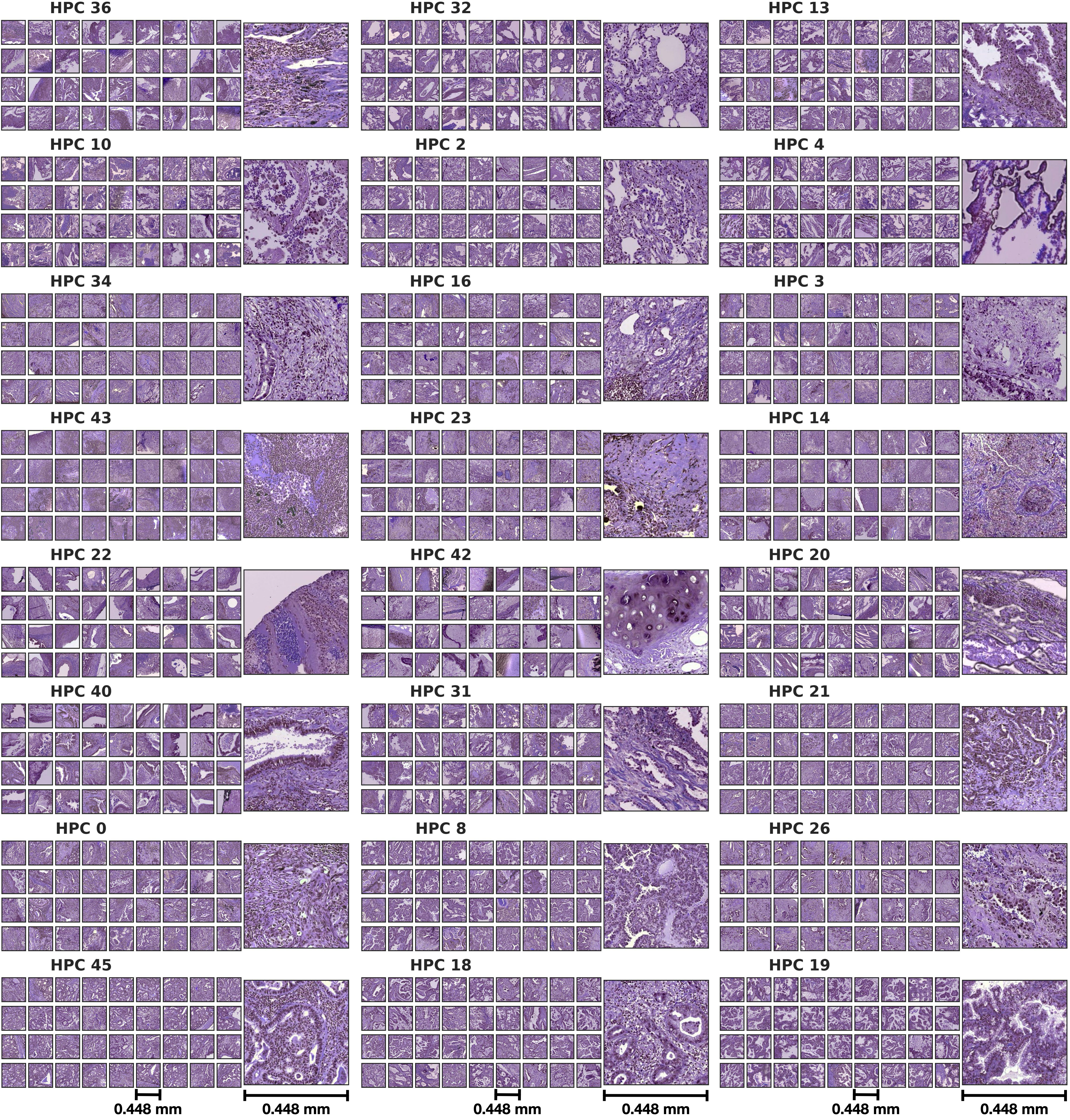

Supplement: Supplementary file 4 — Source Data [file 41467_2024_48666_MOESM4_ESM.zip › Source_Data/paper_tissue_figures/SupplementaryFigure3.jpg]

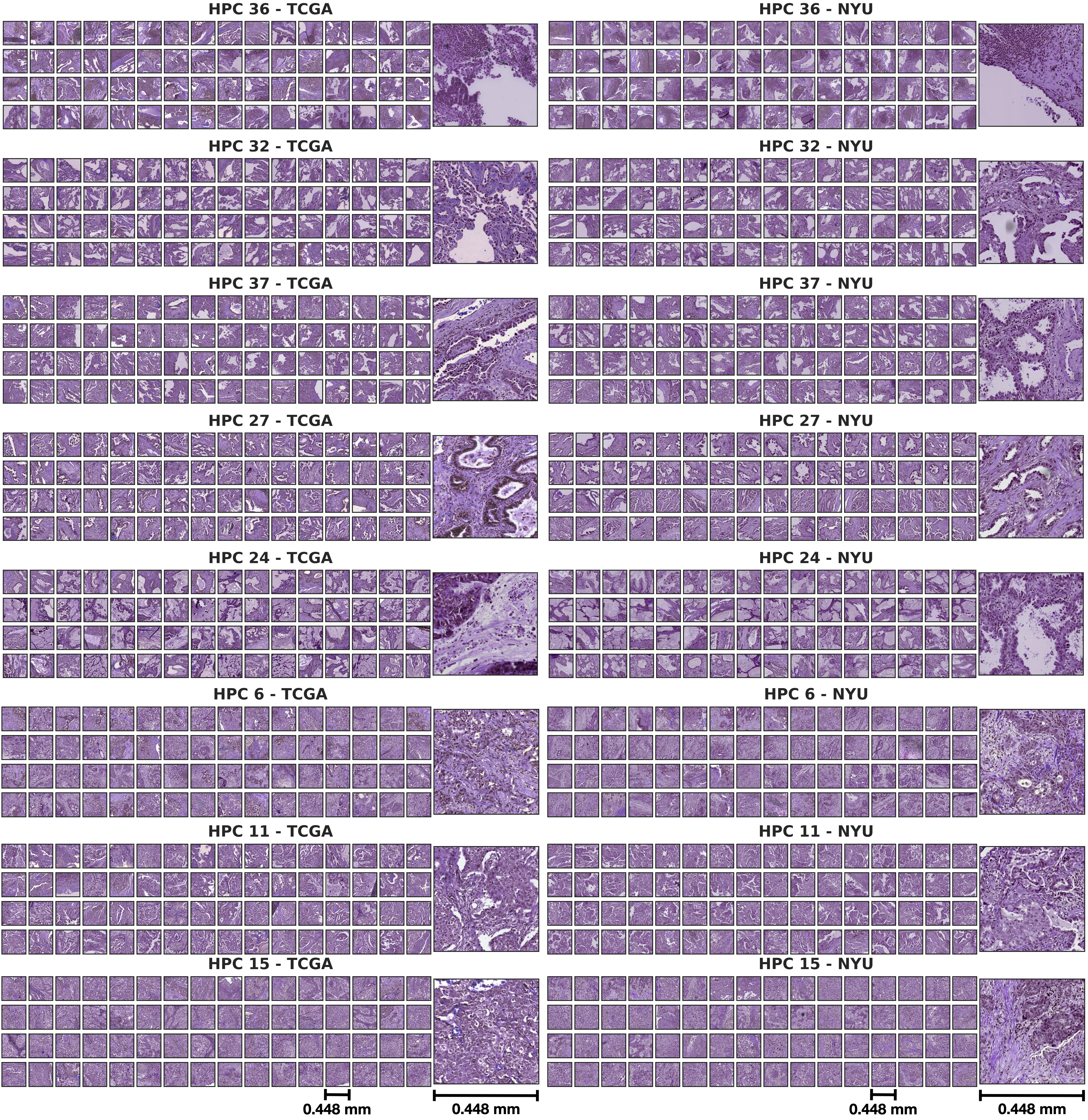

Supplement: Supplementary file 4 — Source Data [file 41467_2024_48666_MOESM4_ESM.zip › Source_Data/paper_tissue_figures/SupplementaryFigure11.jpg]

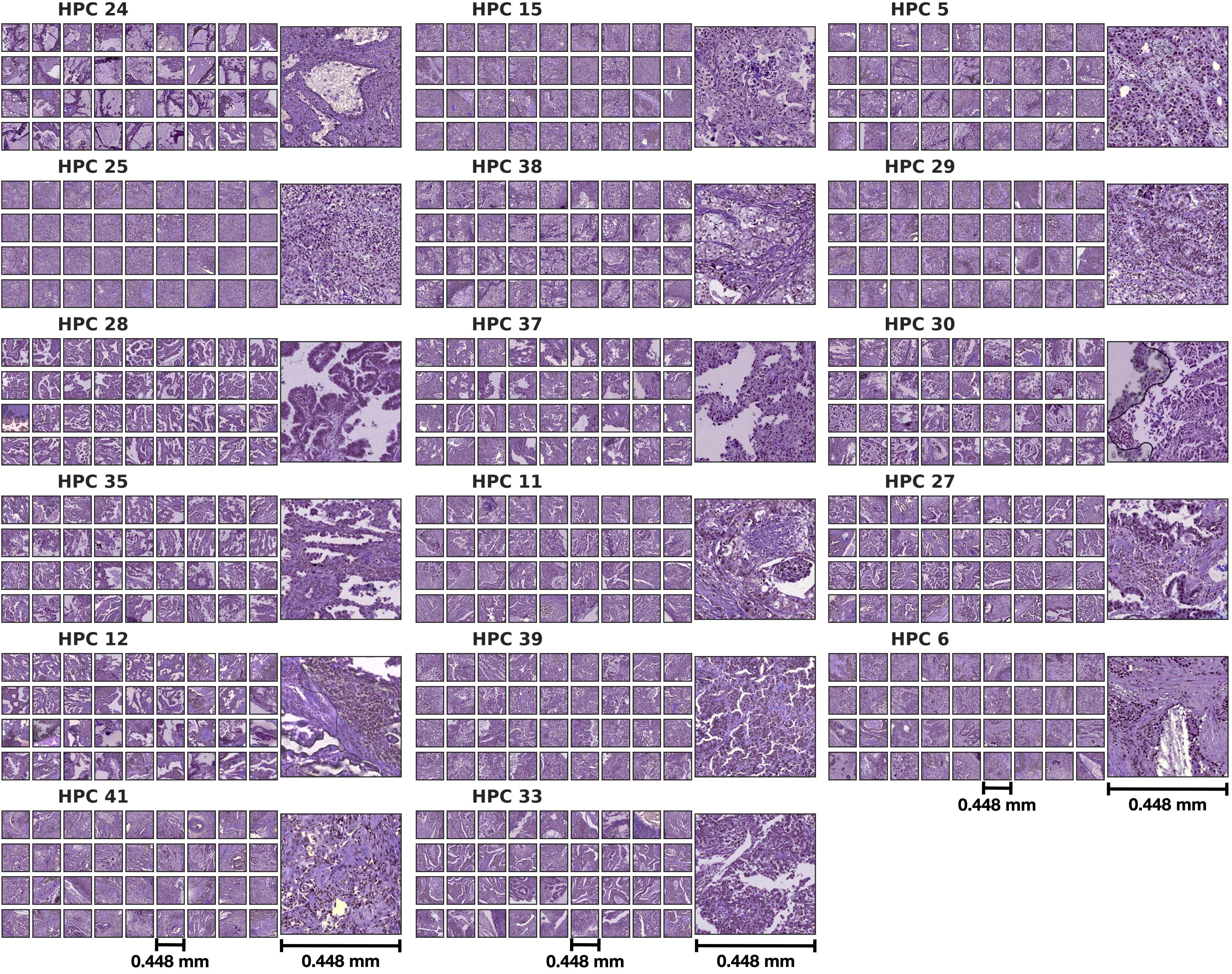

Supplement: Supplementary file 4 — Source Data [file 41467_2024_48666_MOESM4_ESM.zip › Source_Data/paper_tissue_figures/SupplementaryFigure4.jpg]

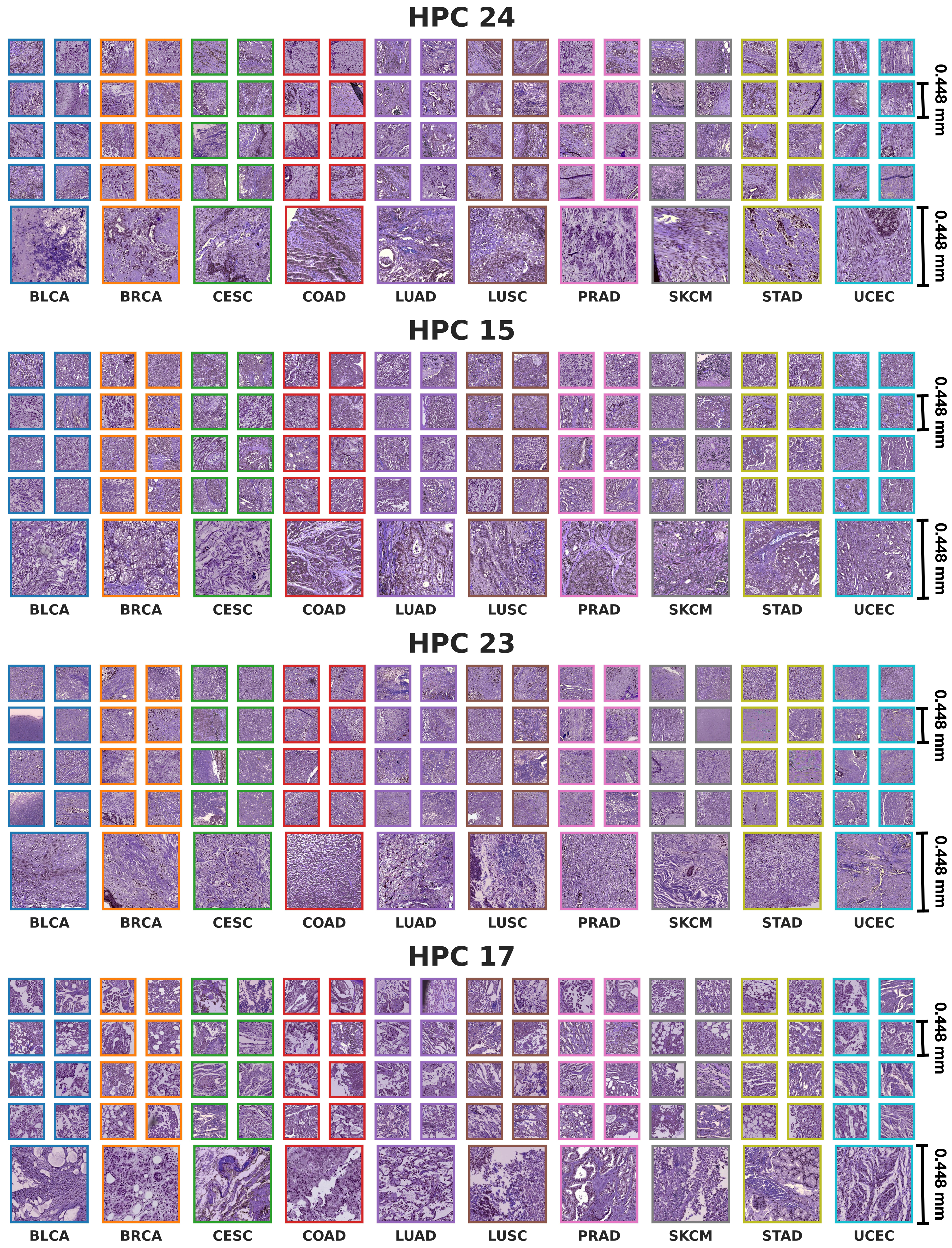

Supplement: Supplementary file 4 — Source Data [file 41467_2024_48666_MOESM4_ESM.zip › Source_Data/paper_tissue_figures/SupplementaryFigure28.jpg]

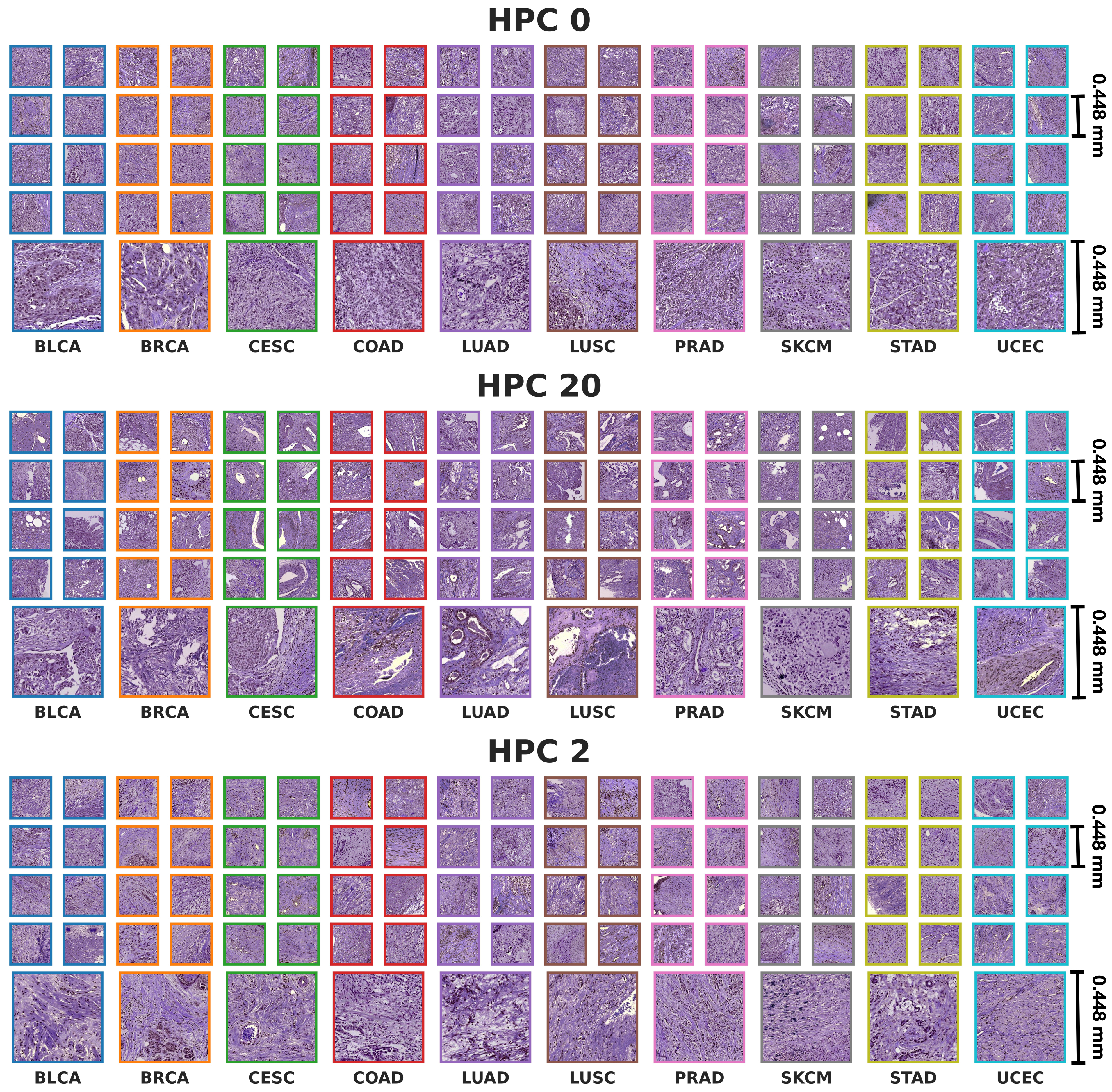

Supplement: Supplementary file 4 — Source Data [file 41467_2024_48666_MOESM4_ESM.zip › Source_Data/paper_tissue_figures/SupplementaryFigure29.jpg]

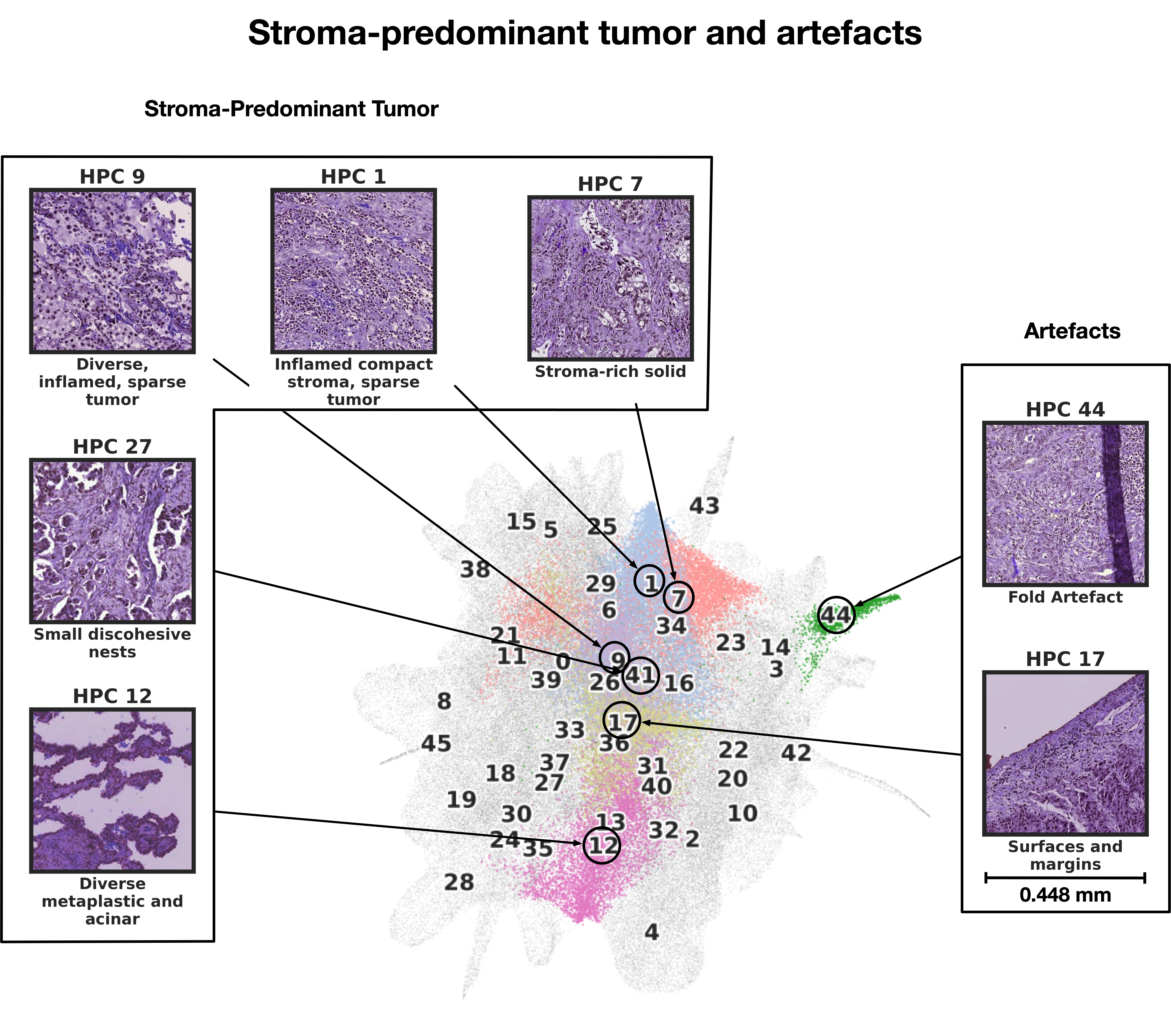

Supplement: Supplementary file 4 — Source Data [file 41467_2024_48666_MOESM4_ESM.zip › Source_Data/paper_tissue_figures/SupplementaryFigure5.jpg]

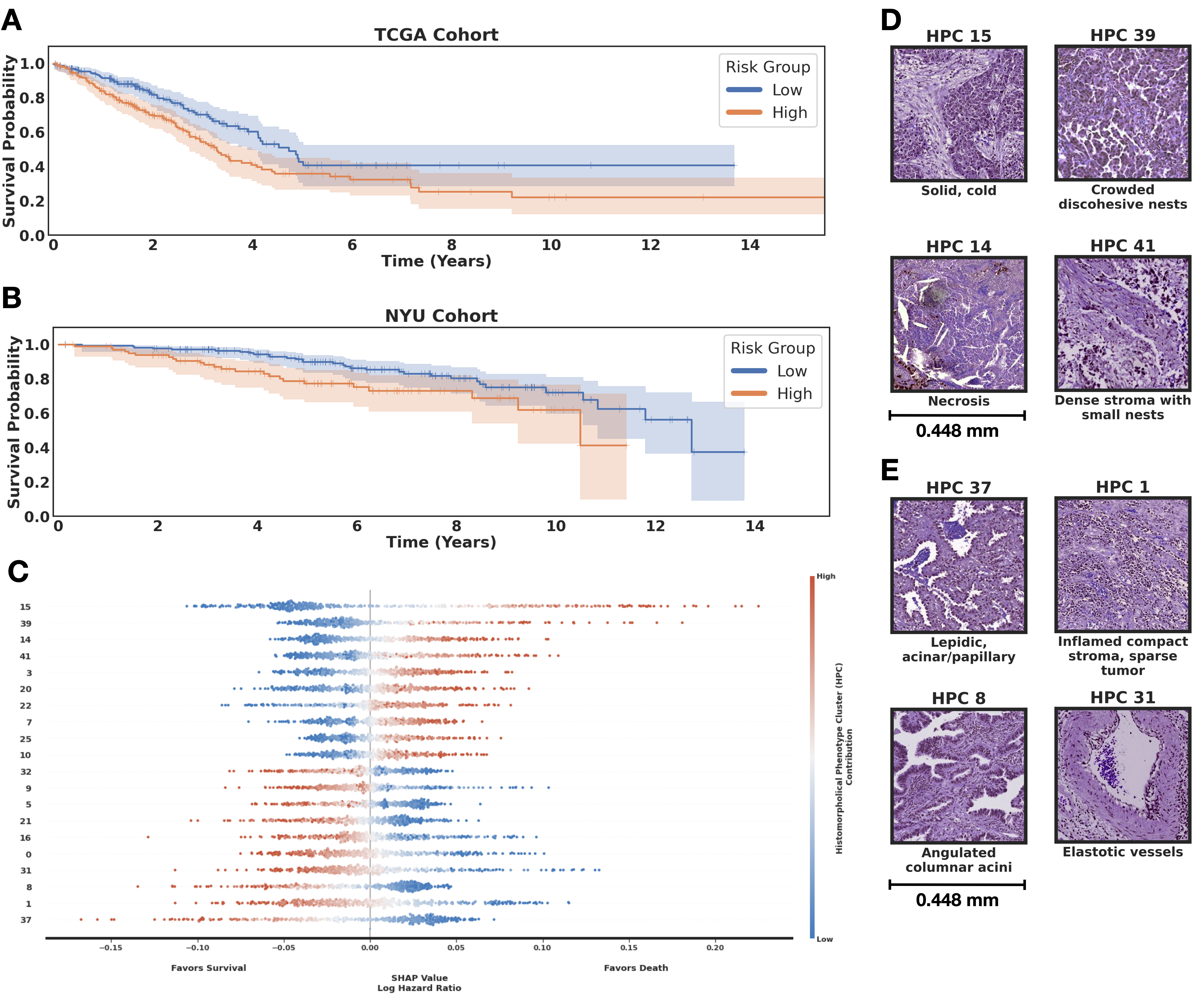

Supplement: Supplementary file 4 — Source Data [file 41467_2024_48666_MOESM4_ESM.zip › Source_Data/paper_tissue_figures/SupplementaryFigure6.jpg]

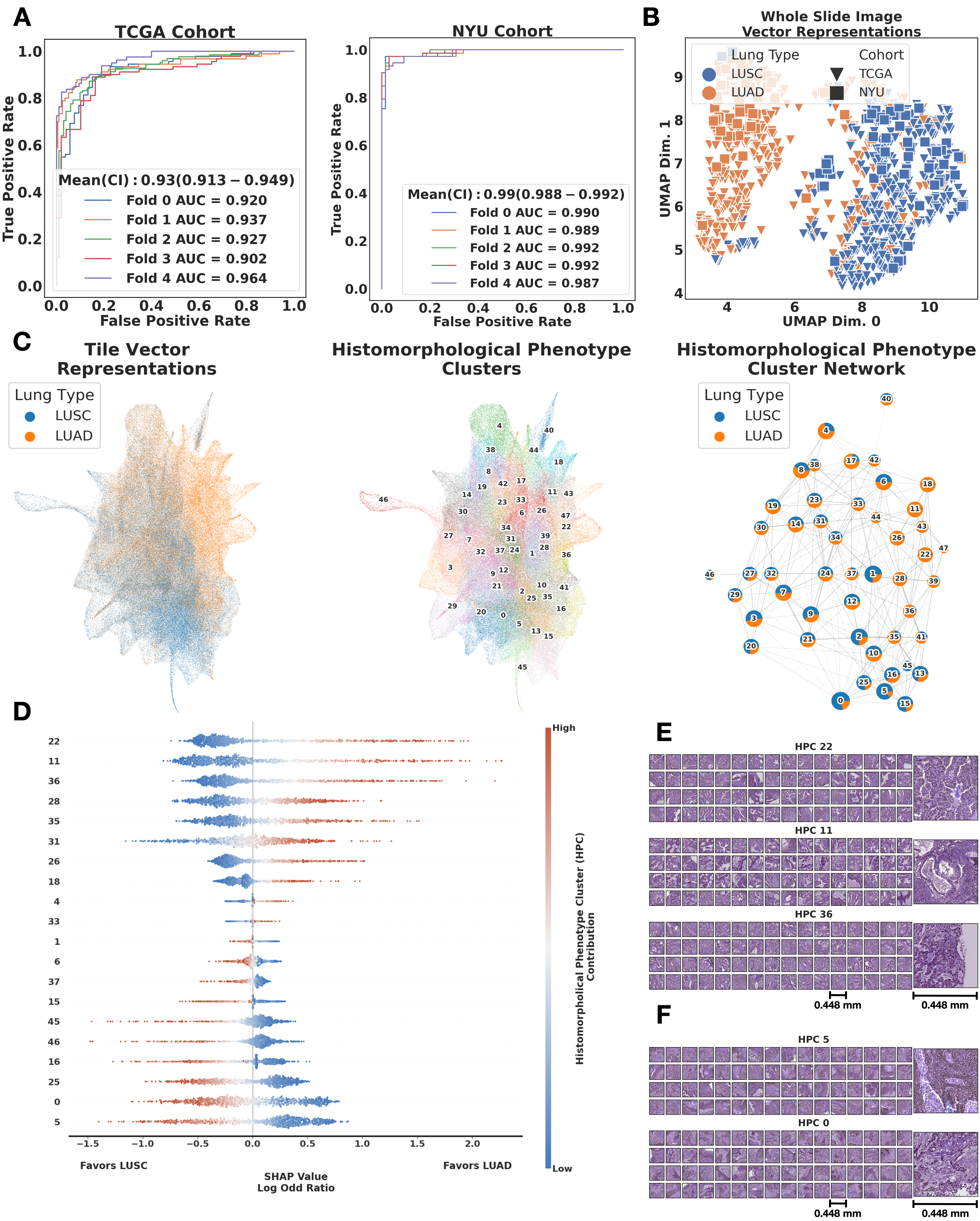

Supplement: Supplementary file 4 — Source Data [file 41467_2024_48666_MOESM4_ESM.zip › Source_Data/paper_tissue_figures/SupplementaryFigure16.jpg]

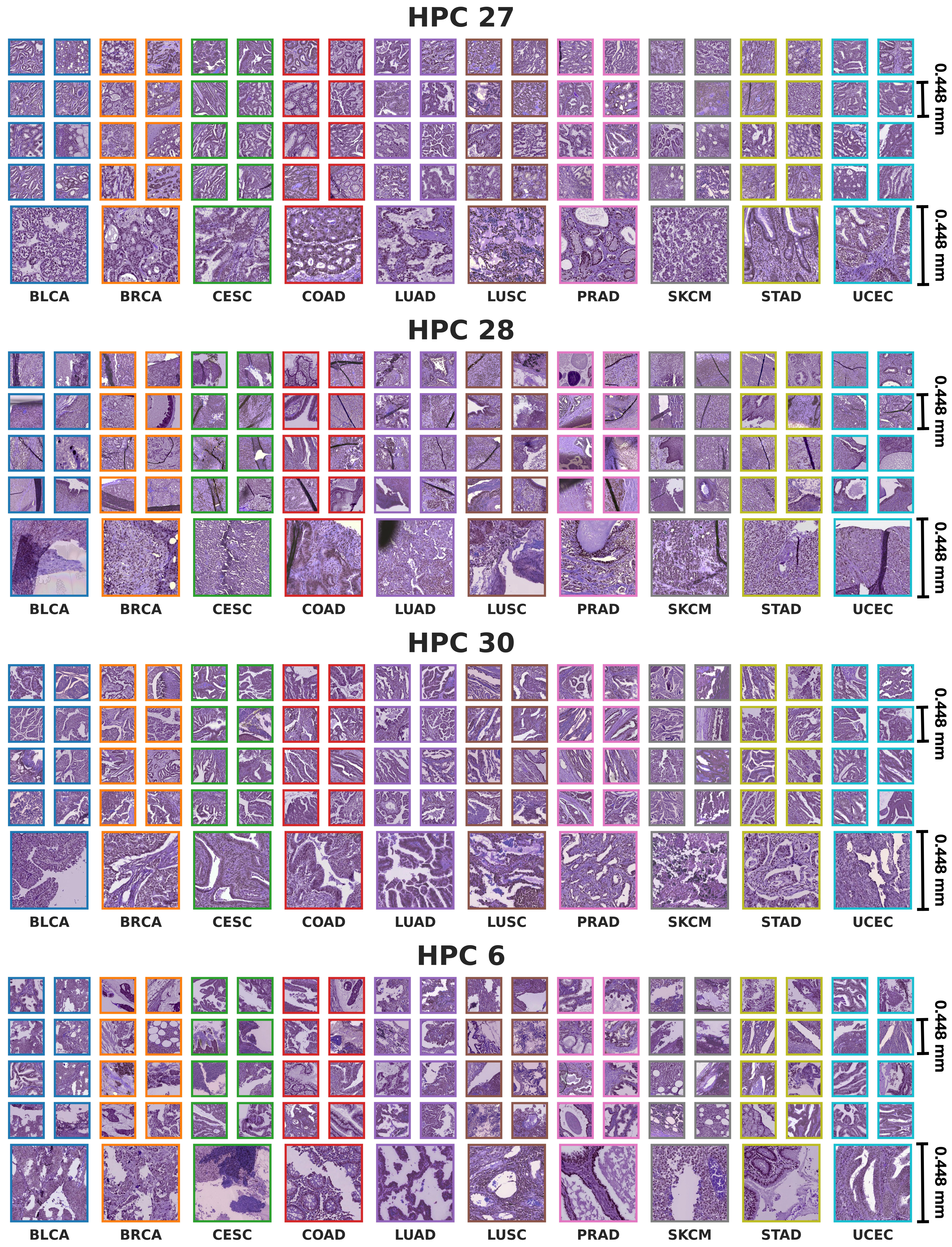

Supplement: Supplementary file 4 — Source Data [file 41467_2024_48666_MOESM4_ESM.zip › Source_Data/paper_tissue_figures/SupplementaryFigure27.jpg]

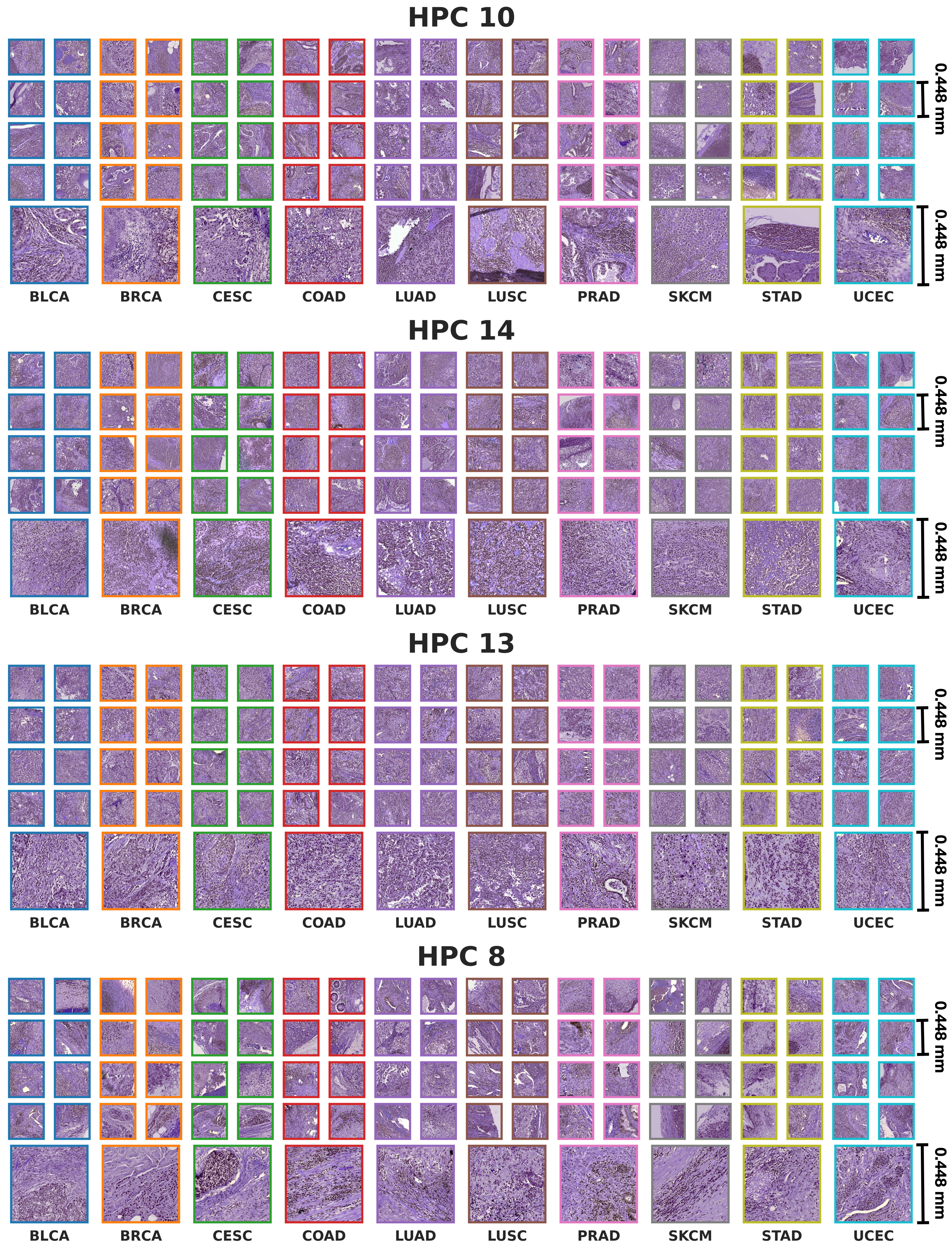

Supplement: Supplementary file 4 — Source Data [file 41467_2024_48666_MOESM4_ESM.zip › Source_Data/paper_tissue_figures/SupplementaryFigure24.jpg]

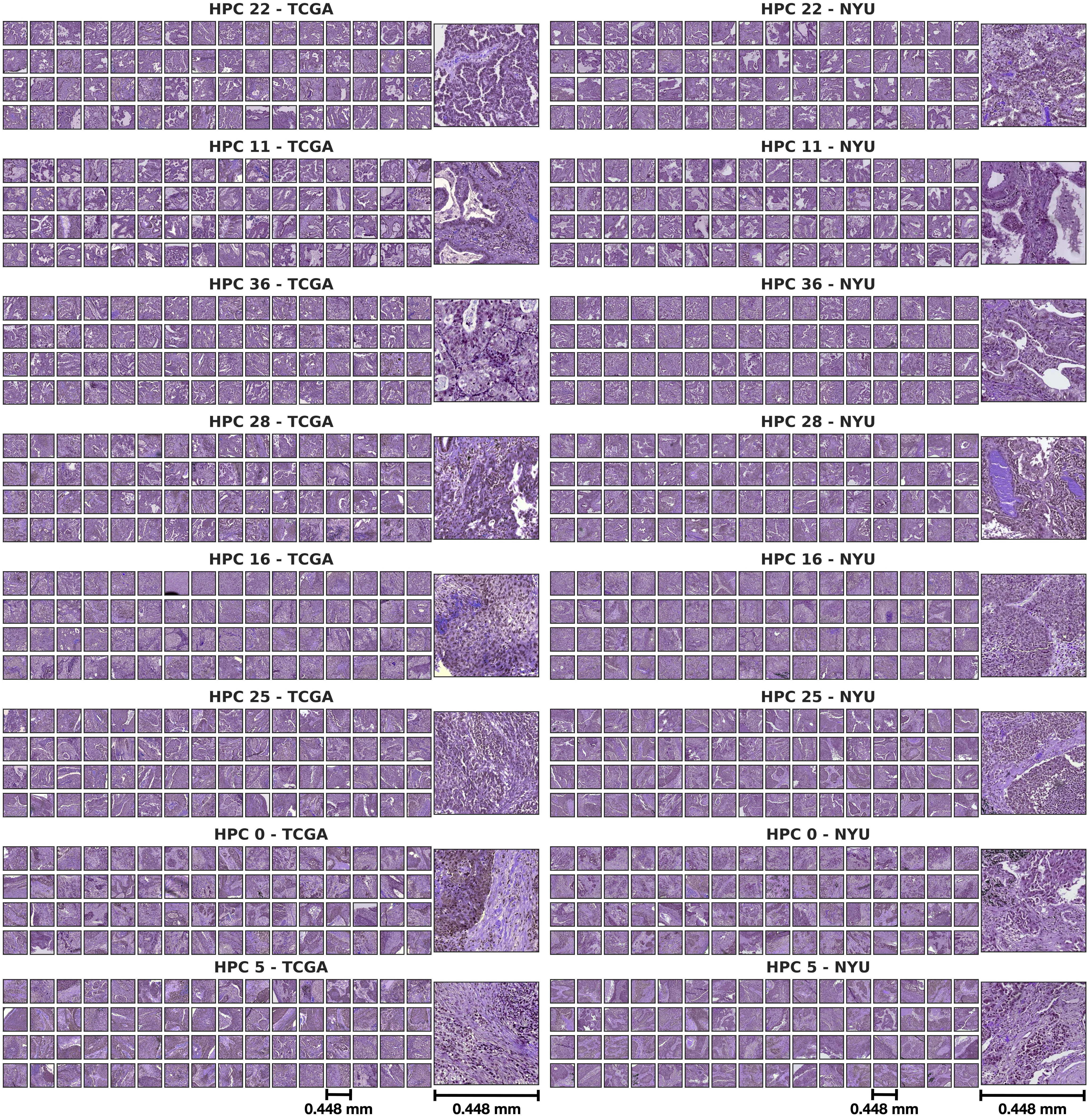

Supplement: Supplementary file 4 — Source Data [file 41467_2024_48666_MOESM4_ESM.zip › Source_Data/paper_tissue_figures/SupplementaryFigure9.jpg]
