# Supplementary material for: Mapping the landscape of histomorphological cancer phenotypes using self-supervised learning on unannotated pathology slides
Source: Nat Commun. 2024 Jun 11;15:4596. doi: 10.1038/s41467-024-48666-7 (PMC11525555; doi:10.1038/s41467-024-48666-7)
Supplement: Supplementary file 4 — Source Data [file Figure1.pdf › Source_Data/paper_tissue_figures/Figure1.pdf]

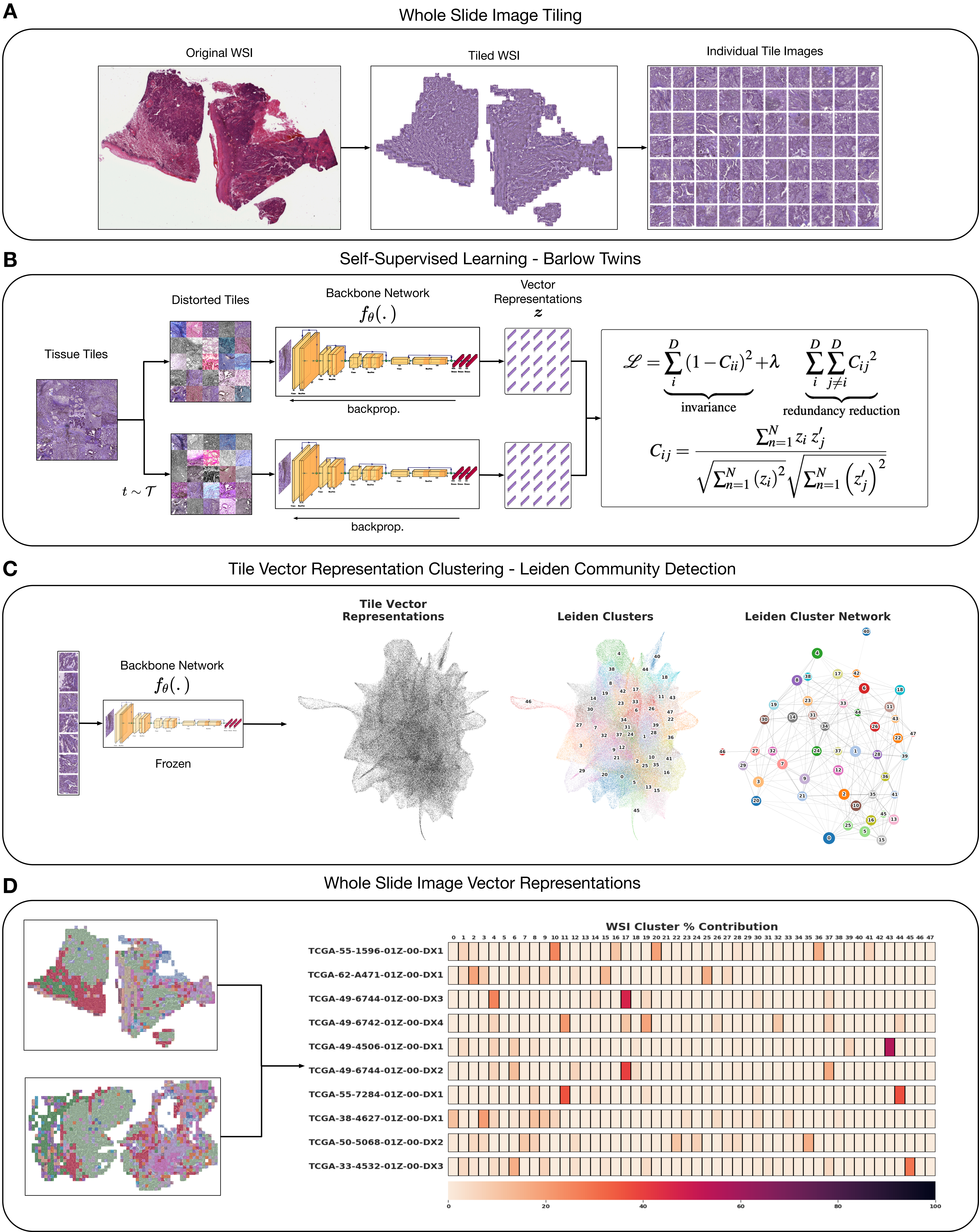
A
Whole Slide Image Tiling
Original WSI
Tiled WSI
Individual Tile Images
B
Self-Supervised Learning - Barlow Twins
Tissue Tiles
Distorted Tiles
$t \sim \mathcal{T}$
Backbone Network
$f_\theta(.)$
Vector Representations
z
backprop.
backprop.
$$\mathcal{L} = \underbrace{\sum_{i}^{D}(1 - C_{ii})^2}_{\text{invariance}} + \lambda \underbrace{\sum_{i}^{D}\sum_{j \neq i}^{D} {C_{ij}}^2}_{\text{redundancy reduction}}$$
$$C_{ij} = \frac{\sum_{n=1}^{N} z_i\, z_j'}{\sqrt{\sum_{n=1}^{N} (z_i)^2}\sqrt{\sum_{n=1}^{N} \left(z_j'\right)^2}}$$
C
Tile Vector Representation Clustering - Leiden Community Detection
Backbone Network
$f_\theta(.)$
Frozen
Tile Vector Representations
Leiden Clusters
Leiden Cluster Network
D
Whole Slide Image Vector Representations
WSI Cluster % Contribution
0 1 2 3 4 5 6 7 8 9 10 11 12 13 14 15 16 17 18 19 20 21 22 23 24 25 26 27 28 29 30 31 32 33 34 35 36 37 38 39 40 41 42 43 44 45 46 47
TCGA-55-1596-01Z-00-DX1
TCGA-62-A471-01Z-00-DX1
TCGA-49-6744-01Z-00-DX3
TCGA-49-6742-01Z-00-DX4
TCGA-49-4506-01Z-00-DX1
TCGA-49-6744-01Z-00-DX2
TCGA-55-7284-01Z-00-DX1
TCGA-38-4627-01Z-00-DX1
TCGA-50-5068-01Z-00-DX2
TCGA-33-4532-01Z-00-DX3
0 20 40 60 80 100
