# Supplementary material for: Mapping the landscape of histomorphological cancer phenotypes using self-supervised learning on unannotated pathology slides
Source: Nat Commun. 2024 Jun 11;15:4596. doi: 10.1038/s41467-024-48666-7 (PMC11525555; doi:10.1038/s41467-024-48666-7)
Supplement: Supplementary file 4 — Source Data [file Figure7.pdf › Source_Data/paper_tissue_figures/Figure7.pdf]

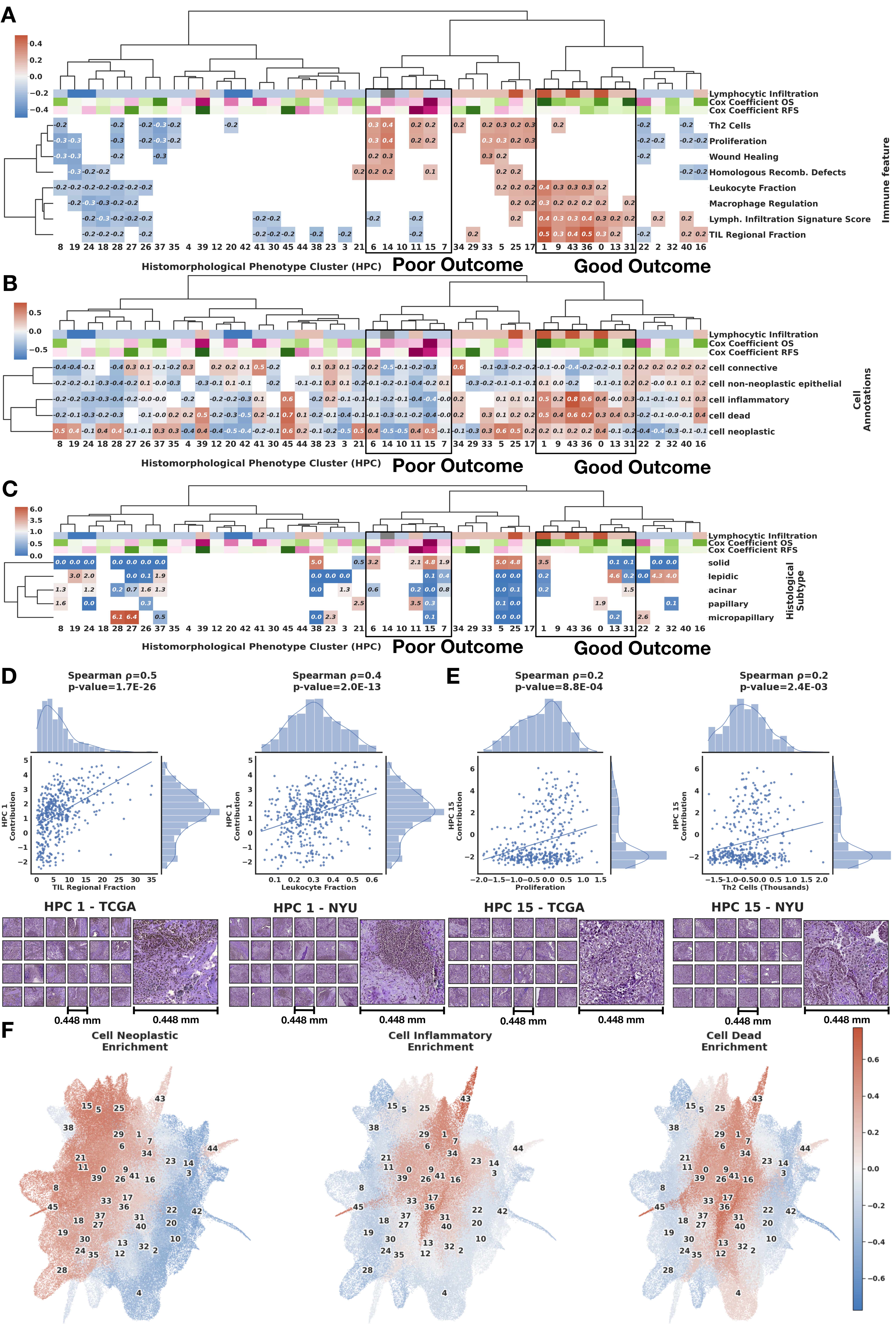
A
Lymphocytic Infiltration
Cox Coefficient OS
Cox Coefficient RFS
Th2 Cells
Proliferation
Wound Healing
Homologous Recomb. Defects
Leukocyte Fraction
Macrophage Regulation
Lymph. Infiltration Signature Score
TIL Regional Fraction
Immune feature
8 19 24 18 28 27 26 37 35 4 39 12 20 42 41 30 45 44 38 23 3 21 6 14 10 11 15 7 34 29 33 5 25 17 1 9 43 36 0 13 31 22 2 32 40 16
Histomorphological Phenotype Cluster (HPC)
Poor Outcome
Good Outcome
B
cell connective
cell non-neoplastic epithelial
cell inflammatory
cell dead
cell neoplastic
Cell Annotations
C
solid
lepidic
acinar
papillary
micropapillary
Histological Subtype
D
Spearman ρ=0.5
p-value=1.7E-26
HPC 1 Contribution
TIL Regional Fraction
Spearman ρ=0.4
p-value=2.0E-13
Leukocyte Fraction
E
Spearman ρ=0.2
p-value=8.8E-04
HPC 15 Contribution
Proliferation
Spearman ρ=0.2
p-value=2.4E-03
Th2 Cells (Thousands)
HPC 1 - TCGA
HPC 1 - NYU
HPC 15 - TCGA
HPC 15 - NYU
0.448 mm
F
Cell Neoplastic Enrichment
Cell Inflammatory Enrichment
Cell Dead Enrichment
