# Supplementary material for: Mapping the landscape of histomorphological cancer phenotypes using self-supervised learning on unannotated pathology slides
Source: Nat Commun. 2024 Jun 11;15:4596. doi: 10.1038/s41467-024-48666-7 (PMC11525555; doi:10.1038/s41467-024-48666-7)
Supplement: Supplementary file 4 — Source Data [file Figure6.pdf › Source_Data/paper_tissue_figures/Figure6.pdf]

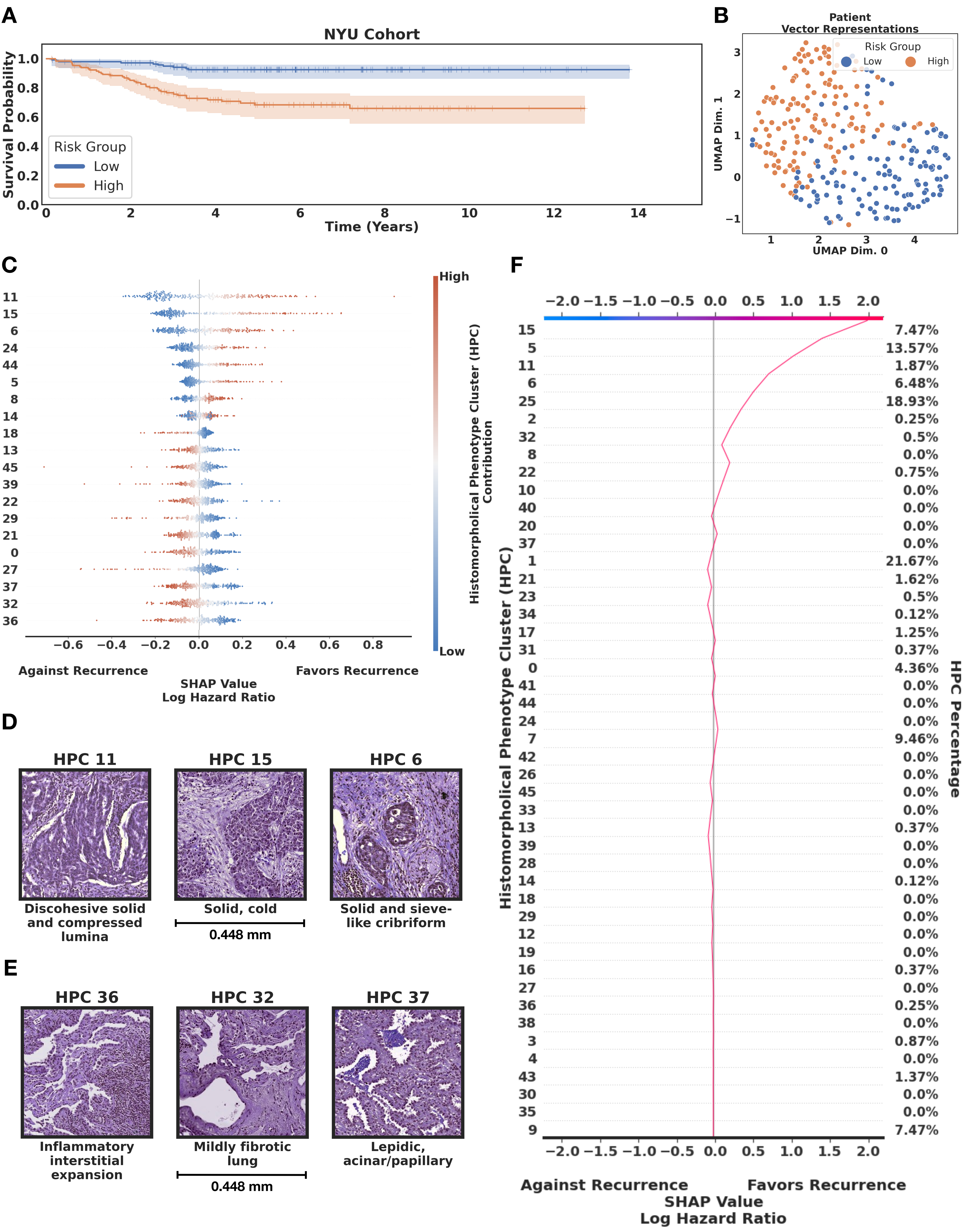
A
NYU Cohort
Survival Probability
Time (Years)
Risk Group
Low
High
B
Patient
Vector Representations
Risk Group
Low
High
UMAP Dim. 1
UMAP Dim. 0
C
High
Low
Histomorphological Phenotype Cluster (HPC)
Contribution
Against Recurrence
Favors Recurrence
SHAP Value
Log Hazard Ratio
D
HPC 11
Discohesive solid and compressed lumina
HPC 15
Solid, cold
0.448 mm
HPC 6
Solid and sieve-like cribriform
E
HPC 36
Inflammatory interstitial expansion
HPC 32
Mildly fibrotic lung
0.448 mm
HPC 37
Lepidic, acinar/papillary
F
Histomorphological Phenotype Cluster (HPC)
HPC Percentage
15 7.47%
5 13.57%
11 1.87%
6 6.48%
25 18.93%
2 0.25%
32 0.5%
8 0.0%
22 0.75%
10 0.0%
40 0.0%
20 0.0%
37 0.0%
1 21.67%
21 1.62%
23 0.5%
34 0.12%
17 1.25%
31 0.37%
0 4.36%
41 0.0%
44 0.0%
24 0.0%
7 9.46%
42 0.0%
26 0.0%
45 0.0%
33 0.0%
13 0.37%
39 0.0%
28 0.0%
14 0.12%
18 0.0%
29 0.0%
12 0.0%
19 0.0%
16 0.37%
27 0.0%
36 0.25%
38 0.0%
3 0.87%
4 0.0%
43 1.37%
30 0.0%
35 0.0%
9 7.47%
Against Recurrence
Favors Recurrence
SHAP Value
Log Hazard Ratio
