# Supplementary material for: Mapping the landscape of histomorphological cancer phenotypes using self-supervised learning on unannotated pathology slides
Source: Nat Commun. 2024 Jun 11;15:4596. doi: 10.1038/s41467-024-48666-7 (PMC11525555; doi:10.1038/s41467-024-48666-7)

A. Classical adenocarcinoma appearances

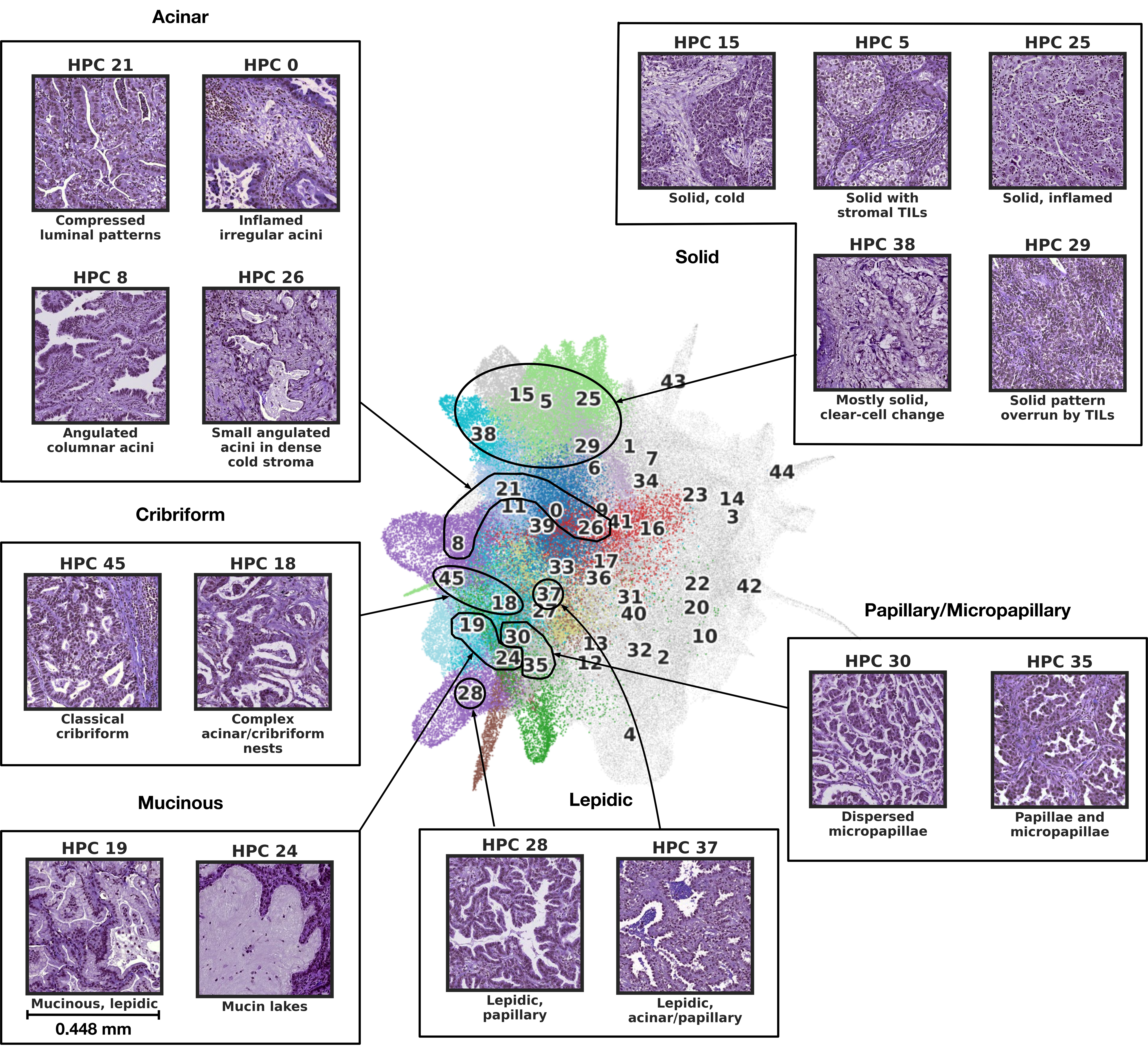

B. Variant adenocarcinoma appearances

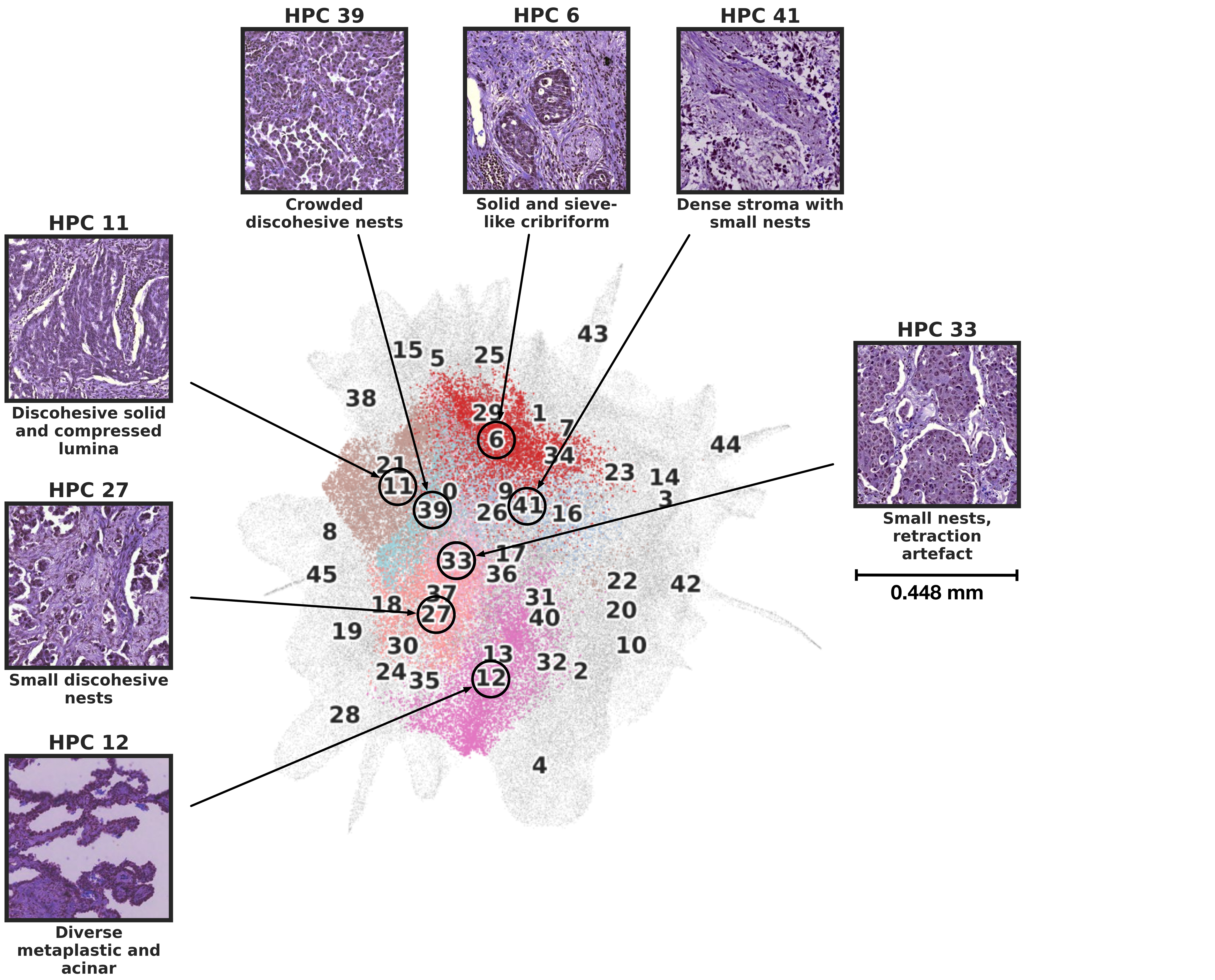

Supplement: Supplementary file 4 — Source Data [file Figure4.pdf › Source_Data/paper_tissue_figures/Figure4.pdf]
