# Supplementary material for: Mapping the landscape of histomorphological cancer phenotypes using self-supervised learning on unannotated pathology slides
Source: Nat Commun. 2024 Jun 11;15:4596. doi: 10.1038/s41467-024-48666-7 (PMC11525555; doi:10.1038/s41467-024-48666-7)
Supplement: Supplementary file 4 — Source Data [file Figure5.pdf › Source_Data/paper_tissue_figures/Figure5.pdf]

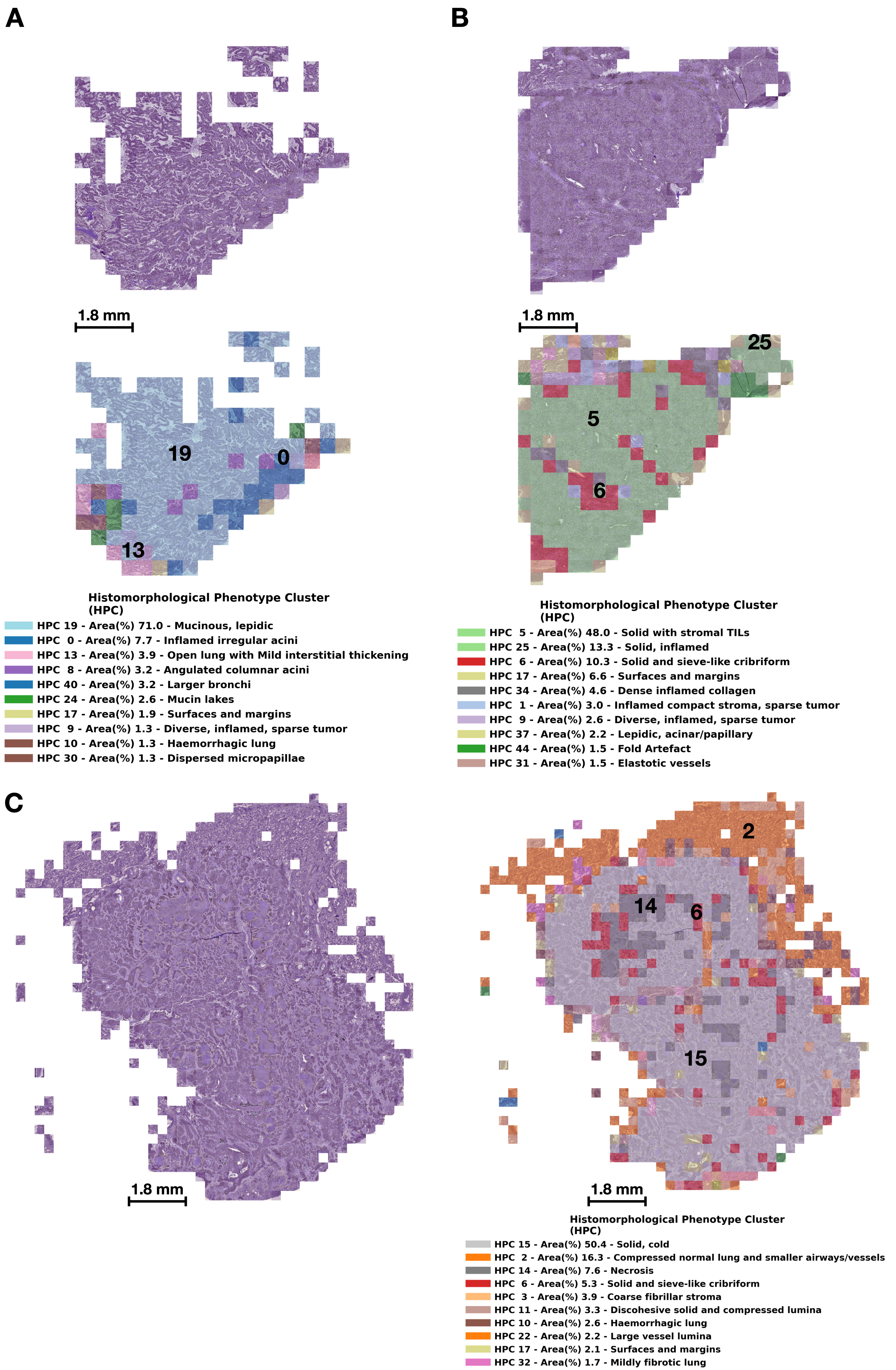
A
1.8 mm
19
0
13
Histomorphological Phenotype Cluster (HPC)
HPC 19 - Area(%) 71.0 - Mucinous, lepidic
HPC 0 - Area(%) 7.7 - Inflamed irregular acini
HPC 13 - Area(%) 3.9 - Open lung with Mild interstitial thickening
HPC 8 - Area(%) 3.2 - Angulated columnar acini
HPC 40 - Area(%) 3.2 - Larger bronchi
HPC 24 - Area(%) 2.6 - Mucin lakes
HPC 17 - Area(%) 1.9 - Surfaces and margins
HPC 9 - Area(%) 1.3 - Diverse, inflamed, sparse tumor
HPC 10 - Area(%) 1.3 - Haemorrhagic lung
HPC 30 - Area(%) 1.3 - Dispersed micropapillae
B
1.8 mm
25
5
6
Histomorphological Phenotype Cluster (HPC)
HPC 5 - Area(%) 48.0 - Solid with stromal TILs
HPC 25 - Area(%) 13.3 - Solid, inflamed
HPC 6 - Area(%) 10.3 - Solid and sieve-like cribriform
HPC 17 - Area(%) 6.6 - Surfaces and margins
HPC 34 - Area(%) 4.6 - Dense inflamed collagen
HPC 1 - Area(%) 3.0 - Inflamed compact stroma, sparse tumor
HPC 9 - Area(%) 2.6 - Diverse, inflamed, sparse tumor
HPC 37 - Area(%) 2.2 - Lepidic, acinar/papillary
HPC 44 - Area(%) 1.5 - Fold Artefact
HPC 31 - Area(%) 1.5 - Elastotic vessels
C
1.8 mm
2
14
6
15
1.8 mm
Histomorphological Phenotype Cluster (HPC)
HPC 15 - Area(%) 50.4 - Solid, cold
HPC 2 - Area(%) 16.3 - Compressed normal lung and smaller airways/vessels
HPC 14 - Area(%) 7.6 - Necrosis
HPC 6 - Area(%) 5.3 - Solid and sieve-like cribriform
HPC 3 - Area(%) 3.9 - Coarse fibrillar stroma
HPC 11 - Area(%) 3.3 - Discohesive solid and compressed lumina
HPC 10 - Area(%) 2.6 - Haemorrhagic lung
HPC 22 - Area(%) 2.2 - Large vessel lumina
HPC 17 - Area(%) 2.1 - Surfaces and margins
HPC 32 - Area(%) 1.7 - Mildly fibrotic lung
